# Impact of social factors and health campaigns on the burden of idiopathic epilepsy: an inequality, decomposition, generalized and synthetic difference-in-differences study

**DOI:** 10.3389/fpubh.2025.1598497

**Published:** 2025-06-02

**Authors:** Xuewen Rong, Dongting Yu, Wei Zhao, Jingyu Xiao, Du Feng, Gengfeng Chen, Zemeng Cao, Liming Shu

**Affiliations:** 1 Department of Neurology, Institute of Neuroscience, Key Laboratory of Neurogenetics and Channelopathies of Guangdong Province and the Ministry of Education of China, The Second Affiliated Hospital, Guangzhou Medical University, Guangzhou, China; 2 Nanshan School, The Second Affiliated Hospital, Guangzhou Medical University, Guangzhou, China; 3 School of Pediatrics, Guangzhou Medical University, Guangzhou, China

**Keywords:** idiopathic epilepsy, disability-adjusted life years, generalized difference-in-differences, synthetic difference-in-differences, inequality, global burden of disease

## Abstract

**Background:**

Idiopathic epilepsy is a major global health burden that causes premature death and disability. Previous studies have systematically analyzed trends in the burden of idiopathic epilepsy. However, analyses of the impact of social factors, health campaigns and policies on the burden of idiopathic epilepsy are lacking. This study aims to fill that gap.

**Methods:**

We used data from the 2021 Global Burden of Disease (GBD) and calculated the estimated annual percentage changes (EAPC) to assess trends. Decomposition analysis breaks down changes in the burden of idiopathic epilepsy into three factors. Correlation analysis measures the association between the EAPC and social development. Inequality analysis illustrates the disparity in the burden of idiopathic epilepsy among countries. Generalized difference-in-differences (GDID) and synthetic difference-in-differences (SDID) analyses are used to quantify the impact of health campaigns or policies on the burden of idiopathic epilepsy.

**Results:**

With respect to incidence, aging had a negative impact. The correlation between the sociodemographic index (SDI) and EAPC had a rho value of −0.18. The slope index of inequality for the mortality burden decreased from 1.97 to 1.62. After the policy shock, the age-standardized rate (ASR) of years lived with disability (YLD) in the Caribbean increased significantly to 4.59 (95% CI: 0.96 to 8.22), while the all-age YLD rate in the U.S. increased (5.18, 95% CI: 0.66 to 9.70) for both sexes.

**Conclusion:**

This study explores the impact of social factors and health campaigns on the burden of idiopathic epilepsy. We emphasize the need for targeted prevention and treatment strategies to effectively address the burden of idiopathic epilepsy.

## Introduction

Idiopathic epilepsy, one of the most common neurological disorders worldwide, is characterized by a high global prevalence and mortality rate (1). In 2021, there were a total of 24220856.23 cases of idiopathic epilepsy and 139850.93 idiopathic epilepsy-related deaths worldwide, particularly in low- and middle-income countries (2). Previous studies have systematically analyzed the trends in the burden of idiopathic epilepsy (2, 3). However, analyses of the impact of social factors, large-scale health campaigns, and policies on the burden of epilepsy are lacking. Idiopathic epilepsy is a highly prevalent neurological disorder that is closely associated with social factors and imposes a significant burden on the socioeconomic and healthcare systems. Patients with idiopathic epilepsy simultaneously face physical health and sociopsychological challenges (4). The cost of treating idiopathic epilepsy also places a heavy burden on families and national public health systems. To address the substantial burden of the disease, the World Health Organization (WHO), the International League Against Idiopathic Epilepsy (ILAE), and various national and regional health organizations have launched a series of initiatives. It is essential to gain a deeper understanding of the impact of these initiatives on the burden of disease.

Previous studies based on the GBD framework have focused primarily on the epidemiological trends and geographical distribution of epilepsy (2). These investigations provide systematically evaluated and relatively reliable primary data that form the foundation of our analysis. However, previous studies lack a comprehensive understanding of the burden of epilepsy from the perspective of health campaigns and policy interventions and often overlook the role that such initiatives play in shaping disease outcomes.

While a growing body of literature has examined the effects of health movements and policy actions, these studies commonly suffer from limitations. Some utilize disease burden data without employing robust or appropriate statistical models, leading to potentially biased or inconclusive findings (5). Others lack empirical evidence grounded in disease burden metrics, which limits their relevance and applicability for health policy evaluation. These gaps underscore the need for a more integrated and methodologically rigorous approach to assess the interplay between policy implementation and the burden of idiopathic epilepsy (6).

We designed the present study to address these deficiencies and contribute new insights to the field. We selected two large-scale international campaigns against idiopathic epilepsy as samples to assess the impact of transnational health policies on the burden of idiopathic epilepsy. The first campaign is “Out of the Shadows: A Global Campaign Against Epilepsy (GCAE),” which was launched in 1997, and the second is Revolution WHA 68.20. In November 2012, the Pan American Health Organization (PAHO), the ILAE, and the International Bureau for Epilepsy (IBE) jointly signed a memorandum of understanding and established a 10-year partnership to implement a strategic plan for epilepsy across the Americas. This initiative serves as an example of the WHO’s regional actions for epilepsy control. In the 2019 GBD study, the Latin American and Caribbean (LAC) region ranked third in terms of the global epilepsy burden. Therefore, we selected the Caribbean region as a model for regional policy and analyzed the impact of these policies on YLD due to idiopathic epilepsy. The U.S. epilepsy plan that began in 2012 was chosen as a case for national analysis. We used the disease burden of idiopathic epilepsy as the evaluation outcome. From an interdisciplinary perspective, we assessed the effects of these influential epilepsy-related health campaigns and policies using two advanced DID methods that are commonly applied in economics.

In this study, we explore the impact of social factors, including sociodemographic characteristics and the level of social development, on the burden of disease. We also assess the relationship between policies and idiopathic epilepsy, evaluate the impact of policy implementation on reducing the disease burden, and provide guidance for policy improvements. This study fills a gap in the relevant literature.

## Materials and methods

### Data source and disease definition

We utilized data on idiopathic epilepsy from the GBD 2021 results.^1^ The GBD 2021 study employed the latest epidemiological data and rigorous standardized methodologies to systematically and comprehensively assess health losses related to 371 diseases, injuries, and conditions as well as 88 risk factors across 204 countries and territories (7, 8). According to the WHO definition, epilepsy is a brain disease characterized by abnormal electrical activity that causes seizures, unusual behavior, sensations, and sometimes loss of awareness (9). In the GBD 2021, the ICD-10 codes for idiopathic epilepsy ranged from G40 to G41.9.

### Estimation framework

The GBD 2021 models the prevalence and incidence of most diseases and injuries using Bayesian meta-regression disease modeling meta-regression (DisMod-MR 2.1), with spatiotemporal Gaussian process regression (ST-GPR) replacing DisMod-MR 2.1 for certain diseases (7). DisMod-MR 2.1 is a Bayesian disease modeling meta-regression tool that generates estimates for prevalence, incidence, remission, and mortality rates by sex, location, year, and age group. ST-GPR is a regression method used to statistically smooth heterogeneous and incomplete data across time, age, and location. Moreover, the cause of death for most diseases and injuries is estimated using the cause of death ensemble model (7). The burden of idiopathic epilepsy is assessed by years lived with disability (YLD), whereas premature mortality is measured by years of life lost (YLL). The sum of YLD and YLL is used to calculate disability-adjusted life years (DALYs) (7).

### Sociodemographic index and human development index

The sociodemographic index (SDI) represents the geometric mean of three parameters for a given location: lag-distributed income per capita, average years of schooling, and the fertility rate in females younger than 25 years (7). The SDI ranges from 0 to 1 and reflects the level of socioeconomic development and its correlation with disease burden (10). The HDI, created by the United Nations, measures average achievements in key dimensions of human development, including a long and healthy life, knowledge, and a decent standard of living (11).

### Decomposition analysis

To further understand the driving factors of changes in the idiopathic epilepsy burden from 1990 to 2021, we applied the method detailed by Xie et al. (12) in a GBD 2016 study. This method quantifies the changes in four indicators of the idiopathic epilepsy burden over this period into three contributing factors: population, aging, and epidemiological changes. Taking DALYs as an example, the number of DALYs can be expressed by the following formula:


$$\mathrm{DAL}Y_{ay,py,ey}=\sum_{i=1}^{20}a_{i,y}\times p_{y}\times e_{i,y}$$

where DALY ay, py, and ey represents DALYs based on the factors of age structure, population, and DALY rates for a specific year y; $a_{i,y}$ represents the proportion of the population in age category i of the 20 age categories in a given year y; $p_{y}$ represents the total population in a given year y; and $e_{i,y}$ represents the DALY rate given age category i in year y. The contribution of each factor to the change in DALYs from 1990 to 2021 is defined by the effect of one factor changing while the other factors are held constant.

### Cross-country health inequality analysis

In the analysis of health inequality, we measured disparities in the burden of idiopathic epilepsy across countries using the slope index of inequality (SII) and the concentration index of health inequality (CIX) (13, 14). The SII was calculated by regressing national mortality rates and DALY rates for all-age populations on the relative position scale of the SDI. This scale is defined by the midpoint of cumulative population strata ranked by per capita gross domestic product. Heteroscedasticity was accounted for using a weighted regression model, and the log transformation of relative social position values was applied to address non-linearity due to marginal utility. After the populations were ranked by income and mortality/DALY disease burden, the CIX was calculated by fitting the Lorenz concentration curve and numerically integrating the area under the curve. Additionally, we calculated the SII derived from the health inequality analysis of the DALY disease burden from 1990 to 2021. We fitted a spline curve to observe the trend of changes in the SII.

### Generalized difference-in-differences

Difference-in-differences (DID) is a statistical scale used to evaluate the causal effects of policies, programs, and interventions based on the parallel trends assumption, in which the confounding factors that affect treatment effects before the intervention are equivalent to those that affect treatment effects after the intervention (15). For DID, during the post-treatment period t1, the average effect of treatment on the treated (ATT) is defined as


$$E\left[Y_{1}\left(i,t_{1}\right)-Y_{0}\left(i,t_{1}\right)A\left(i\right)=1\right]$$

where A(i) represents the treatment status, with A(i) = 1 if individual i is treated and A(i) = 0 otherwise. Y(i,t) is the outcome of interest for individual i at time t, with two observation periods: pretreatment (*t* = t0) and post-treatment (*t* = t1). We used a generalized difference-in-differences (GDID) model to analyze the causal effects of the global idiopathic epilepsy policy and other confounders on the burden of idiopathic epilepsy. For GDID, during the post-treatment period t1, the ATT is defined as


$$ATT_{\mathrm{GDID}}=E\left[Y\left(t_{1}\right)-Y\left(t_{0}\right)t\left(Z\right)=1\right]-E\left[Y\left(t_{1}\right)-Y\left(t_{0}\right)t\left(Z\right)=0\right]$$

Z represents a confounding factor defined as an instrumental variable. $t\left(Z\right)=E\left(AZ\right)$ and $E\left[Y\left(t_{1}\right)-Y\left(t_{0}\right)t\left(Z\right)\right]$ are both assumed to be linear in Z and t(Z), respectively. The details of the GDID and DID analyses are described in the Supplementary Methods section.

### Synthetic difference-in-differences

We use the synthetic difference-in-differences (SDID) method to estimate the causal impact of idiopathic epilepsy-related actions on idiopathic epilepsy YLD. SDID is an extension of the DID model that combines the advantages of DID and synthetic control methods to provide a more robust estimate of a treatment effect when the parallel trends assumption of traditional DID might be violated. Owing to the lack of a natural control group that closely resembled the treated units, identifying a suitable single comparison group was challenging. Therefore, we employed the SDID approach to conduct our analysis. SDID is used to construct a synthetic control group from the non-treatment group by data-driven weights and to ensure that the outcome of this synthetic control group is parallel to the pretreatment average outcome of the treatment group (16, 17). It is also used to balance the time weights. For SDID, ATT is defined as


$$\begin{array}{l} ATT_{\mathrm{sdid}}=E\left[Y,t>T_{0},D=1\right]-E\left[\hat{\lambda_{t}}Y,t\leq T_{0},D=1\right]\\ -\overset{N-K}{\underset{n=1}{\sum }}\hat{\omega_{n}^{\mathrm{sdid}}}\left(E\left[Y,t>T_{0},D=0\right]-E\left[\hat{\lambda_{t}}Y,t\leq T_{0},D=0\right]\right) \end{array}$$

Here, Y represents the outcome of interest, where $t>T_{0}$ indicates the posttreatment period and $t\leq T_{0}$ indicates the pretreatment period. D is a dummy variable that indicates whether the region is a demographic region. The detailed methodology of SDID is provided in the Supplementary Methods section.

### Interventions

#### Global campaign against epilepsy (GCAE)

In 1998, the first regional meeting of the GCAE was held in Heidelberg, Germany, where the first regional declaration was unanimously adopted. Since then, the disease burden of epilepsy has gained increasing attention, and multiple regional and global policies to control the disease burden have been implemented. We chose 1998 as the policy shock point, which is the specific time point at which a major health policy, intervention, or campaign is introduced or implemented.

#### Revolution WHA 68.20

Up-to-date evidence on the levels and trends of YLD is important for global, regional, and national health policies. We selected the most recent international policy on idiopathic epilepsy, Revolution WHA 68.20, and analyzed its impact on YLD. We chose 2016, 1 year after the revolution was first posted, as the policy shock point.

#### Movement of PAHO and the WHO in the Caribbean

In 2013, PAHO and the WHO released the *Report on Epilepsy in Latin America and the Caribbean* (18), which systematically compiled and analyzed key data on care resources, programs, and services for people with epilepsy in Latin American and Caribbean countries. The report enabled these countries to identify their strengths and weaknesses and prioritize areas to improve the response of the health sector. We consider this the official starting point of the movement in the Caribbean region. Considering that policy implementation takes time, we delayed the policy impact to 2014.

#### U.S. epilepsy plan

In addition to analyzing the impact of global and regional policy, we chose the U.S. epilepsy plan that began in 2012 as a case for national analysis. In 2012, the Institute of Medicine (IOM) released the groundbreaking report titled *Epilepsy Across the Spectrum: Promoting Health and Understanding* (19), which was followed by actions from Health and Human Services (HHS) in 2014 (20). We selected 2014 as the policy shock point.

### Statistical analyses

Spearman correlation was used to assess the correlation between the EAPCs in age-standardized DALY rates and the SDI and HDI. All data analyses and visualizations were performed using R software version 4.4.2 and Stata 16, with a two-sided *p*-value < 0.05 considered statistically significant. The R package DasGuptR was used for decomposition analysis, and the R package synthdid was used for SDID analysis.

## Results

### The role of the SDI and HDI

**Figure 1:**
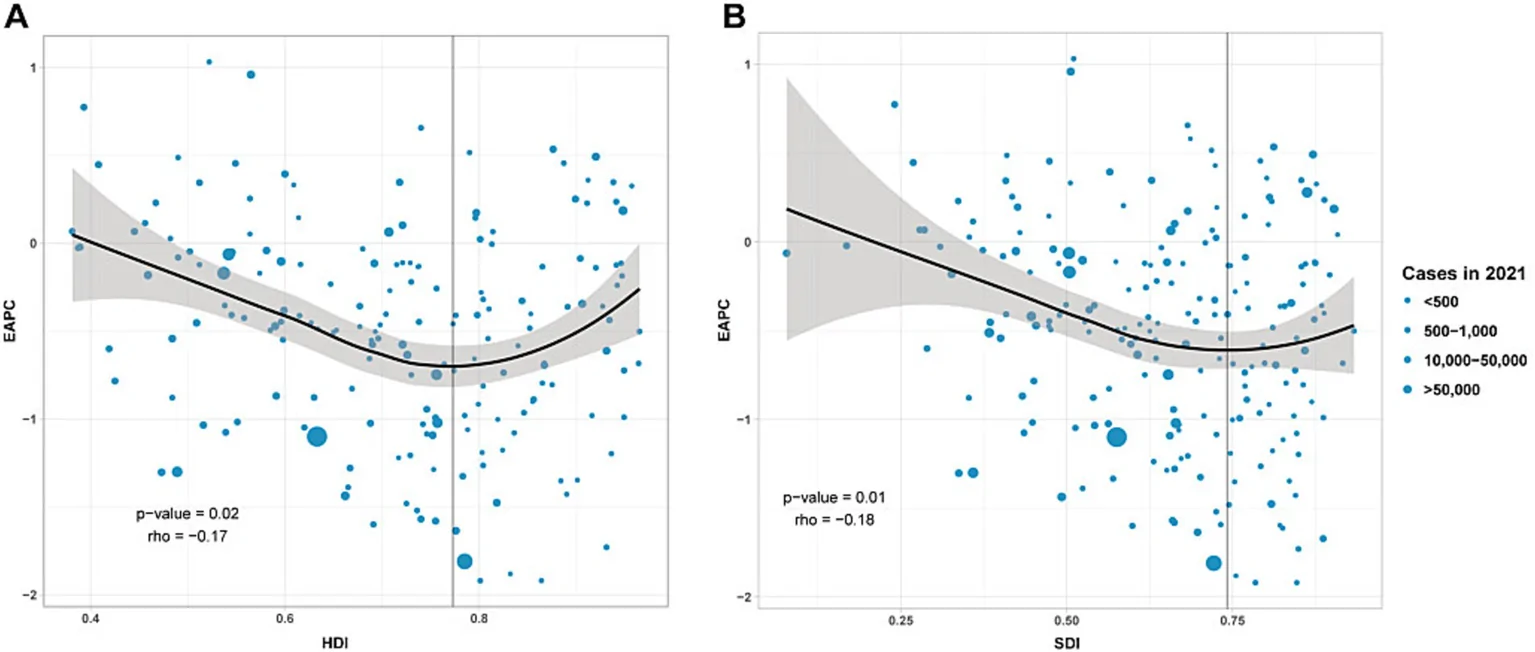
Correlations between the EAPC of the age-standardized DALY rate and **(A)** HDI or **(B)** SDI. Shading represents the 95% confidence interval.

**Figure 2:**
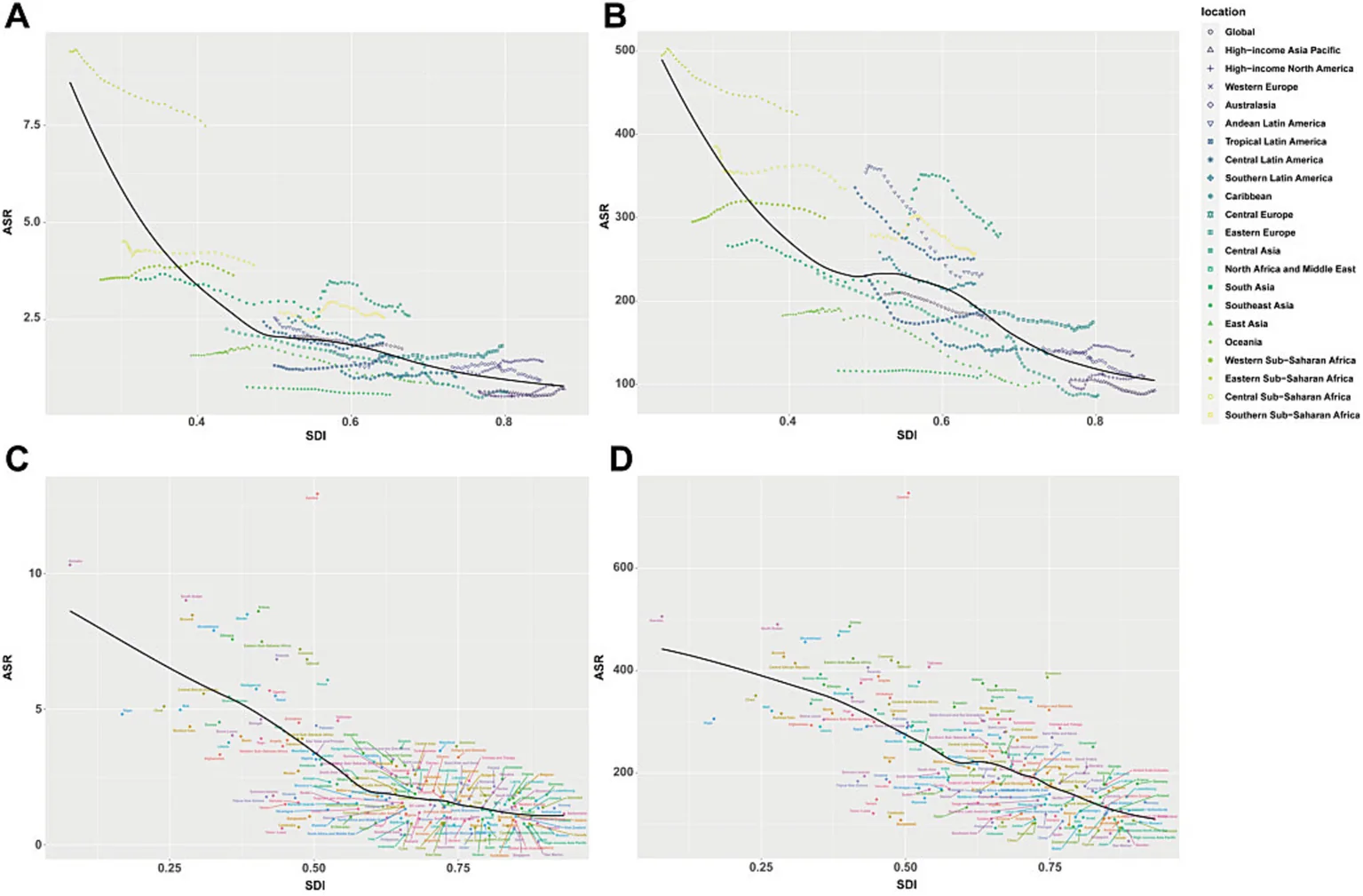
Age-standardized DALYs **(B,D)** and ASDR **(A,C)** for idiopathic epilepsy in 21 GBD regions by SDI from 1990 to 2021 and 204 GBD countries in 2021.

We conducted separate correlation analyses between the HDI and SDI and the EAPC in DALYs. The correlation analysis for the SDI yielded a rho value of −0.18, whereas the correlation analysis for the HDI yielded a rho value of −0.17 (Figure 1). Both results were statistically significant. As the HDI and SDI increase, the EAPC initially decreases but then increases. Additionally, we observed that the ASDR and age-standardized DALY rate in GBD super regions decreased with increasing SDI (Figure 2). This finding is consistent with the trend we fitted using data from each country in 2021 (Figure 2).

### Decomposition analysis of changes in idiopathic epilepsy

**Figure 3:**
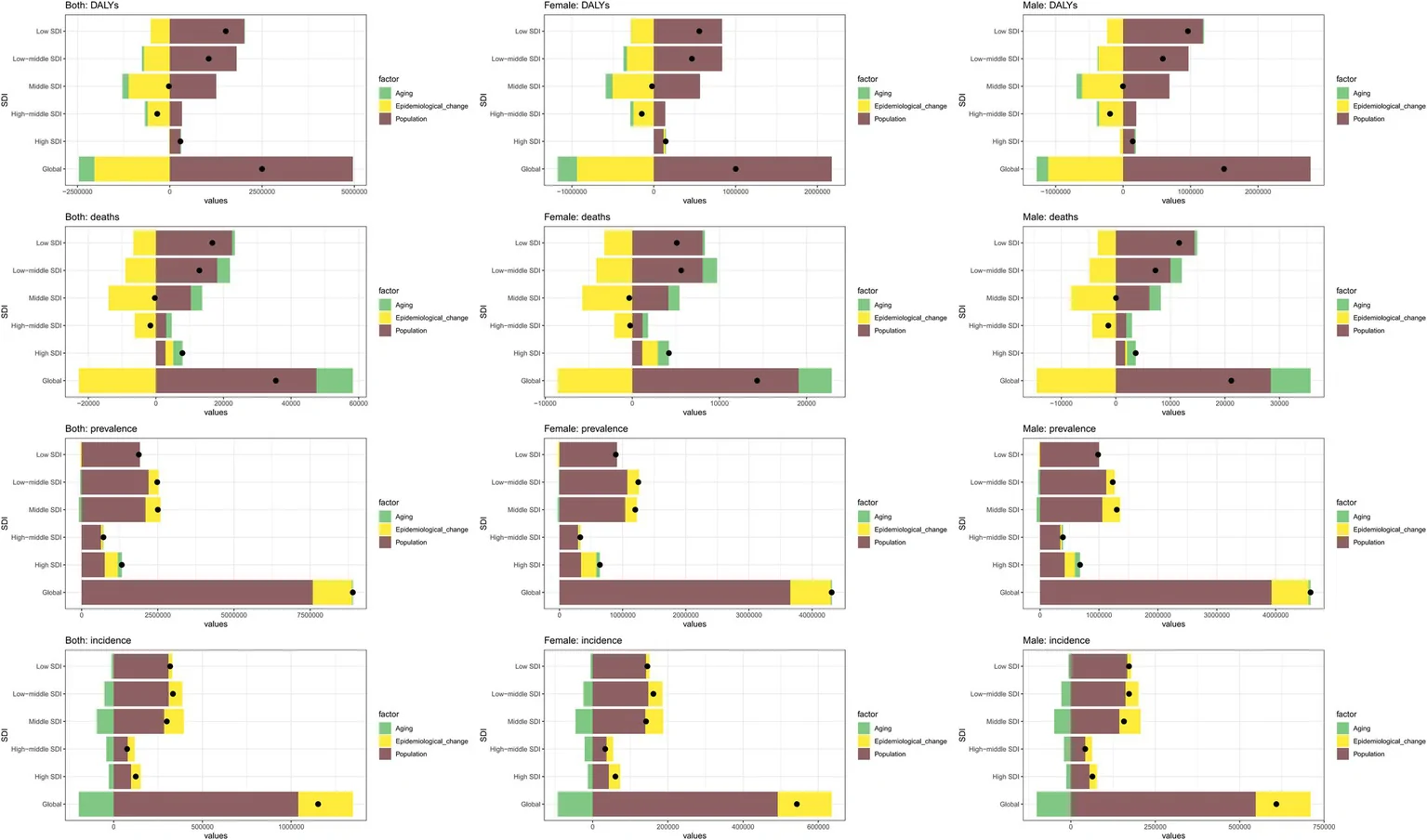
Changes in DALYs associated with idiopathic epilepsy according to three population-level determinants: age, population and epidemiological changes at the global and regional levels. The black dots indicate the total value of change attributable to all three components.

We conducted a decomposition analysis for regions at the global level and five SDI levels and attributed the disease burden of idiopathic epilepsy to three population-level determinants (aging, population, and epidemiological changes). The results of the DALY analysis revealed that high-SDI regions were least affected by epidemiological changes (−6.49%), whereas middle-SDI regions were most affected (4650.94%). The regions that were least affected by aging were the low-SDI regions (0.90%), while the middle-SDI regions were most affected (696.32%). The high-SDI regions were least affected by population (98.84%), while the middle-SDI regions were most affected (−5247.26%) (Figure 3 and Supplementary Table S1). For all SDI regions except the low-middle and high-middle regions, the impact of population on DALYs was positive, whereas the impact of epidemiological changes was negative (Figure 3 and Supplementary Table S1). The results for males are similar to those for both sexes. However, there are some differences between the results for males and females. In addition to changes in the proportions of the three influencing factors, the differences are reflected mainly in the following aspects: compared with the results for males, in low-SDI regions, the impact of aging shifted from positive to negative for females, while in high-SDI regions, the impact of epidemiological changes shifted from negative to positive for females (Figure 3 and Supplementary Table S1).

With respect to incidence in all SDI regions, regardless of sex, aging has a negative impact, whereas epidemiological changes and population have positive impacts. In terms of prevalence, population is the dominant factor (Figure 3 and Supplementary Tables S2–S4).

### Cross-country health inequality analysis

**Figure 4:**
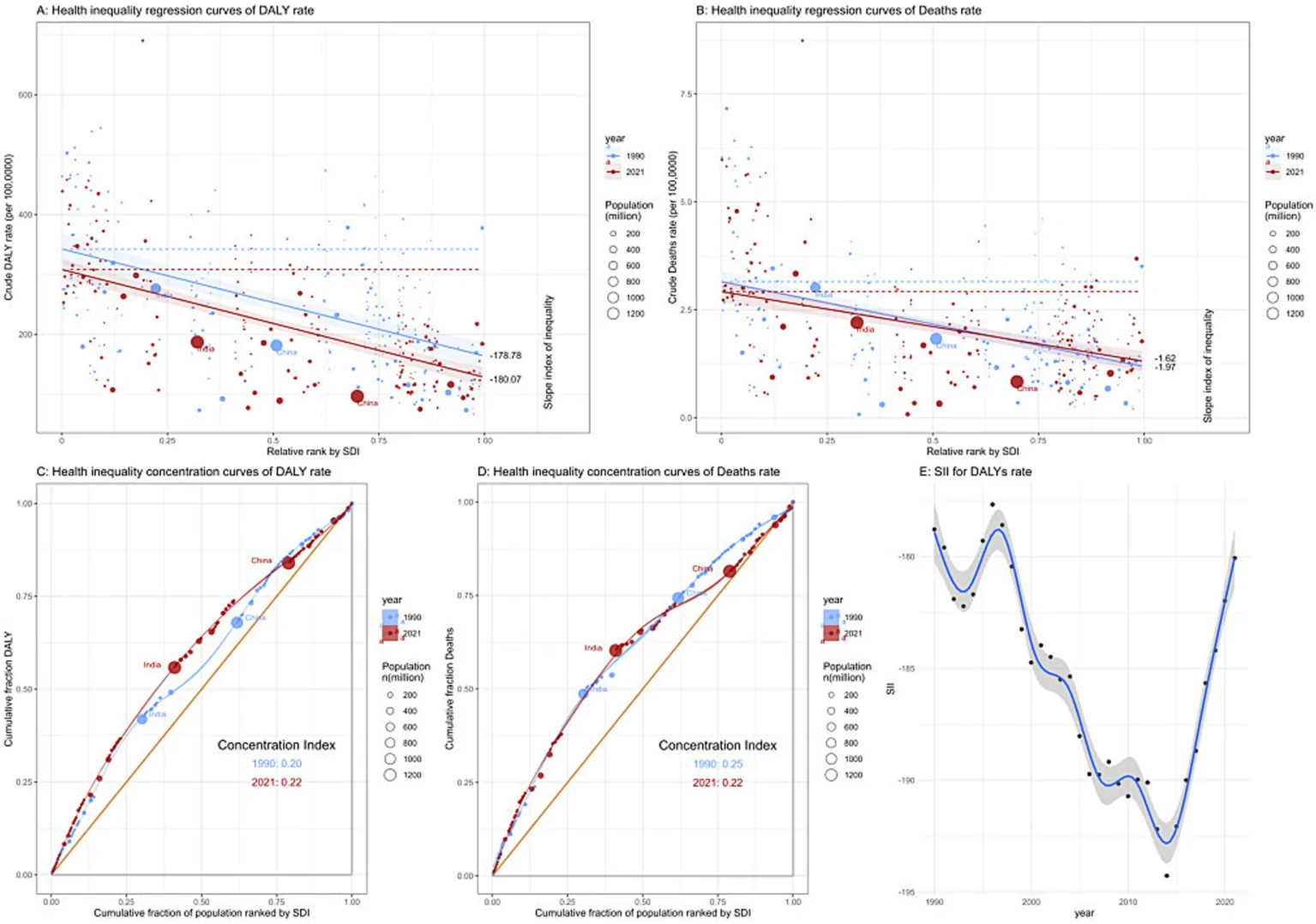
Health inequality regression curves and concentration curves for DALYs **(A,C)**, mortality **(B,D)** and the SII for the DALY rate of idiopathic epilepsy **(E)**. Shading represents the 95% confidence interval.

We found mortality and DALY inequalities across countries. From 1990 to 2021, the concentration index of DALYs increased slightly and the slope index of inequality also rose, indicating a slight increase in health inequalities between countries with high and low SDIs (Figures 4A,C). The slope index of inequality for the mortality burden decreased from 1.97 to 1.62, and the concentration index decreased from 0.25 to 0.22 (Figures 4B,D). These increases and decreases were relatively modest, suggesting that there was no evident change in the DALYs or mortality burden.

By examining the SII curve fitted with the spline function, we found that the health inequality index of the DALY disease burden showed a clear trend of initially decreasing and then increasing from 1990 to 2021 (Figure 4E). This indicates that global health inequality related to DALYs worsened rapidly from 1990 to 2014 but has improved continuously since 2014.

### The impact of policies and campaigns on the disease burden globally

The results revealed that the all-age DALY rate for both sexes had a downward trend after the policy was implemented with a delayed effect (Supplementary Figure S1). This is understandable as the promotion of global policies requires time. Our analysis by sex showed that females had a very strong pre-policy trend, which refers to the trajectory or pattern of a health outcome over time before the implementation of a specific policy or intervention. The DALY rates for males were not significant before the policy but showed a subsequent rapid downward trend, reflecting a strong impact of the policy on males (Supplementary Figure S1).

Analysis of the all-age incidence rate revealed a significant downward trend after the implementation of the policy with a delay over a certain period. The results for males showed a downward trend after the policy shock with a considerable delay, suggesting that the policy’s impact on the incidence in males may be slow and limited (Supplementary Figure S2). The decline in results for females after the shock was pronounced, suggesting that the policy’s impact on female incidence may be rapid and strong. This sex difference may be influenced by factors such as the life expectancy gap between males and females, lifestyle habits, and physical structure.

**Figure 5:**
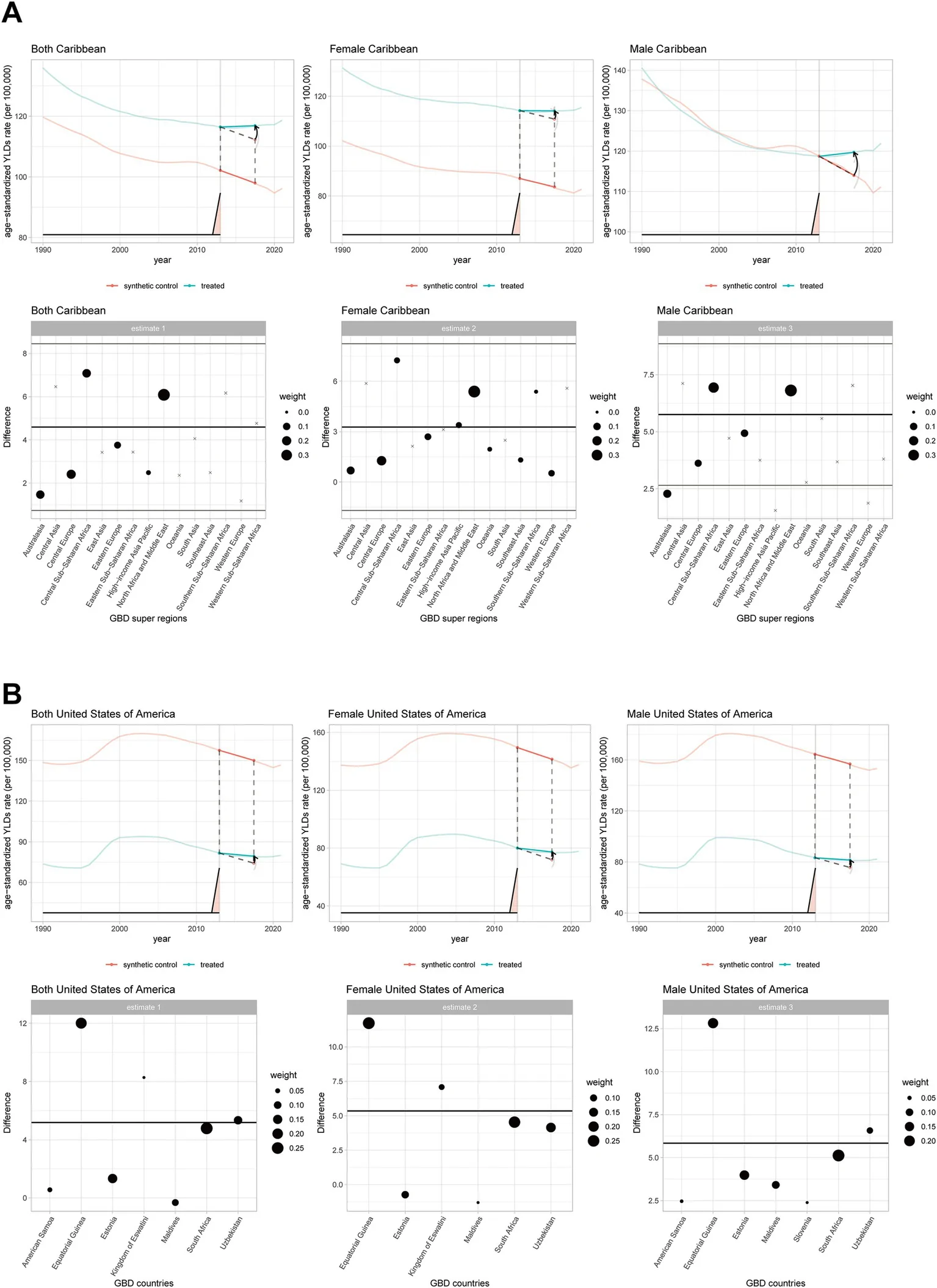
Effects of campaigns led by the PAHO/WHO, ILAE and IBE on idiopathic epilepsy YID rates in the Caribbean region and America. Panels **(A,B)** present the effects of the campaign by gender. Estimates 1–3 of **(A,B)** present the weights of the synthetic control line.

When analyzing the all-age mortality rate, we find a delay after the policy shock and an upward trend after the initial decline. This trend was more pronounced in the male population, suggesting that the policy had a short-term effect and that its efficacy decreased over time. The results for females revealed a pre-policy trend with a subsequent decline, indicating the sustained impact of the policy (Supplementary Figure S3). The results for the all-age YLL rates for both sexes were similar to those for the all-age mortality rates (Figure 5).

**Figure 6:**
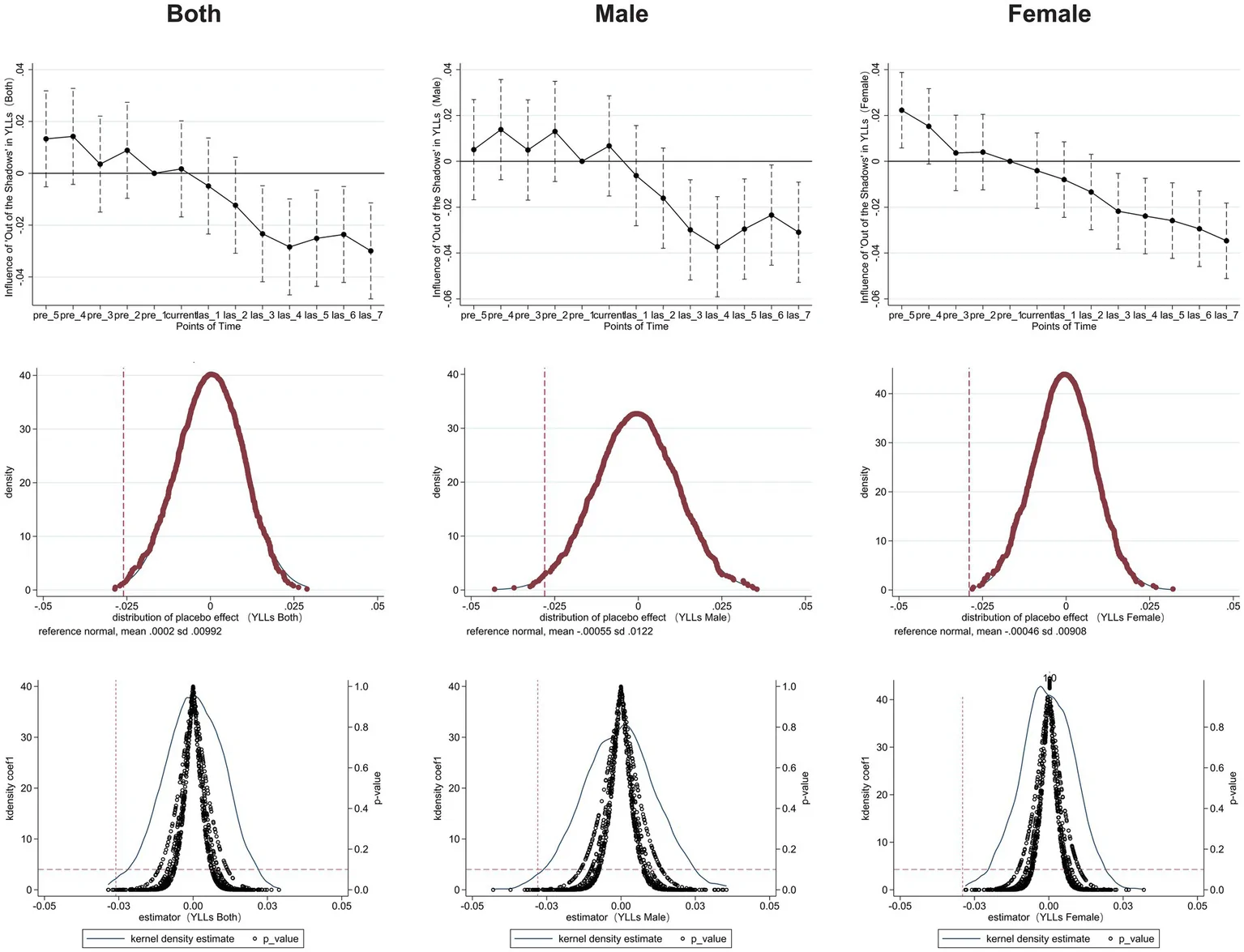
Effect of “out of the shadows: a global campaign against epilepsy” on global idiopathic epilepsy YLL rates and placebo tests. Current refers to the shock point. Dashed lines represent the 95% confidence intervals. The red vertical dashed line represents the true coefficient, and the red horizontal dashed line represents a *p*-value of 0.1.

To verify the reliability of the results, we conducted placebo tests and benchmark regressions. All benchmark regressions and placebo tests for the analyses were statistically significant, except for the incidence of males (Figure 6; Supplementary Figures S1–S4; Supplementary Table S5).

### The impact of policies and campaigns on YLD globally, regionally, and nationally

The results of Revolution WHA 68.20 show that after the policy shock, the all-age YLD rate exhibited a significant upward trend (Supplementary Figure S4).

After the policy shock in the Caribbean, the ASR of YLD significantly increased to 4.59 (95% CI: 0.96 to 8.22). The differences between genders were striking: the result for males was 5.76 (95% CI: 2.78 to 8.73), which was much greater than the 3.27 found for females (95% CI: −1.61 to 8.15) (Figure 6). The policy generated a noticeable gender difference, which may be related to the characteristics of the disease burden and social conditions in the Caribbean region.

For the U.S. epilepsy plan, the results for both sexes (5.18, 95% CI: 0.66 to 9.70) indicate that the policy led to a significant increase in the all-age YLD rate in the U.S. Specifically, the result for females was 5.34 (95% CI: 0.73 to 9.96), and the result for males was 5.84 (95% CI: 0.69 to 10.99) (Figure 6). We observed that the gender difference caused by U.S. policies was much smaller than the gender difference in the Caribbean region.

## Discussion

### Main findings

The burden of idiopathic epilepsy is strongly correlated with both the SDI and the HDI, suggesting that national levels of social and economic development may play a critical role in shaping disease outcomes and healthcare disparities. When the HDI and SDI increase, the EAPC of DALYs initially decreases but subsequently increases. After decomposition analysis, aging was found to have a negative effect on the incidence of idiopathic epilepsy, indicating that age-related factors may be associated with a decline in new cases over time. In contrast, population growth emerged as the predominant driver of prevalence, suggesting that demographic expansion continues to exert substantial pressure on the overall disease burden. These findings highlight the importance of tailoring prevention and resource allocation strategies according to the distinct demographic determinants of incidence and prevalence. The slope index of inequality for the mortality burden decreased from 1.97 to 1.62, indicating a modest reduction in global health disparities over time.

Our findings highlight the nuanced and often delayed effects of global, regional, and national epilepsy policies on the burden of idiopathic epilepsy. The initial implementation of the GCAE in 1998 corresponded with overall declines in DALYs and incidence rates; however, the observable impact was not immediate, underscoring the inherent lag in translating policy into population-level outcomes. Notably, gender disparities were evident across multiple indicators: females responded more rapidly and consistently to policy interventions in terms of incidence reduction, whereas males exhibited delayed but significant changes, particularly in DALYs and YLDs. These sex-specific responses may reflect differences in biology, behavior, and healthcare access and merit targeted approaches in future health strategies. The significant increase in YLDs following recent global and regional policy efforts, including WHA Resolution 68.20 and PAHO/WHO initiatives, may reflect improved diagnosis and reporting, improved service accessibility, or a real increase in the non-fatal disease burden. Particularly in the Caribbean, the gender gap in YLD outcomes suggests that male patients may have experienced disproportionate changes in disease recognition or service provision. In contrast, the U.S. policy response led to more balanced increases in YLD between the sexes, which is potentially indicative of more equitable healthcare systems and targeted awareness campaigns.

### The role of the social development index

With the increase in both the SDI and the HDI, the EAPCs in DALYs due to idiopathic epilepsy generally exhibited a downward trend. However, beyond a certain SDI threshold, the EAPC demonstrated a slight upward inflection, an observation that deviates from conventional expectations. This counterintuitive trend may be attributable to the well-established healthcare systems and comprehensive epilepsy management frameworks in high-SDI and high-HDI countries. These countries often allocate substantial financial and institutional resources to health policy implementation to ensure adequate infrastructure for the prevention, diagnosis, treatment, and long-term care of epilepsy.

Conversely, positive EAPC values in low-SDI and low-HDI countries may reflect systemic challenges, including limited access to medical care among low-income populations, an inequitable distribution of public health services, an insufficient supply of antiepileptic drugs, a shortage of neurologists involved in epilepsy care, and the pervasive impact of stigma (21). These structural barriers likely contribute to the persistence or worsening of the disease burden over time.

To further elucidate the global health inequities, we quantified disparities between high-SDI countries and low-SDI countries and found that the extent of inequality in 2021 remained largely unchanged compared with that in 1990. The spline curve of the SII for DALYs due to idiopathic epilepsy revealed a U-shaped pattern that initially declined and subsequently increased in recent years. This rebound may be partly influenced by increasing geopolitical tensions and instability in international relations, which can adversely affect global health aid programs designed to reduce inequality (22). Health diplomacy and international cooperation are directly shaped by such geopolitical dynamics, and their disruption may be reflected in worsening health outcomes across vulnerable regions (23).

In addition, geopolitical conflict often undermines economic and cultural exchanges between countries, both of which play critical roles in shaping health systems and disease burdens. As previously discussed, economic downturns may lead to reduced funding for essential healthcare services, whereas a decrease in cross-cultural collaboration may limit the dissemination of evidence-based practices, thereby exacerbating the burden of idiopathic epilepsy in certain populations.

### Gender disparities in policy impact

Our SDID analysis revealed that the impact of identical policy interventions varied significantly by sex, with the most pronounced disparity in the age-standardized YLD rate for idiopathic epilepsy in the Caribbean. In contrast, no such disparity was evident in the United States. Given the relatively small gender gap in idiopathic epilepsy-related YLD ASR between the Caribbean and the U.S., we hypothesize that these divergent outcomes are not driven by biological or epidemiological differences but rather by differences in policy implementation and the broader sociocultural environment. Social norms, cultural expectations, and institutional structures significantly shape the way health policies are executed and resources are allocated, particularly in relation to gender (24). Discrimination and gender-based inequalities may therefore attenuate or amplify the effectiveness of policy interventions.

The application of a gender-sensitive lens is essential when evaluating the differential impact of health policies. In the Caribbean, persistent structural inequalities exist not only between socioeconomic strata (vertical inequality) but also between men and women (horizontal inequality). In fact, the Caribbean has some of the highest global rates of gender-based structural disparity. Compared with the United States, which is characterized by a predominantly individualistic culture, the Latin America and Caribbean (LAC) region has a more collectivist cultural framework. While this collectivism fosters social cohesion, it often reinforces patriarchal norms that restrict women’s autonomy and limit equitable access to rights and healthcare resources (25).

These systemic inequalities are quantitatively captured by the gender inequality index (GII) developed by the United Nations Development Programme (UNDP). The GII assesses gender gaps across three key domains: reproductive health (measured by maternal mortality and adolescent birth rates), empowerment (measured by the proportion of parliamentary seats held by women and educational attainment), and labor market participation. Even among the most economically developed countries in the LAC region, such as Argentina, Uruguay, and Chile, the GII remains higher (i.e., worse) than in the United States, indicating more pronounced gender disparities (26).

This broader sociopolitical context helps to explain why the Caribbean exhibited a significant sex-based differential in policy impact while the U.S. did not. Cultural norms, policy governance structures, and institutional gender sensitivity all play vital roles in shaping how interventions translate into population-level health outcomes. Future policy design and evaluation must explicitly incorporate gender equity frameworks to ensure balanced and effective health outcomes for both women and men.

### The role of YLD and epilepsy-related actions

Demographic, epidemiological, and gerontological studies have involved considerable debate on whether the prevalence of disability increases or decreases with epidemiological transition. Fries et al. suggested that as life expectancy increases and lifestyles change, the onset of chronic diseases is delayed, the age at first onset increases, the number of years spent with disease decreases, and YLD decreases (27). Olshansky et al. (28) proposed a different view: they emphasized that a decrease in mortality leads to an increase in YLD and highlighted the role of medical interventions in extending the lifespan of people with disabling diseases, thereby increasing YLD. A GBD 2010 study emphasized the importance of stable age-specific YLD per person, which increases steadily with age (29). As life expectancy increases, people may spend more years in poorer health, thus increasing YLD, because the added years are at older ages when disability rates are higher. These findings are inconsistent with Fries’ theory and suggest that as time progresses, there may be less evidence of the causes of YLD such that a decline may not be observed.

We found that under policy interventions, the YLD-related indicators of global, regional, and national results increased rapidly after the implementation of policies. This sharp increase in the short term may be due to the significant impact of epilepsy healthcare policies, which have increased the number of years lived with idiopathic epilepsy for already diagnosed patients. Globally, there was a preexisting trend before the policy interventions that may have been influenced by the global increase in life expectancy and some regional policies. Both the Caribbean and the U.S. showed a decreasing trend in the age-standardized YLD rate (ASYR) before the policy intervention, whereas the U.S. experienced little change in life expectancy and the Caribbean experienced a rapid increase in life expectancy before 2014. We observed an increasing trend in the U.S. idiopathic epilepsy ASDR (EAPC: 1.41, 95% CI: 0.92 to 1.90) (Supplementary Figure S6). The reduction in the U.S. ASYR may be explained by the change in the ASDR, according to S. Jay Olshansky. In contrast, the Caribbean showed an upward trend in the idiopathic epilepsy ASDR (EAPC: −0.76, 95% CI: −0.94 to −0.58) (Supplementary Figure S6). According to Fries (27), this may suggest that the rapid increase in life expectancy and healthier lifestyles in the Caribbean reduce the number of years spent with disease, thereby lowering YLD and masking the effect of a decrease in ASDR. After the policy intervention, due to strengthened medical interventions and various social measures related to epilepsy care, patients in the Caribbean and the U.S. experienced an increase in years lived with idiopathic epilepsy, which caused YLD to rise. This increase reflects the effectiveness of the related policies.

### Implications for public health and research

On the basis of our findings, we encourage targeted global public health actions. A significant proportion of children and adolescents develop drug-resistant epilepsy, which leads to higher mortality rates and poorer cognition (30). This situation calls for the development of more suitable antiepileptic drugs and treatments for children. Additionally, some caregivers may find it difficult to communicate with medical teams and other important figures about their child’s seizures (31), which highlights the need for stronger cooperation among patients, families, communities, and care teams. The school system can play an important role through close collaboration with school nurses to create a non-discriminatory environment and the spread of epilepsy-related care knowledge. Moreover, we cannot ignore the impact of pediatric epilepsy on families and caregivers (31, 32). It is essential to provide more social support to families of children with epilepsy, including medical benefits and financial assistance, to alleviate their economic burden. Both caregivers and patients face stigma and psychological pressure, with caregivers experiencing greater worry, guilt, and feelings of failure. Providing mental health services for both patients and caregivers is strongly recommended.

For middle-aged and older adult individuals, we note the impact of underlying diseases such as cardiovascular diseases and hypertension on the onset of epilepsy (33). Older adults with these conditions should receive more attention, and additional treatment strategies for epilepsy comorbidity should be developed. Older adult individuals with epilepsy are more likely to experience cognitive dysfunction (34), which increases the complexity of care. Older adults are more likely to benefit from antiepileptic drugs for controlling seizures, but they are also more prone to potential side effects and require careful monitoring. With advances in healthcare and population aging, the number of older adult people with epilepsy will significantly increase globally. Early diagnosis and timely, appropriate individual management can prevent unnecessary hospitalization and reduce the impact of comorbidities. Similarly, closer integration between primary and secondary healthcare will improve the care of older adult patients with epilepsy (33). More funding should be allocated to the epilepsy care system, and more specialized epilepsy doctors should be trained. Additionally, the establishment of a well-connected medical system should be promoted. The development and promotion of early diagnosis techniques for older adult patients with epilepsy will save subsequent medical resources and reduce the medical burden.

We emphasize the impact and role of policies on the burden of epilepsy and encourage sustained and stable cross-departmental and cross-national policies. Our results show that past policies and actions had positive effects, but their duration was limited. For example, the GCAE project in China is expanding rural epilepsy control programs, but other GCAE activities have gradually weakened or disappeared (35). We also encourage policies focused on eliminating the disparity in medical resources for people with epilepsy between rural and urban areas. People with epilepsy who live in rural areas tend to have worse health outcomes than those in who live in urban areas, a phenomenon that is particularly evident in both high-income and low-income regions (36, 37). More epilepsy-related medical resources and financial support should be invested in rural areas to reduce this regional disparity. In addition, there are significant differences in the disease burden between countries that remained almost unchanged from 1990 to 2021. We call for support for countries and regions with high burdens of idiopathic epilepsy, such as Zambia and the Caribbean, to reduce the significant health inequalities that persist. We must also pay attention to epilepsy-related psychological issues and stigma, as relevant policies have been shown to effectively improve the quality of life for people with idiopathic epilepsy. Additionally, alcohol consumption is an important risk factor for idiopathic epilepsy. Alcohol control policies, such as raising alcohol taxes, banning underage drinking, and restricting the availability of alcohol, may help reduce the burden of idiopathic epilepsy.

### Strengths and limitations

This study is the first to use GBD data and DID-derived methods to assess the effects of epilepsy-related actions and policies, which allowed for a clearer understanding of their impact and effectiveness. Two advanced DID methods were applied to evaluate the impact of epilepsy-related actions and policies on the disease burden. These findings provide valuable insights and recommendations for future epilepsy-related actions and policy development. Additionally, we used various classical epidemiological theories to explain the impact of policy shocks on YLD, which offers a deeper understanding.

However, this study has several limitations. The quality of data across regions varies significantly, and some countries and regions have limited high-quality data. Furthermore, some low- and middle-income countries may lack reliable epidemiological data and experience case underreporting, which increases the uncertainty of the results. Additionally, the inclusion of healthcare claims data in the GBD data may introduce systematic bias, as individuals who did not receive treatment or were excluded from health insurance are not accounted for. Moreover, this study used only GBD data for the health outcome analysis, so the data sources are relatively narrow and one-sided.

## Conclusion

This study provides a comprehensive analysis of social factors and the effects of campaigns or public policies on the burden of idiopathic epilepsy. It offers a comprehensive evaluation of three principal determinants underlying the shifting burden of idiopathic epilepsy and highlights pronounced health disparities among countries at different stages of socioeconomic development. We systematically assessed the influence of representative, large-scale health interventions and policies from a global perspective as well as within the contexts of both underdeveloped and developed regions. Importantly, while these interventions demonstrated a substantial impact, notable sex-based disparities were concurrently observed across several epidemiological indicators. These findings not only serve as a data-informed retrospective on previous public health efforts but also offer critical insights to guide the development and optimization of future health policies. Reducing the burden of idiopathic epilepsy depends on accessible and widespread treatment and medication availability. We call for more lasting actions and policies for patients with epilepsy and encourage greater inclusivity and understanding toward people with epilepsy.

## Data Availability

Publicly available datasets were analyzed in this study. This data can be found: https://ghdx.healthdata.org/gbd-2021.

## Footnotes

^1^ https://vizhub.healthdata.org/gbd-results/

## Supplementary material

SUPPLEMENTARY FIGURE S1 Effect of “out of the shadows: a global campaign against epilepsy” on global idiopathic epilepsy DALY rates and placebo tests. Current refers to the shock point. Dashed lines represent the 95% confidence intervals. The red vertical dashed line represents the true coefficient, and the red horizontal dashed line represents a *p*-value of 0.1.

SUPPLEMENTARY FIGURE S2 Effect of “out of the shadows: a global campaign against epilepsy” on global idiopathic epilepsy incidence rates and placebo tests. Current refers to the shock point. Dashed lines represent the 95% confidence intervals. The red vertical dashed line represents the true coefficient, and the red horizontal dashed line represents a *p*-value of 0.1.

SUPPLEMENTARY FIGURE S3 Effects of “out of the shadows: a global campaign against epilepsy” on global idiopathic epilepsy mortality rates and placebo tests. Current refers to the shock point. Dashed lines represent the 95% confidence intervals. The red vertical dashed line represents the true coefficient, and the red horizontal dashed line represents a *p*-value of 0.1.

SUPPLEMENTARY FIGURE S4 Effect of revolution WHA 68.20 on global idiopathic epilepsy DALY rates and placebo tests. Current refers to the shock point. Dashed lines represent the 95% confidence intervals. The red vertical dashed line represents the true coefficient, and the red horizontal dashed line represents a *p*-value of 0.1.
