# Correction: Impact of social factors and health campaigns on the burden of idiopathic epilepsy: an inequality, decomposition, generalized and synthetic difference-in-differences study

**DOI:** 10.3389/fpubh.2026.1964398

**Published:** 2026-08-25

**Authors:** Xuewen Rong, Dongting Yu, Wei Zhao, Jingyu Xiao, Du Feng, Gengfeng Chen, Zemeng Cao, Liming Shu

**Affiliations:** 1 Department of Neurology, Institute of Neuroscience, Key Laboratory of Neurogenetics and Channelopathies of Guangdong Province and the Ministry of Education of China, The Second Affiliated Hospital, Guangzhou Medical University, Guangzhou, China; 2 Nanshan School, The Second Affiliated Hospital, Guangzhou Medical University, Guangzhou, China; 3 School of Pediatrics, Guangzhou Medical University, Guangzhou, China

**Keywords:** idiopathic epilepsy, disability-adjusted life years, generalized difference-in-differences, synthetic difference-in-differences, inequality, global burden of disease

Affiliation “Department of Neurology, Institute of Neuroscience, Key Laboratory of Neurogenetics and Channelopathies of Guangdong Province and the Ministry of Education of China, The Second Affiliated Hospital, Guangzhou Medical University, Guangzhou, China” was omitted for author Xuewen Rong. This affiliation has now been added for author Xuewen Rong.

Author Xuewen Rong was erroneously assigned to affiliation “Nanshan School, The Second Affiliated Hospital, Guangzhou Medical University, Guangzhou, China”. This affiliation has now been removed for author Xuewen Rong.

The original version of this article has been updated.

