# Supplementary figures and images for: Impact of social factors and health campaigns on the burden of idiopathic epilepsy: an inequality, decomposition, generalized and synthetic difference-in-differences study
Source: Front Public Health. 2025 Jun 2;13:1598497. doi: 10.3389/fpubh.2025.1598497 (PMC12171163; doi:10.3389/fpubh.2025.1598497)

Both

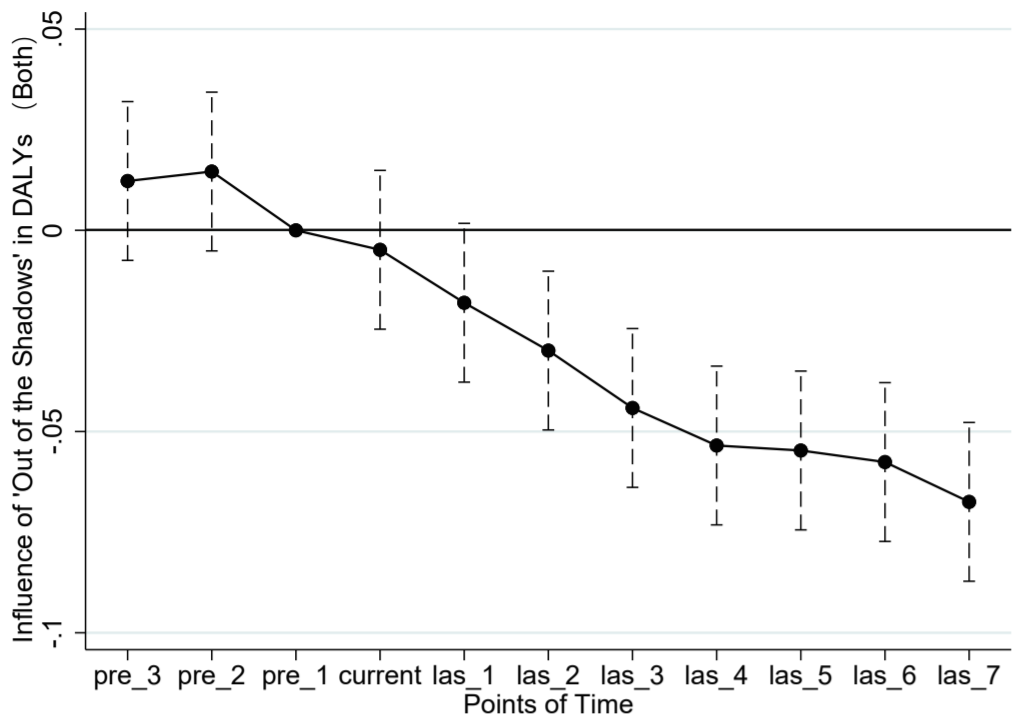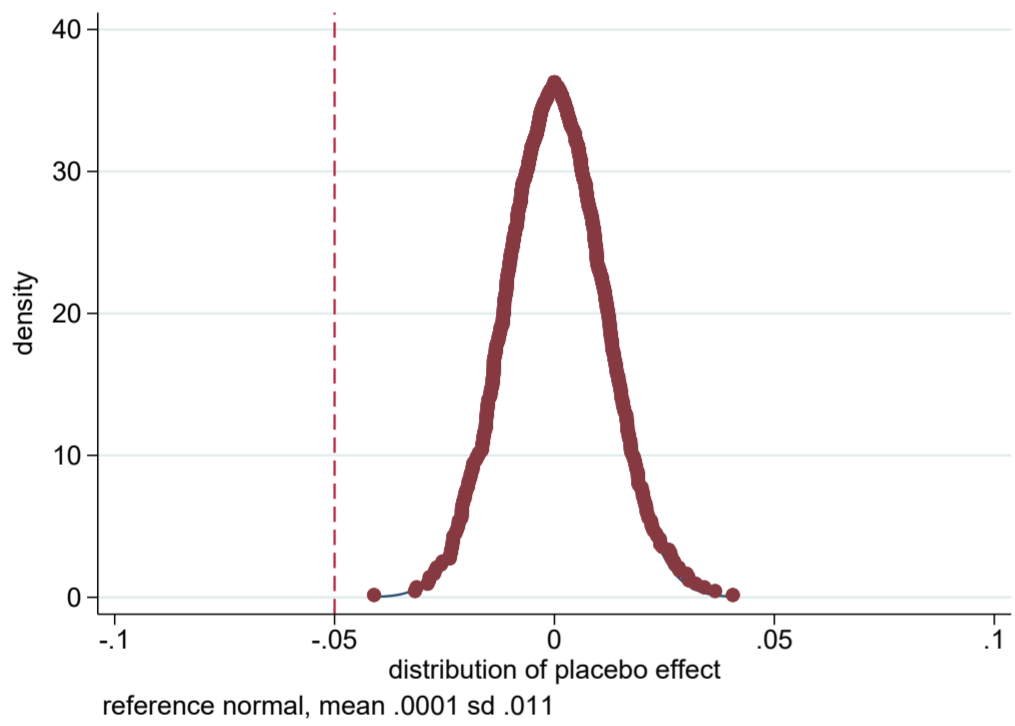

Male

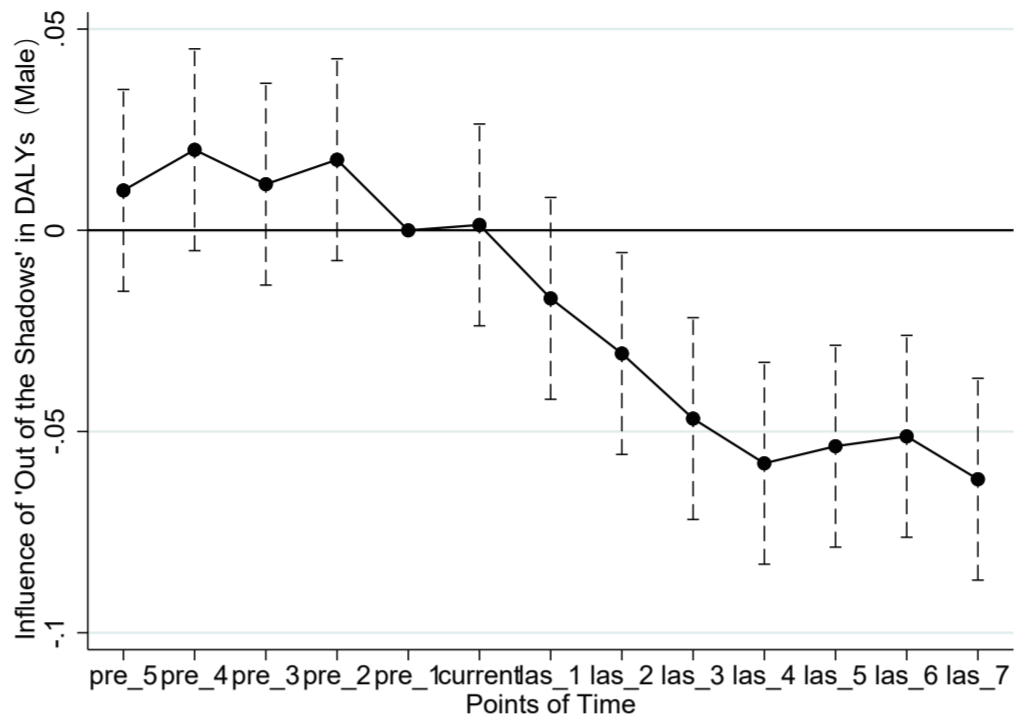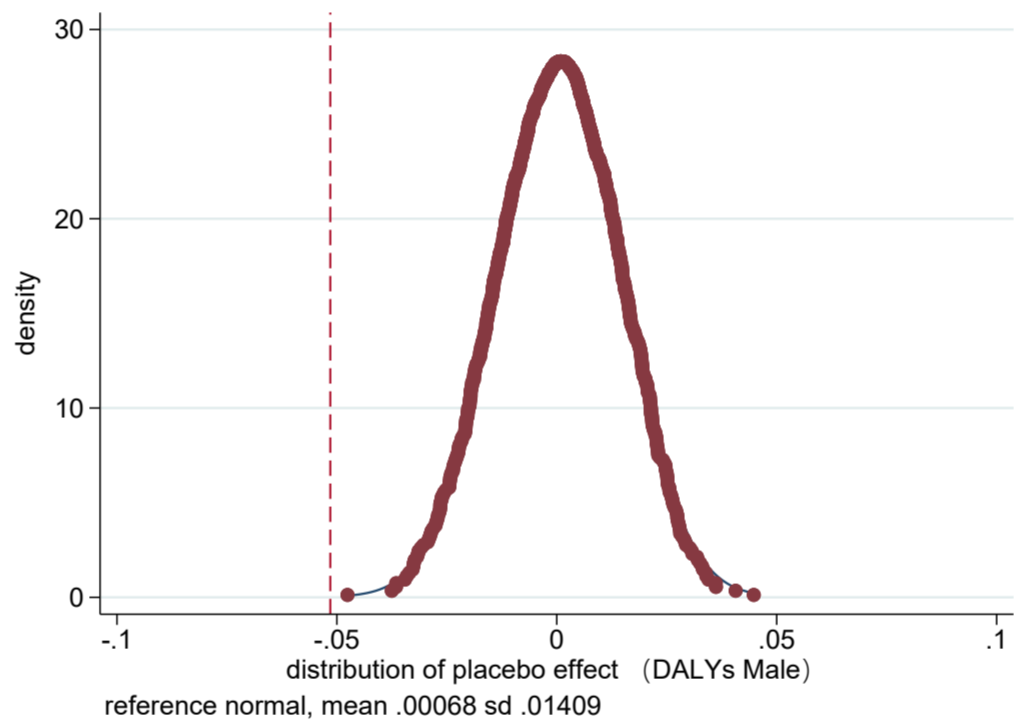

Female

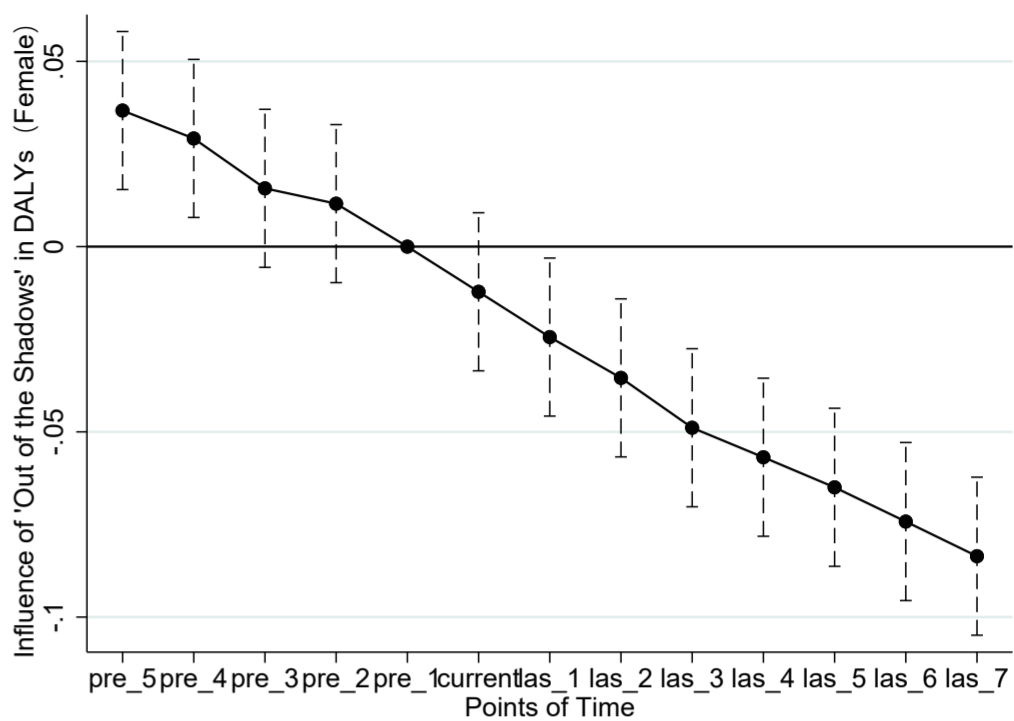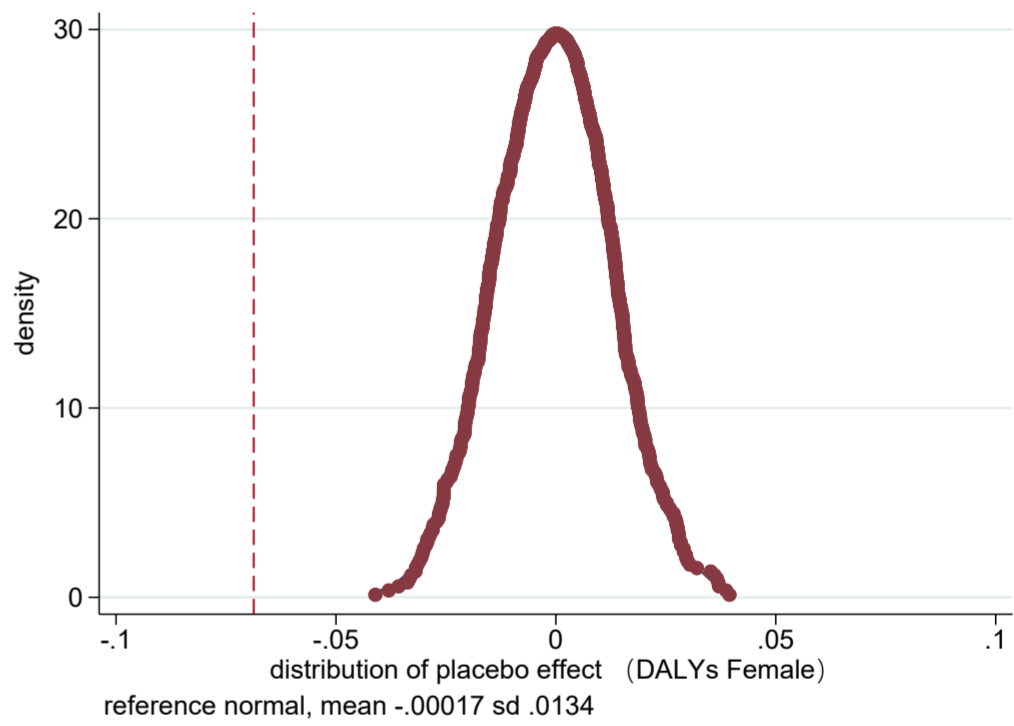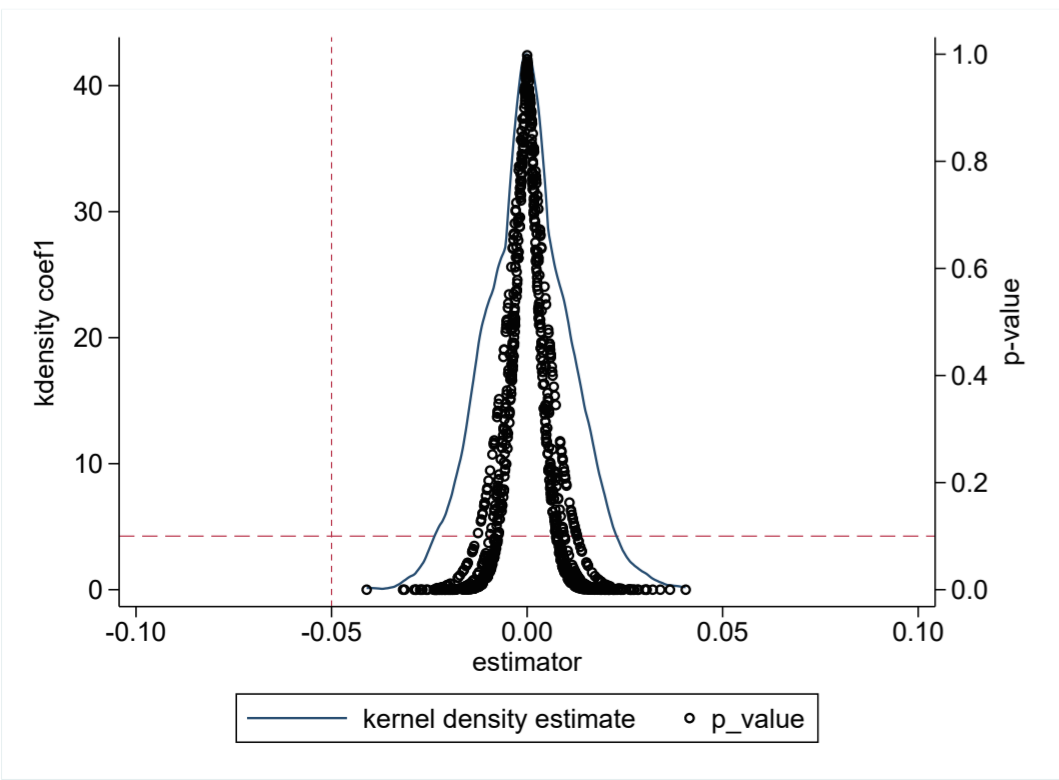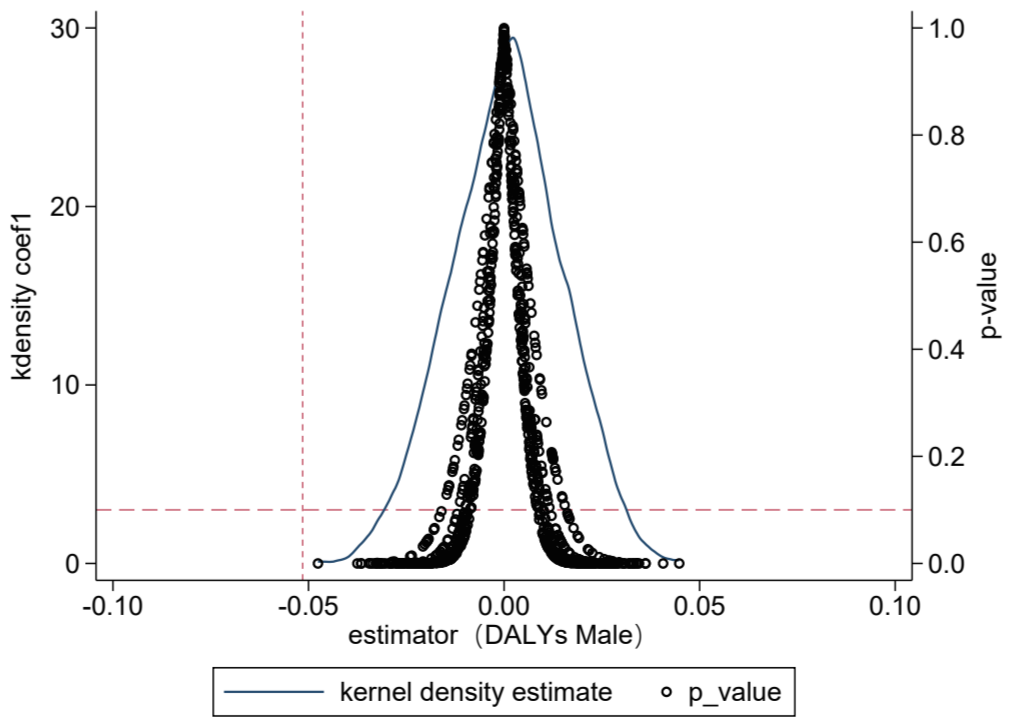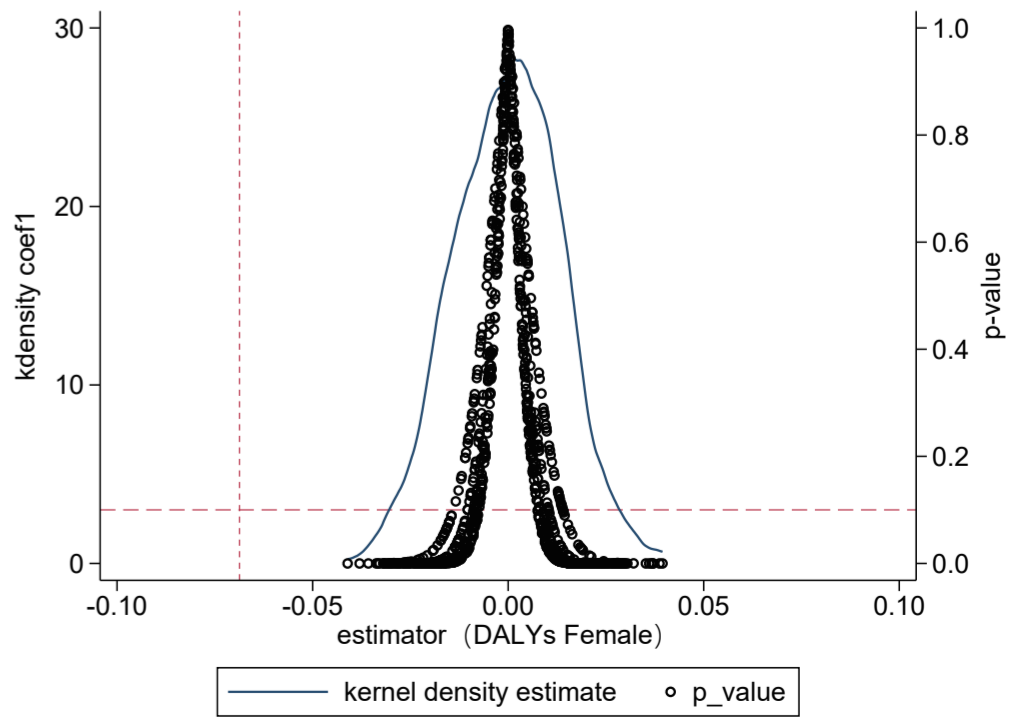

Supplement: SUPPLEMENTARY FIGURE S1 — Effect of “out of the shadows: a global campaign against epilepsy” on global idiopathic epilepsy DALY rates and placebo tests. Current refers to the shock point. Dashed lines represent the 95% confidence intervals. The red vertical dashed line represents the true coefficient, and the red horizontal dashed line represents a p-value of 0.1. [file Image_1.pdf]

Both

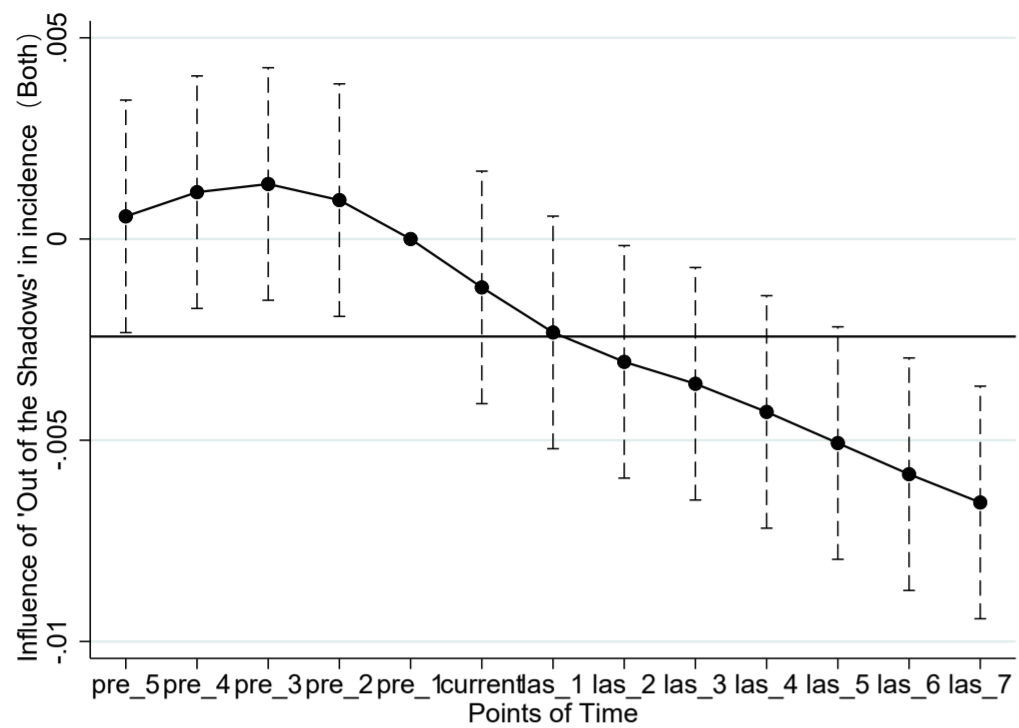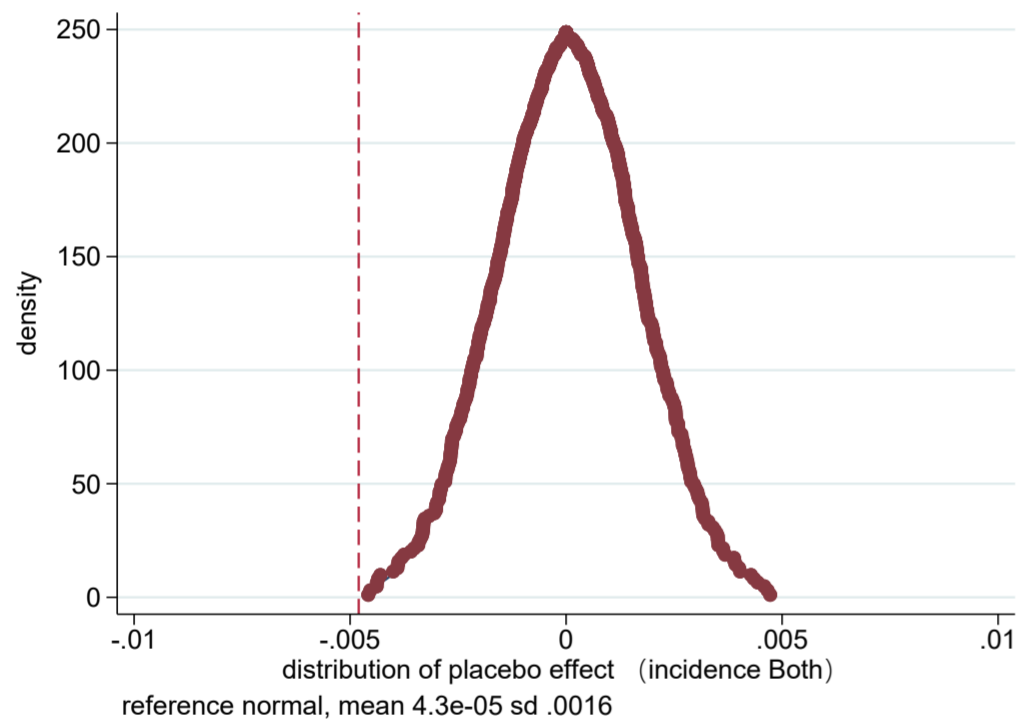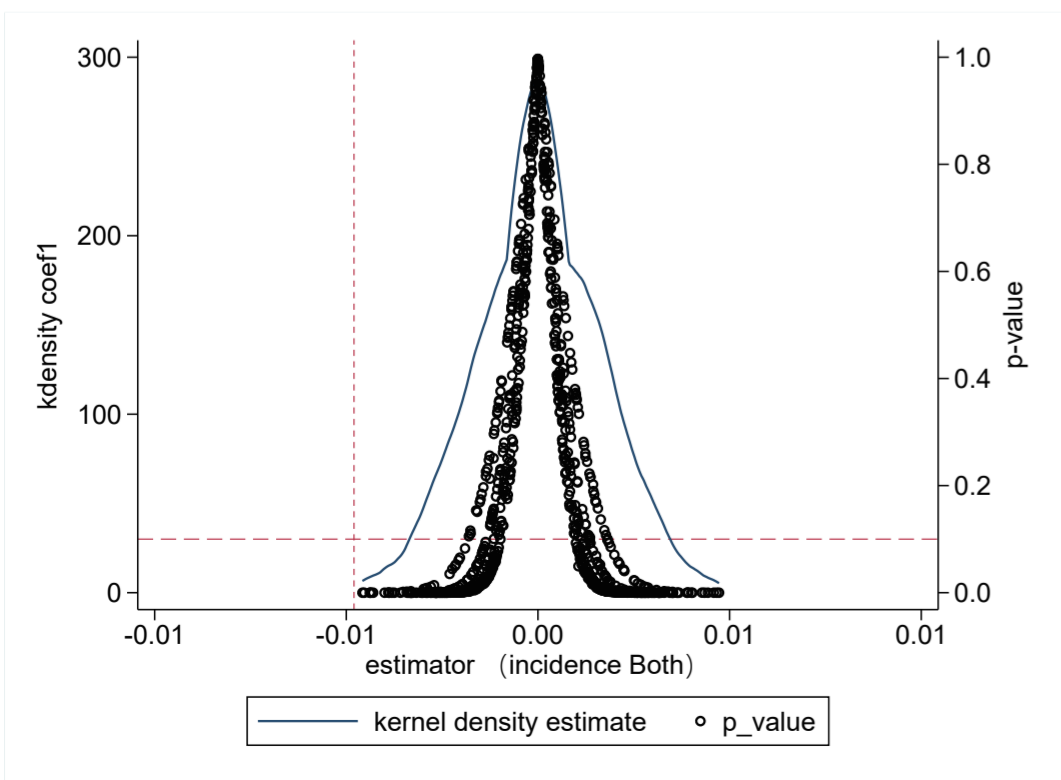

Male

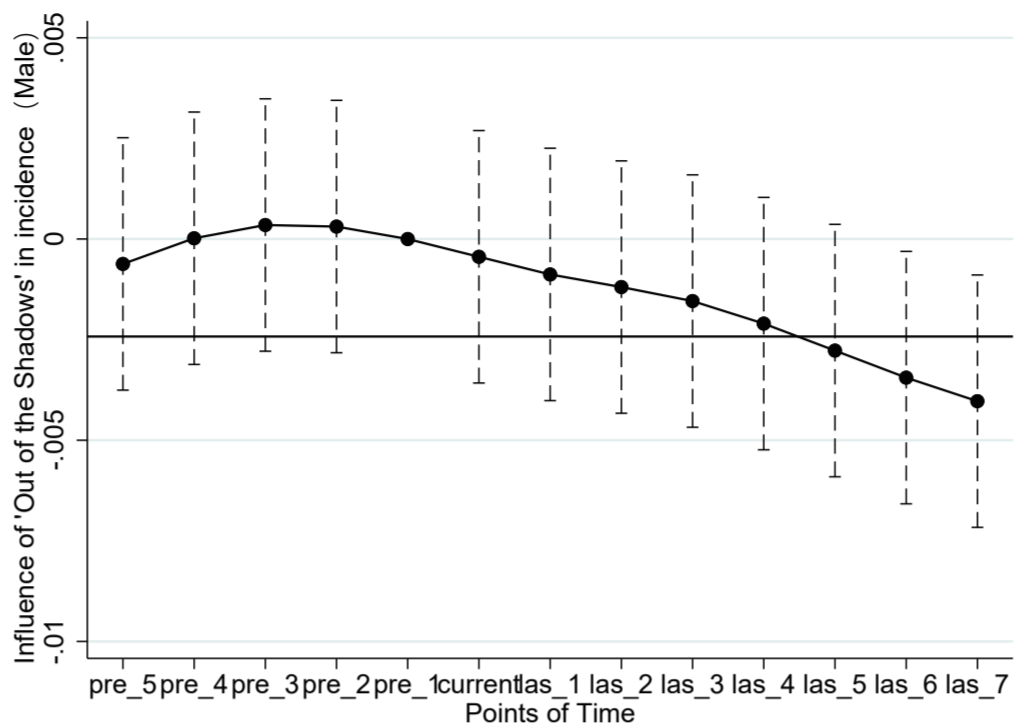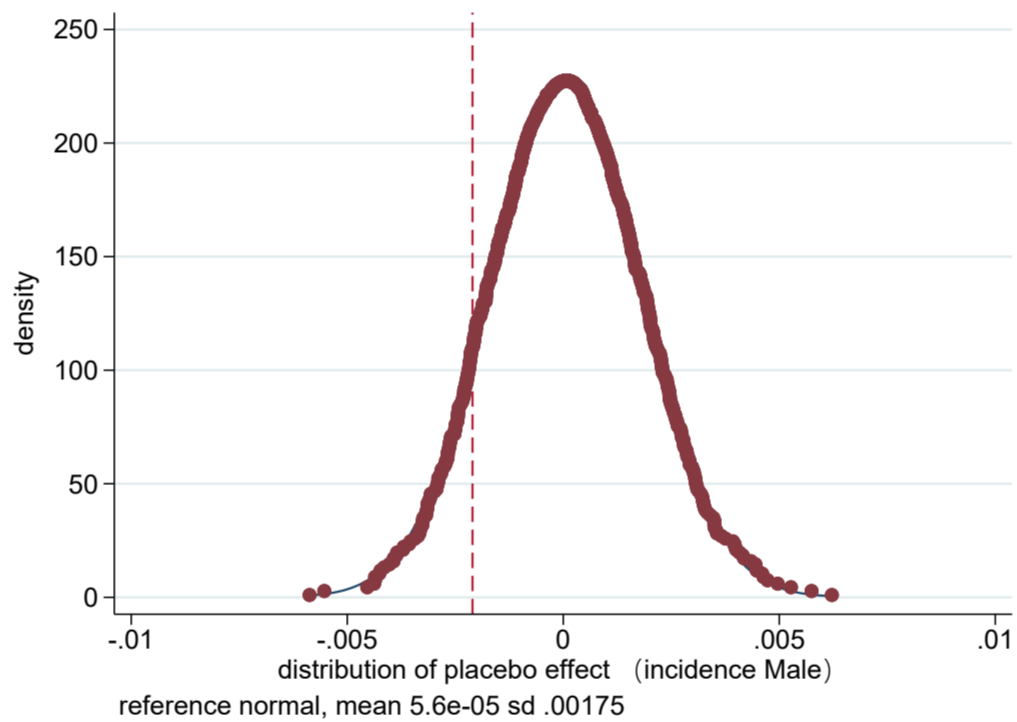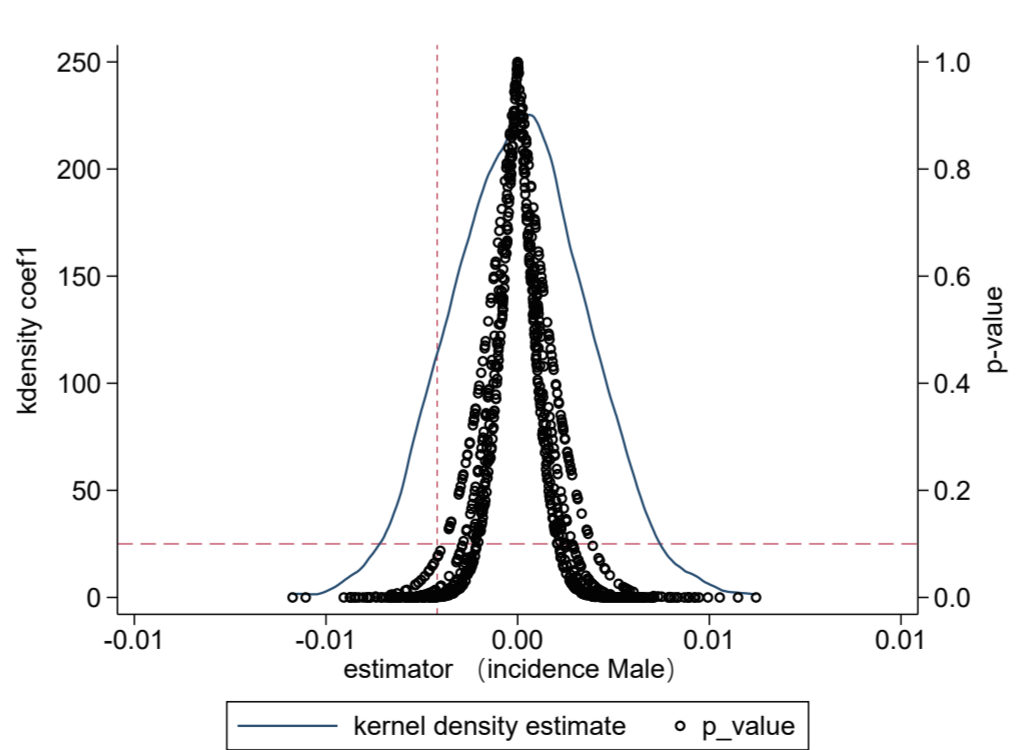

Female

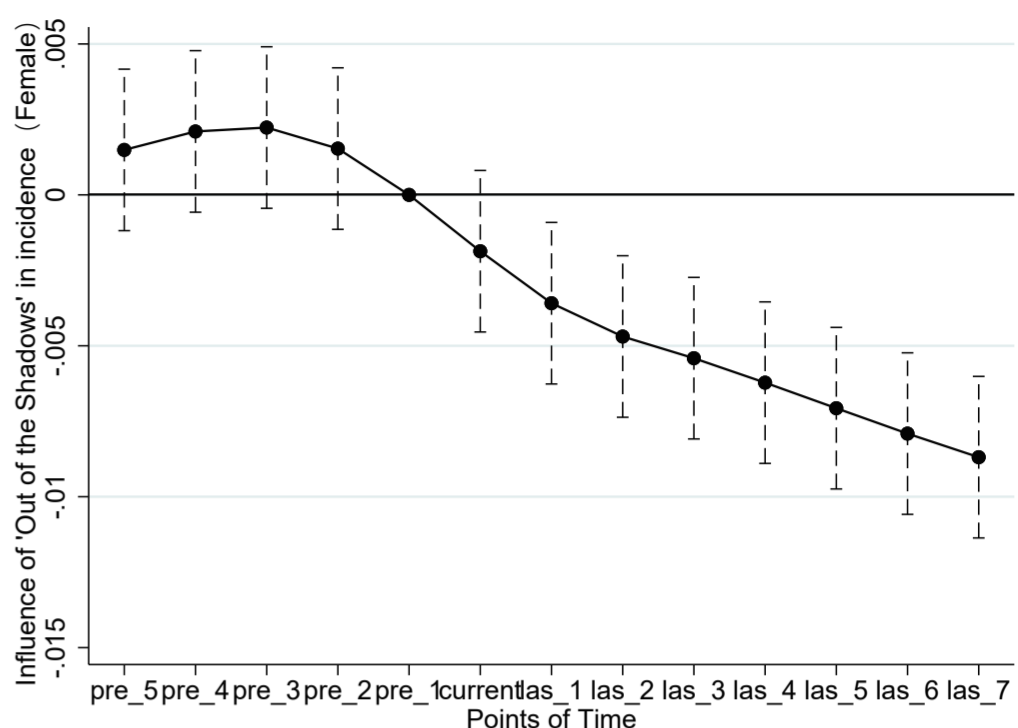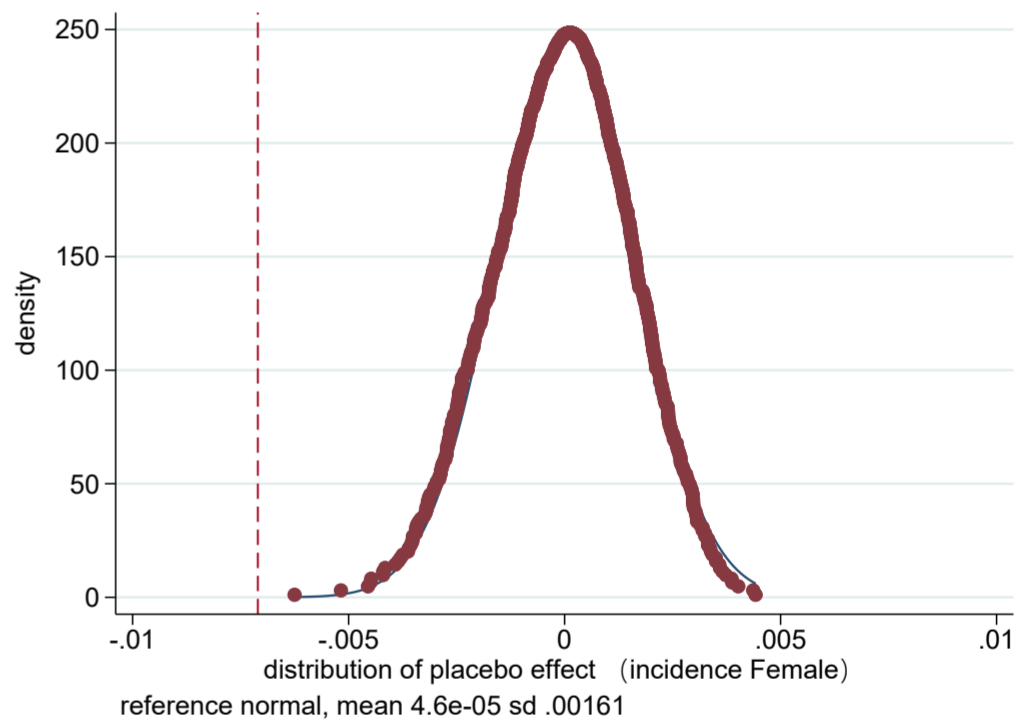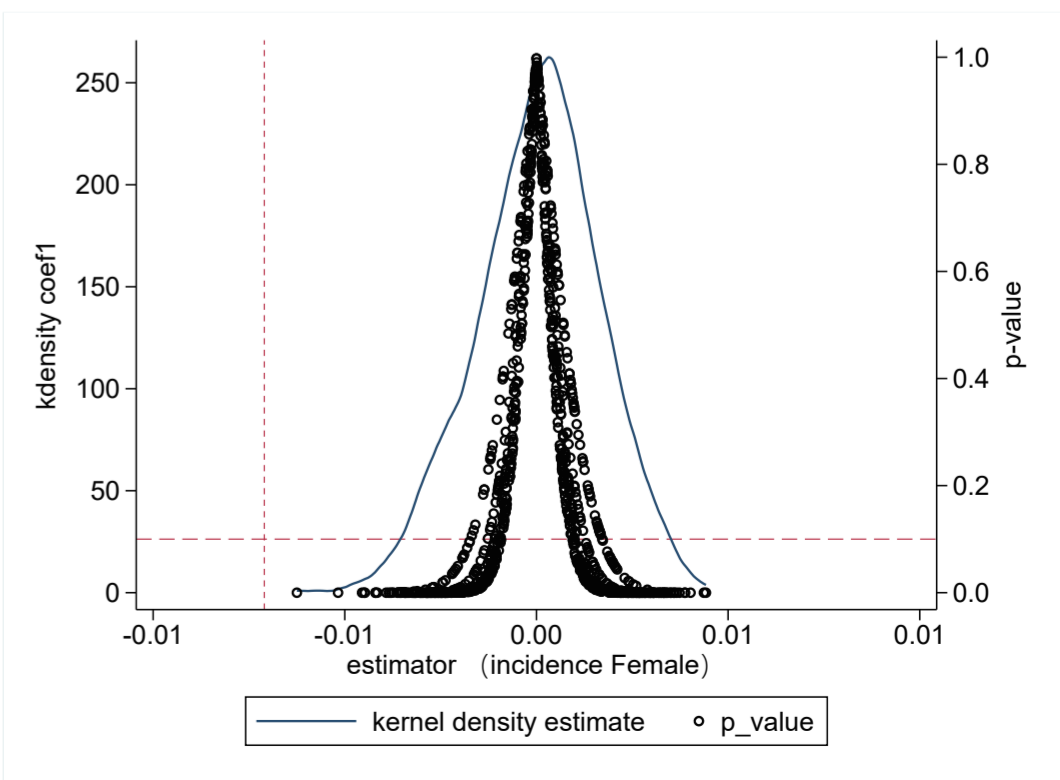

Supplement: SUPPLEMENTARY FIGURE S2 — Effect of “out of the shadows: a global campaign against epilepsy” on global idiopathic epilepsy incidence rates and placebo tests. Current refers to the shock point. Dashed lines represent the 95% confidence intervals. The red vertical dashed line represents the true coefficient, and the red horizontal dashed line represents a p-value of 0.1. [file Image_2.pdf]

Both

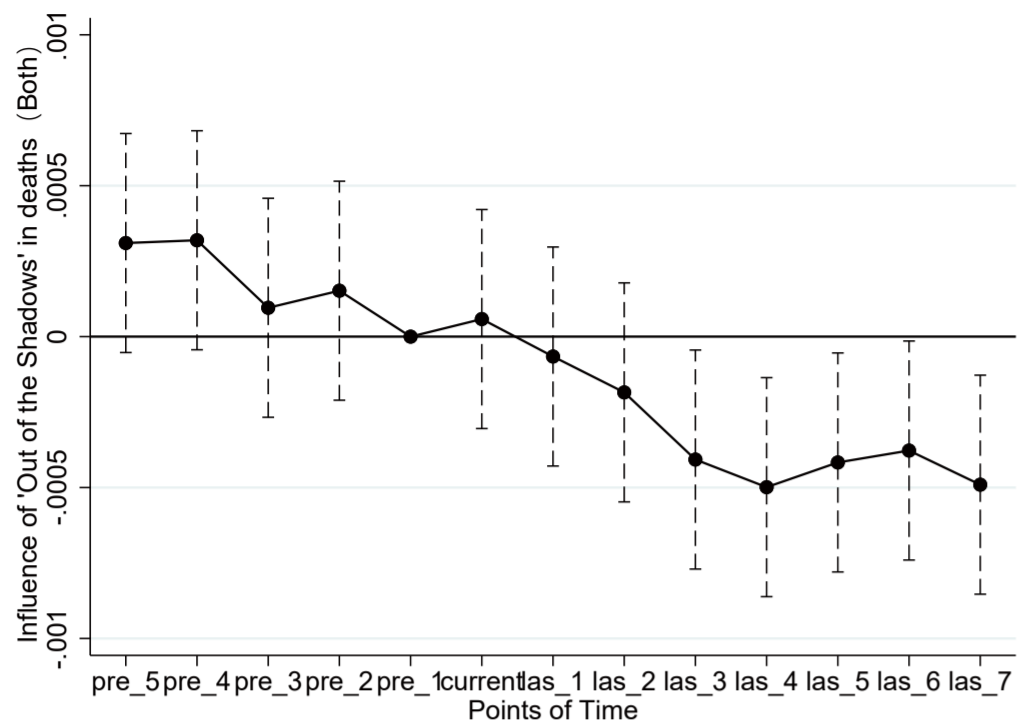

Male

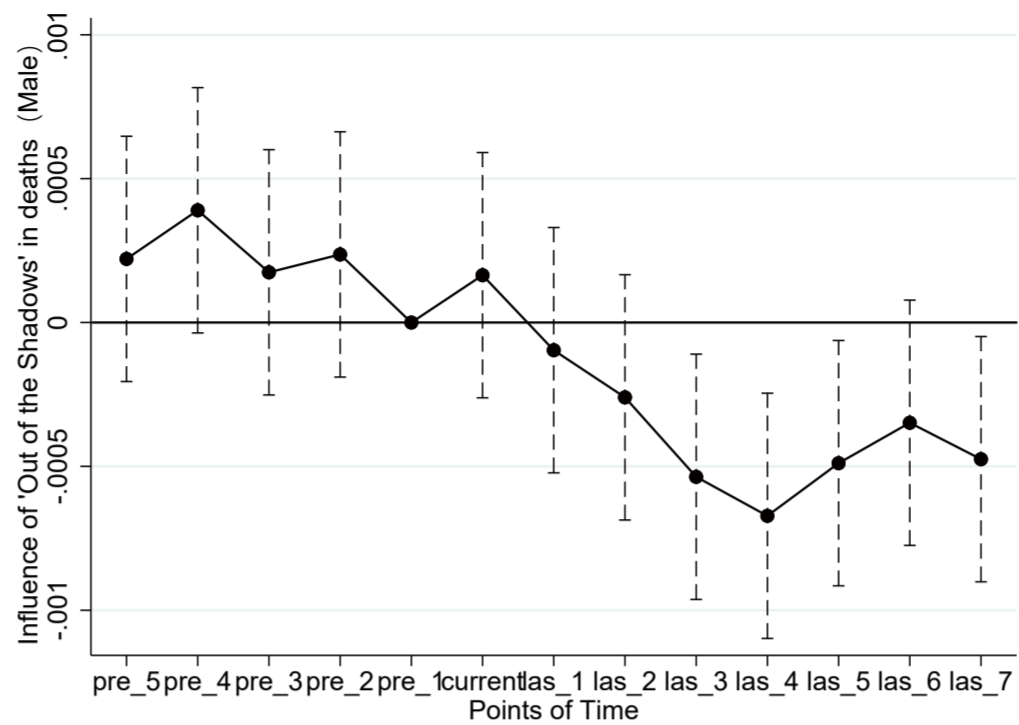

Female

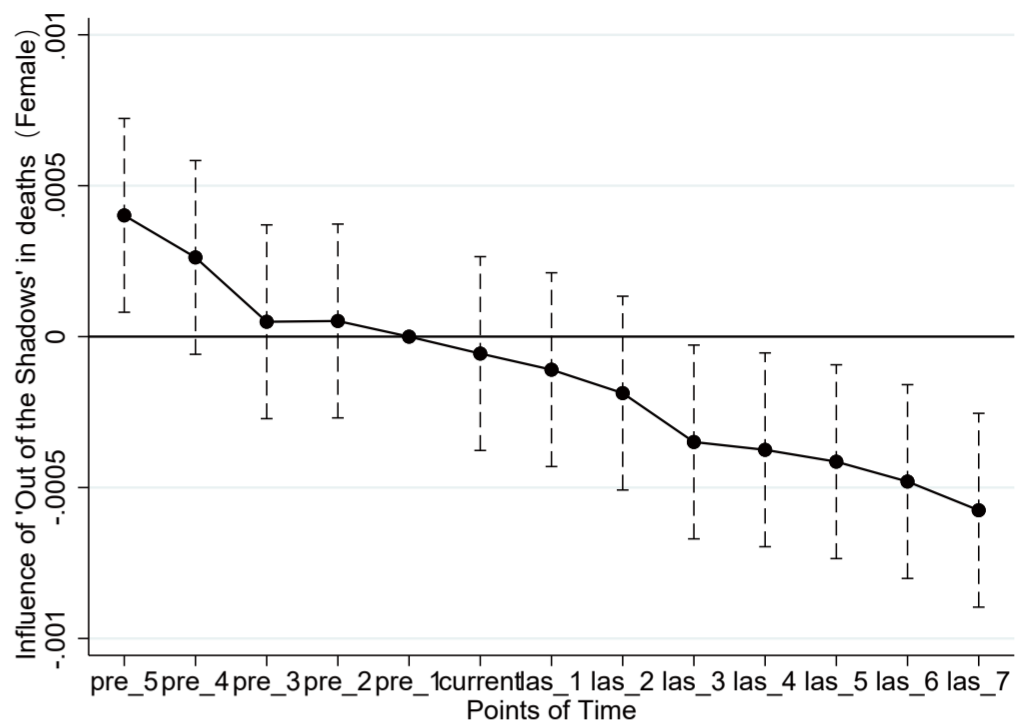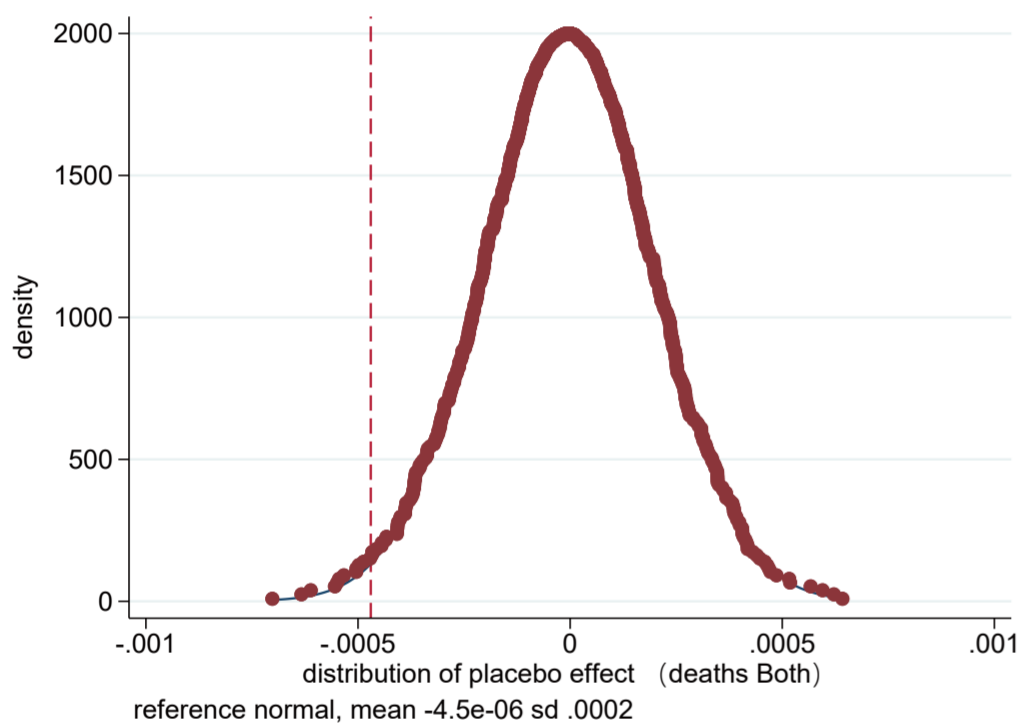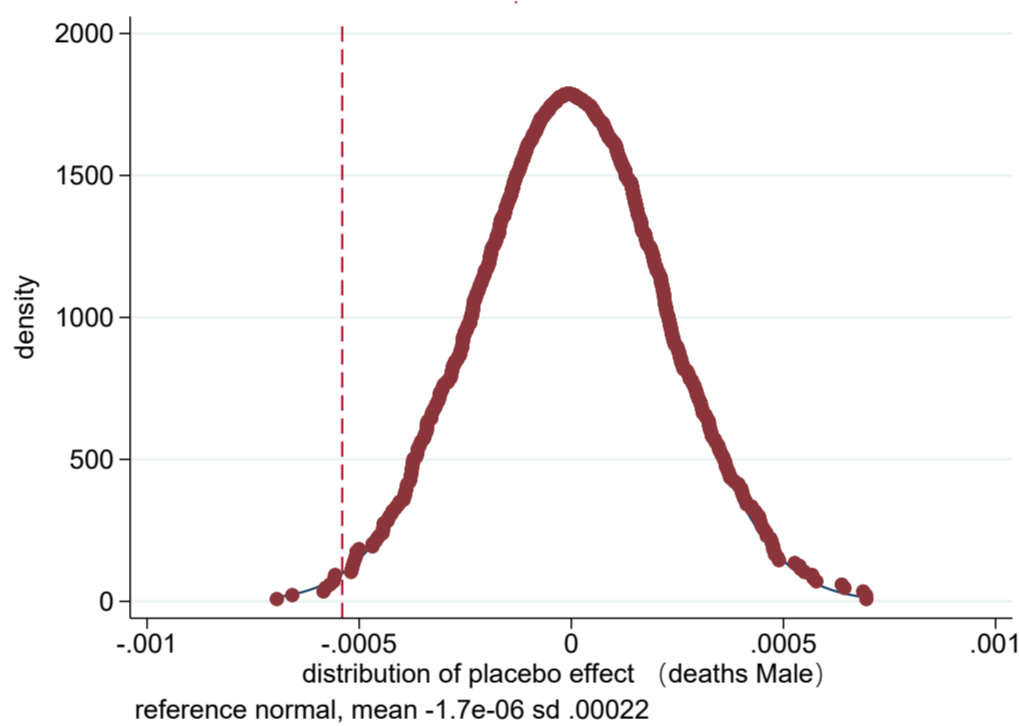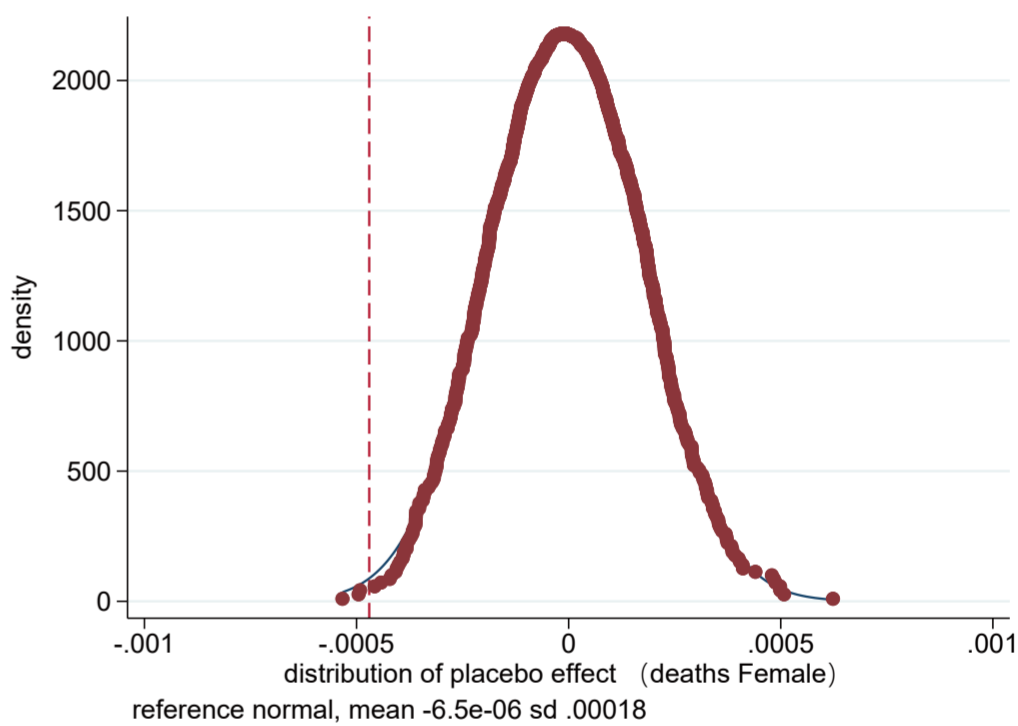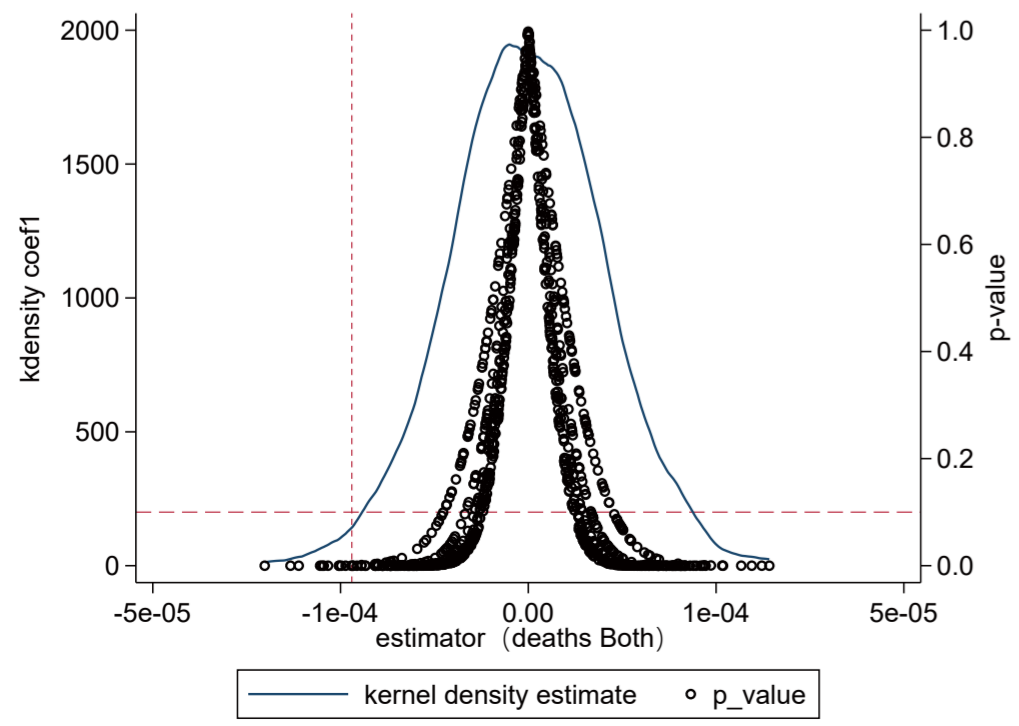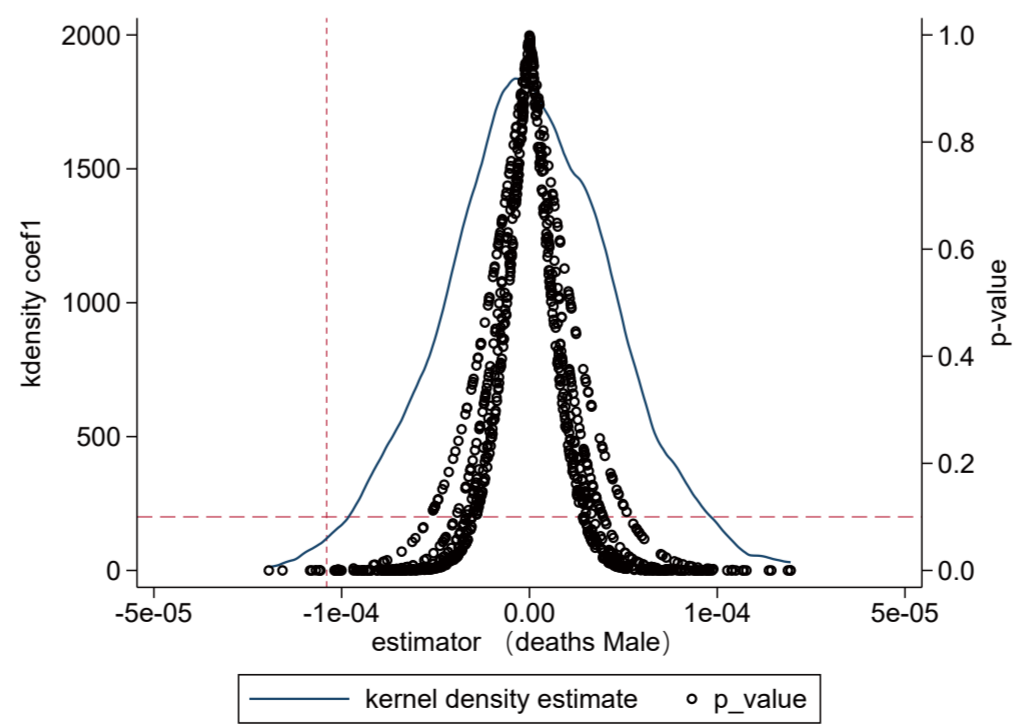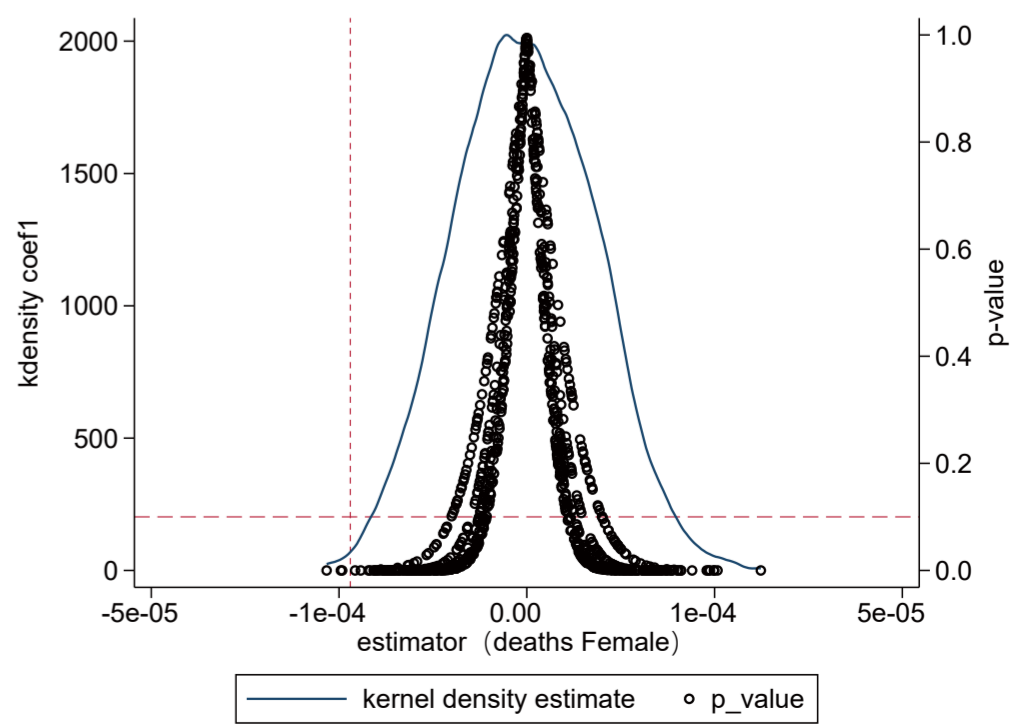

Supplement: SUPPLEMENTARY FIGURE S3 — Effects of “out of the shadows: a global campaign against epilepsy” on global idiopathic epilepsy mortality rates and placebo tests. Current refers to the shock point. Dashed lines represent the 95% confidence intervals. The red vertical dashed line represents the true coefficient, and the red horizontal dashed line represents a p-value of 0.1. [file Image_3.pdf]

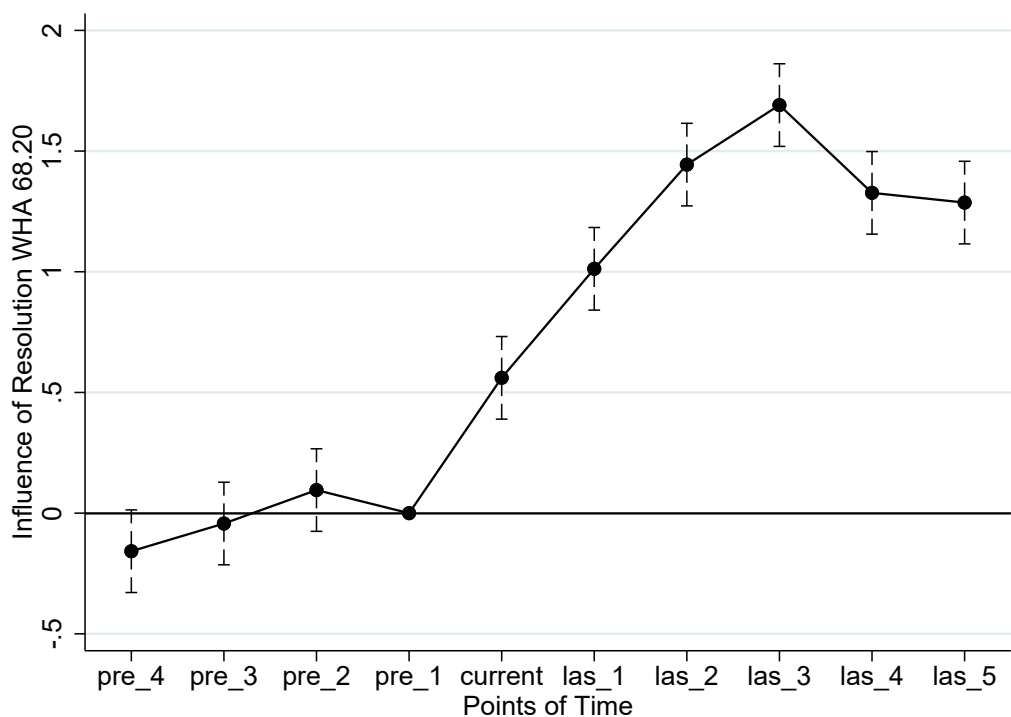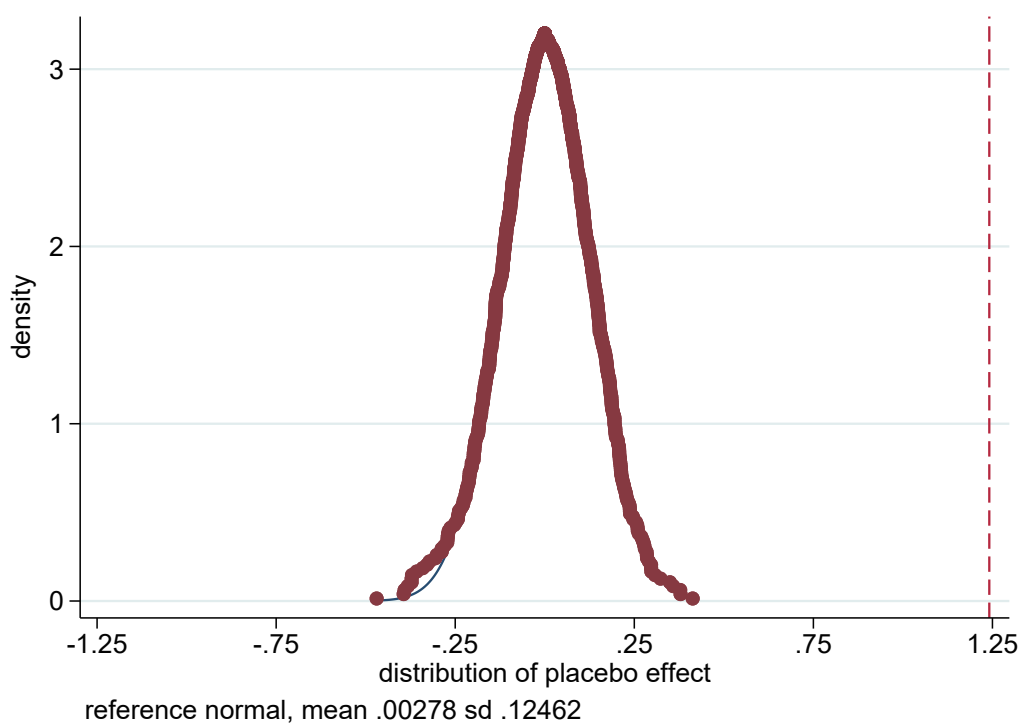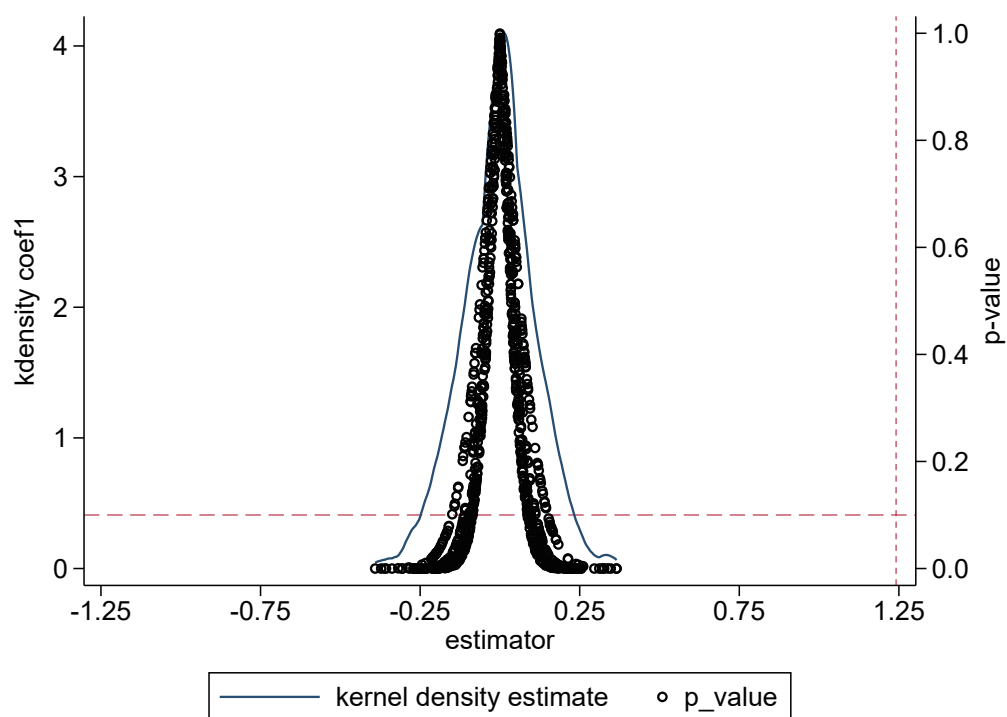

Supplement: SUPPLEMENTARY FIGURE S4 — Effect of revolution WHA 68.20 on global idiopathic epilepsy DALY rates and placebo tests. Current refers to the shock point. Dashed lines represent the 95% confidence intervals. The red vertical dashed line represents the true coefficient, and the red horizontal dashed line represents a p-value of 0.1. [file Image_4.pdf]
