# Supplementary material for: Impact of social factors and health campaigns on the burden of idiopathic epilepsy: an inequality, decomposition, generalized and synthetic difference-in-differences study
Source: Front Public Health. 2025 Jun 2;13:1598497. doi: 10.3389/fpubh.2025.1598497 (PMC12171163; doi:10.3389/fpubh.2025.1598497)
Supplement: Supplementary file 5 [file Table_1.docx]

# Supplementary Methods

## Cross-country health inequality analysis

The SII was calculated by regressing national mortality rates and DALY rates for all-age populations on the relative position scale of SDI. This scale is defined by the midpoint of cumulative population strata ranked by per capita gross domestic product. Heteroscedasticity was accounted for using a weighted regression model, and the log transformation of relative social position values was applied to address non-linearity due to marginal utility. After ranking populations by income and mortality/DALY disease burden, the CIX was calculated by fitting the Lorenz concentration curve and numerically integrating the area under the curve(1).

## Generalized Difference-in-Differences

GDID extends the DID method by incorporating associated variables, allowing causal effects to be identified under conditions different from those typically required by DID(2).

For DID, during the post-treatment period t1, the average effect of treatment on the treated (ATT) is defined as:

$$E\left[ Y_{1}\left( i,t_{1} \right)-Y_{0}\left( {i,t}_{1} \right)|A\left( i \right)=1 \right]$$

Where A(i) represents the treatment status, with A(i)=1 if individual i is treated, and A(i)=0 otherwise.

Y(i,t) is the outcome of interest for individual i at time t, with two observation periods: pre-treatment (t=t0) and post-treatment (t=t1).

When all study subjects are more or less simultaneously exposed to policy interventions—meaning there is only a treatment group without a control group. In such cases, a treatment intensity indicator can be constructed based on the specific shocks each subject experiences. In this scenario, the individual dimension does not change from 0 to 1 but instead reflects continuous variation. This treatment intensity is also referred to as an instrumental variable. Therefore, the policy grouping dummy variable at the individual level can be replaced by a continuous variable that reflects the degree of impact the policy has on each individual.

The specific formula is as follows:

$${ATT}_{GDID}=E\left[ Y\left( t_{1} \right)-Y\left( t_{0} \right)|t\left( Z \right)=1 \right]-E\left[ Y\left( t_{1} \right)-Y\left( t_{0} \right)|t\left( Z \right)=0 \right]$$

Z represents a confounding factor defined as an instrumental variable.

$t\left( Z \right)=E\left( A|Z \right)$ and $E\left[ Y\left( t_{1} \right)-Y\left( t_{0} \right)|t\left( Z \right) \right]$both are assumed to be linear in Z and t(Z), respectively.

The intensity variable is fixed and unchanging. In our study, we use each country's all-ages prevalence rate as an instrumental variable. The working variables in GDID are confounding factors that are associated with the measured factors. YLD is involved in the calculation of prevalence:

$$YLDs=P\times pTss\times DW$$

Prefers to prevalence (global, regional or national), which is determined from population-based studies around the world. Proportion of time in symptomatic state (pTSS) is estimated from population-based studies. Disability weight (DW) has a value between 0 (no disability or health loss) and 1.0 (disability or health loss comparable to being dead). Therefore, we consider prevalence to be a reasonable instrumental variable.We adopted the method used by Chen in 2017, using the difference between the policy shock point and the previous year's prevalence rate as the intensity variable(3).

We used a mixed placebo test to verify the robustness of the baseline regression and confirm the parallel trends assumption. In the mixed placebo test, we randomly reassigned the instrumental variable and selected random years as the shock points for epilepsy-related policies and actions (excluding the first year, as it cannot be the initial shock point). False coefficients were then generated, and density plots and coefficient distribution plots were drawn based on these coefficients. All placebo tests were conducted with 1,000 random samples. The results show that most estimated coefficients are concentrated near zero, and the p-values for most estimates are greater than 0.1 (not significant at the 10% level). This indicates that our estimates are unlikely to have occurred by chance and are less likely to have been affected by other policies or random factors. Furthermore, in most cases, the true values are located at the tail or outside the kernel density curve, indicating that the placebo test results are highly significant.

## Synthetic Difference-in-Differences

In analyzing the Caribbean region, in order to avoid introducing uncertainty into the results from other regions of the Americas influenced by policies, we excluded these regions. In analyzing the United States, we found it difficult to accurately exclude countries affected by the relevant policies. Method from Wenlan Dong et al was used to finish SDID(4). We assumed that our data covered N areas $(n = 1, 2, . . . , N )$, among which the first K areas are treated areas $(n = 1, 2, . . . , K)$ and the rest are non-treated areas $(n = K + 1, K + 2, . . . , N )$. In our research, we just choose one region or country as treated area, which means K=1. We assumed that our data span $T$ periods and there are $T_{0}$ pre-implementation periods. $D_{n}$ is defined to indicate if area n is a treated area. For each area n and year t, we observed its past-month YLD rate, $Y_{n,t}$. We define $Y_{n,t}^{0}$ as the potential outcome in year t for area n as if it is non-treated area. Likewise, $Y_{n,t}^{1}$ is the potential outcome of treated area when $t>T_{0}$.

So we calculate average treatment effect on the treated area n when $t>T_{0}$ as:

$$ATT=E\left[ Y_{n,t}^{1}-Y_{n,t}^{0}|t>T_{0}, D=1 \right]$$

The optimal synthetic weight we used to construct synthetic non-treated areas is as follows:

$$W^{sdid}=\left( \omega_{k+1}^{sdid}, ..., \omega_{n}^{sdid} \right)^{'}$$

It also seeks to balance the time weights Γ=$(\lambda_{1}, \lambda_{2}, ..., \lambda_{T_{0}})$ across both the pre- and post-intervention periods, in order to avoid potential selection bias and confounding factors.

Based on the above two weights, we can rewrite the ATTs as:

$${ATT}_{sdid}=E\left[ Y|t>T_{0},D=1 \right]-E\left[ \hat{\lambda_{t}}Y|t\leq T_{0},D=1 \right]-\sum_{n=1}^{N-K} \hat{\omega_{n}^{sdid}}\left( E\left[ Y|t>T_{0},D=0 \right]-E\left[ \hat{\lambda_{t}}Y|t\leq T_{0},D=0 \right] \right)$$

Here, Y represents the outcome of interest, where $t>T_{0}$. indicates the post-treatment period, and $t\leq T_{0}$. indicates the pre-treatment period. D is a dummy variable indicating whether the region is a demographic region.

# ****Reference****

1. Ordunez P, Martinez R, Soliz P, Giraldo G, Mujica OJ, Nordet P. Rheumatic heart disease burden, trends, and inequalities in the Americas, 1990–2017: A population-based study. *The Lancet Global Health* (2019) 7:e1388–e1397. doi: 10.1016/S2214-109X(19)30360-2

2. Richardson DB, Ye T, Tchetgen Tchetgen EJ. Generalized difference-in-differences. *Epidemiology* (2023) 34:167–174. doi: 10.1097/EDE.0000000000001568

3. Chen SX. The effect of a fiscal squeeze on tax enforcement: Evidence from a natural experiment in China. *Journal of Public Economics* (2017) 147:62–76. doi: 10.1016/j.jpubeco.2017.01.001

4. W D, X Z, S L, X Z, Z L, M G, Y Y, Ye F, H L, J Q, et al. Effect of the national integrated demonstration area for the prevention and control of noncommunicable diseases programme on behavioural risk factors in China: a synthetic difference-in-differences study. *The Lancet regional health Western Pacific* (2024) 50: doi: 10.1016/j.lanwpc.2024.101167
