# Supplementary material for: Impact of social factors and health campaigns on the burden of idiopathic epilepsy: an inequality, decomposition, generalized and synthetic difference-in-differences study
Source: Front Public Health. 2025 Jun 2;13:1598497. doi: 10.3389/fpubh.2025.1598497 (PMC12171163; doi:10.3389/fpubh.2025.1598497)
Supplement: Supplementary file 6 [file Table_2.docx]

Supplementary table1: Decomposition of DALYs changes.

| **factor** | **SDI** | **Both** | | **Female** | | **Male** | |
| --- | --- | --- | --- | --- | --- | --- | --- |
|  |  | **values** | **Per(%)** | **values** | **Per(%)** | **values** | **Per(%)** |
| Aging | Global | -419494.6341 | -16.79 | -235857.0358 | -23.54 | -171175.9861 | -11.43 |
| Aging | High SDI | 22085.29223 | 7.65 | 7087.756495 | 4.86 | 19651.9955 | 13.77 |
| Aging | High-middle SDI | -72674.11516 | 21.38 | -38158.32565 | 26.07 | -30322.73052 | 15.67 |
| Aging | Low SDI | 13653.12225 | 0.9 | -2742.198749 | -0.49 | 15771.04967 | 1.64 |
| Aging | Low-middle SDI | -56560.85562 | -5.36 | -43693.46525 | -9.37 | -12324.83127 | -2.09 |
| Aging | Middle SDI | -166714.0203 | 696.32 | -82935.87916 | 393.17 | -80858.7622 | 2839.2 |
| Epidemiological_change | Global | -2040641.183 | -81.66 | -936806.2406 | -93.5 | -1110802.425 | -74.2 |
| Epidemiological_change | High SDI | -18741.54176 | -6.49 | 19526.75339 | 13.39 | -45008.37767 | -31.53 |
| Epidemiological_change | High-middle SDI | -598333.9292 | 176.03 | -247421.707 | 169.06 | -356465.3097 | 184.17 |
| Epidemiological_change | Low SDI | -516015.2352 | -34 | -275210.2527 | -49.43 | -237330.1261 | -24.7 |
| Epidemiological_change | Low-middle SDI | -697436.591 | -66.12 | -326386.2633 | -70.02 | -367391.319 | -62.41 |
| Epidemiological_change | Middle SDI | -1113530.422 | 4650.94 | -503113.8068 | 2385.09 | -608671.0342 | 21372.33 |
| Population | Global | 4959009.522 | 198.45 | 2174582.482 | 217.04 | 2778932.911 | 185.64 |
| Population | High SDI | 285220.3607 | 98.84 | 119206.1522 | 81.75 | 168099.8311 | 117.76 |
| Population | High-middle SDI | 331110.0099 | -97.41 | 139231.7013 | -95.14 | 193238.3372 | -99.84 |
| Population | Low SDI | 2019867.829 | 133.1 | 834768.6536 | 149.92 | 1182248.591 | 123.06 |
| Population | Low-middle SDI | 1808862.062 | 171.48 | 836229.0328 | 179.39 | 968431.4611 | 164.5 |
| Population | Middle SDI | 1256302.399 | -5247.26 | 564955.5828 | -2678.26 | 686681.8563 | -24111.53 |

Supplementary table2: Decomposition of prevalence changes.

| **factor** | **SDI** | **Both** | | **Female** | | **Male** | |
| --- | --- | --- | --- | --- | --- | --- | --- |
|  |  | **values** | **per** | **values** | **per** | **values** | **per** |
| Aging | Global | 31065.05912 | 0.35 | 13171.66142 | 0.31 | 31959.81678 | 0.7 |
| Aging | High SDI | 129857.289 | 9.85 | 53715.94211 | 8.4 | 88476.58645 | 13.04 |
| Aging | High-middle SDI | 17545.94698 | 2.45 | 2923.629904 | 0.89 | 20232.18242 | 5.21 |
| Aging | Low SDI | 5967.463182 | 0.32 | 2381.00541 | 0.27 | 3748.199198 | 0.38 |
| Aging | Low-middle SDI | -43169.23188 | -1.74 | -9813.575371 | -0.79 | -31445.02358 | -2.55 |
| Aging | Middle SDI | -86771.88097 | -3.47 | -26198.99474 | -2.18 | -57530.51347 | -4.42 |
| Epidemiological_change | Global | 1285009.566 | 14.44 | 642478.38 | 14.91 | 631035.0919 | 13.75 |
| Epidemiological_change | High SDI | 430628.7915 | 32.68 | 243657.5143 | 38.11 | 172209.6469 | 25.38 |
| Epidemiological_change | High-middle SDI | 64976.47876 | 9.06 | 31777.8574 | 9.66 | 26684.16663 | 6.88 |
| Epidemiological_change | Low SDI | -38815.78149 | -2.07 | -20481.85494 | -2.3 | -17723.14307 | -1.8 |
| Epidemiological_change | Low-middle SDI | 326848.7486 | 13.17 | 184606.8813 | 14.79 | 142480.5282 | 11.55 |
| Epidemiological_change | Middle SDI | 493192.5633 | 19.71 | 187825.4086 | 15.65 | 304059.2192 | 23.35 |
| Population | Global | 7583962 | 85.21 | 3653992.081 | 84.79 | 3927399.594 | 85.56 |
| Population | High SDI | 757412.5355 | 57.47 | 342009.0584 | 53.49 | 417829.8677 | 61.58 |
| Population | High-middle SDI | 634319.9456 | 88.49 | 294153.0055 | 89.45 | 341071.5294 | 87.91 |
| Population | Low SDI | 1909910.283 | 101.75 | 909681.9692 | 102.03 | 999455.7883 | 101.42 |
| Population | Low-middle SDI | 2197788.346 | 88.57 | 1072992.426 | 85.99 | 1122646.626 | 91 |
| Population | Middle SDI | 2095906.466 | 83.76 | 1038318.471 | 86.53 | 1055853.558 | 81.07 |

Supplementary table3: Decomposition of incidence changes.

| **factor** | **SDI** | Both | | Female | | Male | |
| --- | --- | --- | --- | --- | --- | --- | --- |
|  |  | **values** | **per** | **values** | **per** | **values** | **per** |
| Aging | Global | -195751.6578 | -17 | -92708.93606 | -17.08 | -101278.0171 | -16.64 |
| Aging | High SDI | -27193.23358 | -21.87 | -12704.87925 | -21.02 | -13483.67506 | -21.1 |
| Aging | High-middle SDI | -41835.09532 | -55.17 | -21213.56127 | -63.65 | -20181.7405 | -47.49 |
| Aging | Low SDI | -11932.72972 | -3.76 | -5643.729802 | -3.88 | -6184.156255 | -3.59 |
| Aging | Low-middle SDI | -52264.64705 | -15.65 | -24072.66303 | -14.91 | -27775.23398 | -16.11 |
| Aging | Middle SDI | -95182.72156 | -31.81 | -45566.88989 | -32.1 | -49086.1569 | -31.2 |
| Epidemiological_change | Global | 307490.1885 | 26.7 | 143720.9411 | 26.47 | 162550.3354 | 26.71 |
| Epidemiological_change | High SDI | 53819.50343 | 43.28 | 30179.92412 | 49.92 | 22187.04676 | 34.73 |
| Epidemiological_change | High-middle SDI | 38584.11307 | 50.89 | 18055.64052 | 54.17 | 19971.22808 | 47 |
| Epidemiological_change | Low SDI | 21043.41926 | 6.62 | 9339.994818 | 6.42 | 11804.74167 | 6.85 |
| Epidemiological_change | Low-middle SDI | 76070.16116 | 22.79 | 37934.77455 | 23.49 | 38216.50108 | 22.17 |
| Epidemiological_change | Middle SDI | 110932.201 | 37.07 | 47862.86272 | 33.72 | 62831.76632 | 39.94 |
| Population | Global | 1039806.446 | 90.3 | 491885.0568 | 90.6 | 547375.5965 | 89.93 |
| Population | High SDI | 97716.7848 | 78.59 | 42975.89474 | 71.09 | 55188.74334 | 86.38 |
| Population | High-middle SDI | 79075.67974 | 104.29 | 36488.82694 | 109.47 | 42704.30373 | 100.5 |
| Population | Low SDI | 308636.785 | 97.13 | 141753.4871 | 97.46 | 166677.1371 | 96.74 |
| Population | Low-middle SDI | 310049.2974 | 92.87 | 147628.944 | 91.42 | 161922.4889 | 93.94 |
| Population | Middle SDI | 283494.7351 | 94.74 | 139639.5156 | 98.38 | 143563.1166 | 91.26 |

Supplementary table4: Decomposition of mortality changes.

| **factor** | **SDI** | **Both** | | **Female** | | **Male** | |
| --- | --- | --- | --- | --- | --- | --- | --- |
|  |  | **values** | **per** | **values** | **per** | **values** | **per** |
| Aging | Global | 10774.16496 | 30.35 | 3812.669143 | 26.61 | 7322.708818 | 34.6 |
| Aging | High SDI | 2747.357272 | 35.06 | 1267.583496 | 30.19 | 1592.564184 | 43.77 |
| Aging | High-middle SDI | 1621.810337 | -99.37 | 646.9069805 | -263.57 | 1078.095565 | -77.75 |
| Aging | Low SDI | 782.552993 | 4.68 | 236.9151562 | 4.64 | 464.7432405 | 4 |
| Aging | Low-middle SDI | 3750.677289 | 29.15 | 1641.684065 | 29.27 | 2066.408106 | 28.47 |
| Aging | Middle SDI | 3348.705598 | -1032.69 | 1261.485768 | -360.53 | 2091.929873 | 8163.29 |
| Epidemiological_change | Global | -22781.89381 | -64.18 | -8552.974359 | -59.69 | -14523.67495 | -68.62 |
| Epidemiological_change | High SDI | 2279.815538 | 29.09 | 1791.82638 | 42.68 | 348.5960251 | 9.58 |
| Epidemiological_change | High-middle SDI | -6274.446964 | 384.44 | -2054.906386 | 837.25 | -4344.177034 | 313.28 |
| Epidemiological_change | Low SDI | -6638.372703 | -39.73 | -3206.606994 | -62.85 | -3296.119892 | -28.4 |
| Epidemiological_change | Low-middle SDI | -9029.080794 | -70.17 | -4108.727097 | -73.25 | -4816.486243 | -66.37 |
| Epidemiological_change | Middle SDI | -14008.80055 | 4320.12 | -5744.181043 | 1641.69 | -8198.04497 | -31991.03 |
| Population | Global | 47502.34521 | 133.83 | 19069.24208 | 133.08 | 28366.64562 | 134.02 |
| Population | High SDI | 2809.755552 | 35.85 | 1139.234241 | 27.13 | 1697.124035 | 46.65 |
| Population | High-middle SDI | 3020.529306 | -185.07 | 1162.563614 | -473.67 | 1879.409938 | -135.53 |
| Population | Low SDI | 22563.53266 | 135.05 | 8071.834494 | 158.2 | 14436.94694 | 124.4 |
| Population | Low-middle SDI | 18145.11747 | 141.02 | 8076.491204 | 143.98 | 10007.34393 | 137.89 |
| Population | Middle SDI | 10335.82632 | -3187.43 | 4132.800569 | -1181.16 | 6131.741168 | 23927.75 |

Supplementary table5: local drift of DALYs.

| **sex** | **Both** | | | **Male** | | | **Female** | | |
| --- | --- | --- | --- | --- | --- | --- | --- | --- | --- |
| **Age** | **Percent per Year** | **CILo** | **CIHi** | **Percent per Year** | **CILo** | **CIHi** | **Percent per Year** | **CILo** | **CIHi** |
| 0 to 4 | -1.18 | -1.25 | -1.11 | -0.95 | -1.02 | -0.88 | -1.45 | -1.54 | -1.37 |
| 5 to 9 | -0.90 | -0.96 | -0.85 | -0.79 | -0.85 | -0.73 | -1.04 | -1.11 | -0.97 |
| 10 to 14 | -0.70 | -0.75 | -0.65 | -0.64 | -0.69 | -0.58 | -0.78 | -0.85 | -0.72 |
| 15 to 19 | -0.58 | -0.63 | -0.53 | -0.55 | -0.60 | -0.50 | -0.63 | -0.69 | -0.56 |
| 20 to 24 | -0.51 | -0.56 | -0.45 | -0.49 | -0.54 | -0.44 | -0.54 | -0.61 | -0.47 |
| 25 to 29 | -0.49 | -0.55 | -0.44 | -0.44 | -0.50 | -0.39 | -0.56 | -0.63 | -0.49 |
| 30 to 34 | -0.57 | -0.63 | -0.51 | -0.48 | -0.54 | -0.42 | -0.69 | -0.77 | -0.62 |
| 35 to 39 | -0.63 | -0.70 | -0.57 | -0.53 | -0.60 | -0.47 | -0.76 | -0.84 | -0.67 |
| 40 to 44 | -0.69 | -0.76 | -0.62 | -0.61 | -0.68 | -0.54 | -0.80 | -0.89 | -0.70 |
| 45 to 49 | -0.73 | -0.80 | -0.65 | -0.67 | -0.75 | -0.59 | -0.78 | -0.88 | -0.68 |
| 50 to 54 | -0.72 | -0.80 | -0.63 | -0.70 | -0.78 | -0.61 | -0.72 | -0.83 | -0.61 |
| 55 to 59 | -0.60 | -0.69 | -0.51 | -0.62 | -0.71 | -0.53 | -0.56 | -0.68 | -0.44 |
| 60 to 64 | -0.47 | -0.57 | -0.37 | -0.53 | -0.63 | -0.43 | -0.39 | -0.52 | -0.27 |
| 65 to 69 | -0.28 | -0.39 | -0.17 | -0.43 | -0.55 | -0.32 | -0.13 | -0.26 | 0.01 |
| 70 to 74 | -0.08 | -0.21 | 0.05 | -0.35 | -0.48 | -0.21 | 0.15 | 0.00 | 0.31 |
| 75 to 79 | 0.16 | 0.01 | 0.32 | -0.21 | -0.38 | -0.04 | 0.46 | 0.27 | 0.64 |
| 80 to 84 | 0.40 | 0.20 | 0.60 | -0.05 | -0.28 | 0.18 | 0.71 | 0.48 | 0.95 |
| 85 to 89 | 0.65 | 0.35 | 0.95 | 0.14 | -0.21 | 0.49 | 0.96 | 0.63 | 1.29 |
| 90 to 94 | 0.89 | 0.37 | 1.41 | 0.31 | -0.34 | 0.96 | 1.20 | 0.65 | 1.75 |
| 95 to 99 | 1.03 | -0.06 | 2.14 | 0.36 | -1.08 | 1.84 | 1.34 | 0.22 | 2.47 |

Supplementary table6: local drift of incidence.

| **sex** | **Both** | | | **Male** | | | **Female** | | |
| --- | --- | --- | --- | --- | --- | --- | --- | --- | --- |
| **Age** | **Percent per Year** | **CILo** | **CIHi** | **Percent per Year** | **CILo** | **CIHi** | **Percent per Year** | **CILo** | **CIHi** |
| 0 to 4 | 0.07 | 0.03 | 0.12 | 0.08 | 0.03 | 0.13 | 0.06 | 0.01 | 0.11 |
| 5 to 9 | 0.22 | 0.18 | 0.25 | 0.24 | 0.20 | 0.28 | 0.19 | 0.15 | 0.23 |
| 10 to 14 | 0.24 | 0.20 | 0.28 | 0.28 | 0.24 | 0.32 | 0.20 | 0.15 | 0.24 |
| 15 to 19 | 0.22 | 0.18 | 0.25 | 0.28 | 0.24 | 0.32 | 0.14 | 0.10 | 0.19 |
| 20 to 24 | 0.21 | 0.17 | 0.26 | 0.29 | 0.25 | 0.33 | 0.12 | 0.08 | 0.17 |
| 25 to 29 | 0.20 | 0.15 | 0.24 | 0.28 | 0.23 | 0.32 | 0.11 | 0.07 | 0.16 |
| 30 to 34 | 0.14 | 0.09 | 0.19 | 0.20 | 0.15 | 0.25 | 0.08 | 0.03 | 0.13 |
| 35 to 39 | 0.12 | 0.06 | 0.17 | 0.14 | 0.09 | 0.20 | 0.09 | 0.03 | 0.15 |
| 40 to 44 | 0.10 | 0.04 | 0.16 | 0.09 | 0.03 | 0.15 | 0.12 | 0.05 | 0.18 |
| 45 to 49 | 0.10 | 0.04 | 0.17 | 0.07 | 0.00 | 0.14 | 0.15 | 0.08 | 0.22 |
| 50 to 54 | 0.19 | 0.12 | 0.27 | 0.15 | 0.08 | 0.23 | 0.25 | 0.17 | 0.33 |
| 55 to 59 | 0.36 | 0.28 | 0.43 | 0.30 | 0.22 | 0.38 | 0.42 | 0.34 | 0.51 |
| 60 to 64 | 0.48 | 0.40 | 0.57 | 0.40 | 0.31 | 0.49 | 0.58 | 0.48 | 0.68 |
| 65 to 69 | 0.59 | 0.49 | 0.68 | 0.44 | 0.34 | 0.55 | 0.72 | 0.61 | 0.83 |
| 70 to 74 | 0.68 | 0.57 | 0.79 | 0.49 | 0.37 | 0.61 | 0.83 | 0.71 | 0.95 |
| 75 to 79 | 0.80 | 0.66 | 0.93 | 0.59 | 0.44 | 0.74 | 0.94 | 0.80 | 1.08 |
| 80 to 84 | 0.86 | 0.70 | 1.03 | 0.64 | 0.45 | 0.83 | 0.98 | 0.81 | 1.15 |
| 85 to 89 | 0.93 | 0.70 | 1.16 | 0.72 | 0.44 | 1.00 | 1.02 | 0.80 | 1.25 |
| 90 to 94 | 0.96 | 0.60 | 1.32 | 0.79 | 0.31 | 1.27 | 1.03 | 0.69 | 1.36 |
| 95 to 99 | 0.91 | 0.20 | 1.62 | 0.79 | -0.22 | 1.81 | 0.95 | 0.32 | 1.59 |

Supplementary table7: local drift of prevalence.

| **sex** | **Both** | | | **Male** | | | **Female** | | |
| --- | --- | --- | --- | --- | --- | --- | --- | --- | --- |
| **Age** | **Percent per Year** | **CILo** | **CIHi** | **Percent per Year** | **CILo** | **CIHi** | **Percent per Year** | **CILo** | **CIHi** |
| 0 to 4 | -0.26 | -0.33 | -0.20 | -0.30 | -0.35 | -0.24 | -0.22 | -0.30 | -0.15 |
| 5 to 9 | -0.09 | -0.14 | -0.04 | -0.11 | -0.15 | -0.07 | -0.06 | -0.12 | -0.01 |
| 10 to 14 | -0.03 | -0.08 | 0.01 | -0.04 | -0.08 | 0.00 | -0.03 | -0.08 | 0.02 |
| 15 to 19 | -0.04 | -0.08 | 0.01 | -0.01 | -0.05 | 0.03 | -0.07 | -0.12 | -0.02 |
| 20 to 24 | -0.03 | -0.07 | 0.01 | 0.03 | -0.01 | 0.07 | -0.10 | -0.15 | -0.05 |
| 25 to 29 | -0.04 | -0.08 | 0.01 | 0.04 | 0.00 | 0.08 | -0.12 | -0.18 | -0.07 |
| 30 to 34 | -0.10 | -0.14 | -0.05 | -0.02 | -0.06 | 0.03 | -0.19 | -0.24 | -0.13 |
| 35 to 39 | -0.13 | -0.18 | -0.08 | -0.07 | -0.12 | -0.02 | -0.20 | -0.26 | -0.14 |
| 40 to 44 | -0.16 | -0.21 | -0.10 | -0.12 | -0.18 | -0.07 | -0.19 | -0.25 | -0.12 |
| 45 to 49 | -0.15 | -0.21 | -0.09 | -0.14 | -0.20 | -0.09 | -0.15 | -0.22 | -0.08 |
| 50 to 54 | -0.06 | -0.12 | 0.01 | -0.07 | -0.13 | -0.01 | -0.04 | -0.11 | 0.04 |
| 55 to 59 | 0.11 | 0.04 | 0.18 | 0.08 | 0.01 | 0.14 | 0.15 | 0.07 | 0.23 |
| 60 to 64 | 0.25 | 0.18 | 0.32 | 0.20 | 0.14 | 0.27 | 0.30 | 0.22 | 0.38 |
| 65 to 69 | 0.35 | 0.28 | 0.43 | 0.28 | 0.21 | 0.35 | 0.41 | 0.33 | 0.50 |
| 70 to 74 | 0.44 | 0.36 | 0.53 | 0.34 | 0.26 | 0.43 | 0.51 | 0.42 | 0.61 |
| 75 to 79 | 0.57 | 0.47 | 0.67 | 0.45 | 0.35 | 0.55 | 0.63 | 0.52 | 0.74 |
| 80 to 84 | 0.66 | 0.54 | 0.79 | 0.51 | 0.38 | 0.64 | 0.72 | 0.59 | 0.86 |
| 85 to 89 | 0.79 | 0.61 | 0.97 | 0.62 | 0.42 | 0.81 | 0.86 | 0.67 | 1.04 |
| 90 to 94 | 0.95 | 0.65 | 1.26 | 0.77 | 0.41 | 1.12 | 1.02 | 0.72 | 1.33 |
| 95 to 99 | 1.09 | 0.45 | 1.73 | 0.89 | 0.09 | 1.69 | 1.16 | 0.54 | 1.79 |

Supplementary table8: local drift of mortality.

| **sex** | **Both** | | | **Male** | | | **Female** | | |
| --- | --- | --- | --- | --- | --- | --- | --- | --- | --- |
| **Age** | **Percent per Year** | **CILo** | **CIHi** | **Percent per Year** | **CILo** | **CIHi** | **Percent per Year** | **CILo** | **CIHi** |
| 0 to 4 | -1.56 | -1.70 | -1.42 | -1.12 | -1.26 | -0.97 | -2.09 | -2.27 | -1.92 |
| 5 to 9 | -1.30 | -1.42 | -1.17 | -1.02 | -1.15 | -0.89 | -1.64 | -1.80 | -1.49 |
| 10 to 14 | -0.99 | -1.11 | -0.88 | -0.83 | -0.95 | -0.71 | -1.21 | -1.35 | -1.07 |
| 15 to 19 | -0.75 | -0.86 | -0.65 | -0.69 | -0.79 | -0.58 | -0.85 | -0.98 | -0.72 |
| 20 to 24 | -0.59 | -0.69 | -0.49 | -0.59 | -0.69 | -0.49 | -0.61 | -0.75 | -0.48 |
| 25 to 29 | -0.54 | -0.64 | -0.44 | -0.50 | -0.60 | -0.40 | -0.61 | -0.75 | -0.48 |
| 30 to 34 | -0.61 | -0.72 | -0.51 | -0.51 | -0.61 | -0.41 | -0.79 | -0.93 | -0.65 |
| 35 to 39 | -0.71 | -0.81 | -0.60 | -0.58 | -0.68 | -0.48 | -0.91 | -1.06 | -0.77 |
| 40 to 44 | -0.78 | -0.89 | -0.67 | -0.65 | -0.75 | -0.55 | -0.98 | -1.13 | -0.83 |
| 45 to 49 | -0.83 | -0.94 | -0.71 | -0.72 | -0.83 | -0.61 | -0.97 | -1.13 | -0.81 |
| 50 to 54 | -0.87 | -0.99 | -0.74 | -0.80 | -0.92 | -0.68 | -0.94 | -1.10 | -0.77 |
| 55 to 59 | -0.78 | -0.91 | -0.66 | -0.76 | -0.88 | -0.63 | -0.79 | -0.96 | -0.62 |
| 60 to 64 | -0.67 | -0.80 | -0.54 | -0.71 | -0.84 | -0.58 | -0.60 | -0.77 | -0.43 |
| 65 to 69 | -0.41 | -0.55 | -0.28 | -0.61 | -0.74 | -0.48 | -0.17 | -0.34 | 0.00 |
| 70 to 74 | -0.07 | -0.21 | 0.07 | -0.50 | -0.65 | -0.36 | 0.38 | 0.21 | 0.56 |
| 75 to 79 | 0.39 | 0.23 | 0.55 | -0.29 | -0.45 | -0.12 | 1.04 | 0.84 | 1.23 |
| 80 to 84 | 0.83 | 0.64 | 1.02 | -0.02 | -0.23 | 0.19 | 1.55 | 1.33 | 1.77 |
| 85 to 89 | 1.26 | 1.01 | 1.52 | 0.29 | 0.00 | 0.59 | 1.98 | 1.69 | 2.27 |
| 90 to 94 | 1.64 | 1.21 | 2.07 | 0.50 | -0.01 | 1.01 | 2.37 | 1.90 | 2.83 |
| 95 to 99 | 1.73 | 0.87 | 2.60 | 0.39 | -0.69 | 1.49 | 2.43 | 1.54 | 3.34 |

Supplementary table9: cohort effect of DALYs.

| **sex** | **Both** | | | **Male** | | | **Female** | | |
| --- | --- | --- | --- | --- | --- | --- | --- | --- | --- |
| **Cohort** | **Percent per Year** | **CILo** | **CIHi** | **Percent per Year** | **CILo** | **CIHi** | **Percent per Year** | **CILo** | **CIHi** |
| 1897~1901 | 0.86 | 0.60 | 1.23 | 1.08 | 0.66 | 1.76 | 0.75 | 0.52 | 1.09 |
| 1902~1906 | 0.89 | 0.75 | 1.05 | 1.07 | 0.86 | 1.32 | 0.79 | 0.66 | 0.94 |
| 1907~1911 | 0.95 | 0.86 | 1.04 | 1.09 | 0.98 | 1.22 | 0.86 | 0.77 | 0.95 |
| 1912~1916 | 1.00 | 0.94 | 1.06 | 1.12 | 1.04 | 1.20 | 0.92 | 0.86 | 0.99 |
| 1917~1921 | 1.06 | 1.01 | 1.11 | 1.16 | 1.10 | 1.22 | 0.98 | 0.93 | 1.04 |
| 1922~1926 | 1.09 | 1.05 | 1.13 | 1.17 | 1.12 | 1.21 | 1.03 | 0.99 | 1.08 |
| 1927~1931 | 1.10 | 1.06 | 1.13 | 1.14 | 1.10 | 1.18 | 1.06 | 1.02 | 1.10 |
| 1932~1936 | 1.12 | 1.09 | 1.15 | 1.13 | 1.10 | 1.17 | 1.09 | 1.06 | 1.13 |
| 1937~1941 | 1.11 | 1.08 | 1.14 | 1.12 | 1.09 | 1.15 | 1.10 | 1.07 | 1.14 |
| 1942~1946 | 1.10 | 1.08 | 1.13 | 1.10 | 1.08 | 1.13 | 1.10 | 1.07 | 1.13 |
| 1947~1951 | 1.06 | 1.04 | 1.08 | 1.05 | 1.03 | 1.08 | 1.06 | 1.03 | 1.09 |
| 1952~1956 | 1.03 | 1.01 | 1.05 | 1.03 | 1.01 | 1.04 | 1.03 | 1.00 | 1.05 |
| 1957~1961 | 1.00 | 1.00 | 1.00 | 1.00 | 1.00 | 1.00 | 1.00 | 1.00 | 1.00 |
| 1962~1966 | 0.96 | 0.94 | 0.98 | 0.96 | 0.94 | 0.98 | 0.96 | 0.94 | 0.98 |
| 1967~1971 | 0.91 | 0.90 | 0.93 | 0.92 | 0.90 | 0.93 | 0.91 | 0.89 | 0.93 |
| 1972~1976 | 0.89 | 0.87 | 0.90 | 0.90 | 0.88 | 0.91 | 0.87 | 0.85 | 0.89 |
| 1977~1981 | 0.87 | 0.86 | 0.89 | 0.89 | 0.88 | 0.91 | 0.85 | 0.83 | 0.87 |
| 1982~1986 | 0.85 | 0.84 | 0.87 | 0.87 | 0.85 | 0.89 | 0.83 | 0.81 | 0.85 |
| 1987~1991 | 0.82 | 0.81 | 0.84 | 0.84 | 0.82 | 0.86 | 0.80 | 0.78 | 0.82 |
| 1992~1996 | 0.81 | 0.79 | 0.82 | 0.82 | 0.81 | 0.84 | 0.79 | 0.77 | 0.81 |
| 1997~2001 | 0.78 | 0.77 | 0.80 | 0.80 | 0.78 | 0.82 | 0.76 | 0.74 | 0.78 |
| 2002~2006 | 0.75 | 0.74 | 0.77 | 0.78 | 0.76 | 0.79 | 0.72 | 0.70 | 0.74 |
| 2007~2011 | 0.71 | 0.69 | 0.72 | 0.73 | 0.72 | 0.75 | 0.68 | 0.66 | 0.70 |
| 2012~2017 | 0.65 | 0.64 | 0.67 | 0.69 | 0.67 | 0.70 | 0.62 | 0.60 | 0.64 |
| 2017~2021 | 0.60 | 0.59 | 0.62 | 0.65 | 0.63 | 0.67 | 0.55 | 0.53 | 0.56 |

Supplementary table10: cohort effect of mortality.

| **sex** | **Both** | | | **Male** | | | **Female** | | |
| --- | --- | --- | --- | --- | --- | --- | --- | --- | --- |
| **Cohort** | **Percent per Year** | **CILo** | **CIHi** | **Percent per Year** | **CILo** | **CIHi** | **Percent per Year** | **CILo** | **CIHi** |
| 1897~1901 | 0.74 | 0.56 | 0.98 | 1.15 | 0.80 | 1.65 | 0.56 | 0.42 | 0.75 |
| 1902~1906 | 0.76 | 0.66 | 0.88 | 1.08 | 0.91 | 1.28 | 0.60 | 0.51 | 0.69 |
| 1907~1911 | 0.85 | 0.79 | 0.93 | 1.10 | 1.00 | 1.21 | 0.70 | 0.64 | 0.77 |
| 1912~1916 | 0.94 | 0.88 | 1.00 | 1.14 | 1.07 | 1.22 | 0.80 | 0.74 | 0.86 |
| 1917~1921 | 1.03 | 0.98 | 1.09 | 1.21 | 1.14 | 1.27 | 0.89 | 0.84 | 0.95 |
| 1922~1926 | 1.10 | 1.06 | 1.15 | 1.22 | 1.17 | 1.28 | 1.00 | 0.95 | 1.05 |
| 1927~1931 | 1.13 | 1.09 | 1.18 | 1.19 | 1.14 | 1.24 | 1.07 | 1.02 | 1.12 |
| 1932~1936 | 1.17 | 1.13 | 1.21 | 1.18 | 1.14 | 1.22 | 1.14 | 1.09 | 1.20 |
| 1937~1941 | 1.16 | 1.12 | 1.19 | 1.15 | 1.12 | 1.19 | 1.16 | 1.11 | 1.21 |
| 1942~1946 | 1.14 | 1.11 | 1.18 | 1.13 | 1.10 | 1.17 | 1.15 | 1.11 | 1.20 |
| 1947~1951 | 1.07 | 1.04 | 1.10 | 1.06 | 1.03 | 1.09 | 1.09 | 1.05 | 1.13 |
| 1952~1956 | 1.03 | 1.00 | 1.06 | 1.03 | 1.00 | 1.05 | 1.04 | 1.00 | 1.08 |
| 1957~1961 | 1.00 | 1.00 | 1.00 | 1.00 | 1.00 | 1.00 | 1.00 | 1.00 | 1.00 |
| 1962~1966 | 0.96 | 0.94 | 0.98 | 0.96 | 0.94 | 0.98 | 0.96 | 0.93 | 1.00 |
| 1967~1971 | 0.91 | 0.88 | 0.93 | 0.91 | 0.89 | 0.94 | 0.90 | 0.87 | 0.93 |
| 1972~1976 | 0.87 | 0.85 | 0.90 | 0.89 | 0.87 | 0.91 | 0.85 | 0.82 | 0.88 |
| 1977~1981 | 0.86 | 0.84 | 0.88 | 0.88 | 0.86 | 0.91 | 0.82 | 0.79 | 0.85 |
| 1982~1986 | 0.84 | 0.82 | 0.86 | 0.86 | 0.84 | 0.89 | 0.81 | 0.78 | 0.84 |
| 1987~1991 | 0.81 | 0.79 | 0.84 | 0.83 | 0.81 | 0.86 | 0.78 | 0.75 | 0.82 |
| 1992~1996 | 0.79 | 0.76 | 0.81 | 0.80 | 0.78 | 0.83 | 0.76 | 0.73 | 0.80 |
| 1997~2001 | 0.75 | 0.73 | 0.78 | 0.77 | 0.75 | 0.80 | 0.72 | 0.69 | 0.75 |
| 2002~2006 | 0.71 | 0.68 | 0.73 | 0.75 | 0.72 | 0.77 | 0.66 | 0.63 | 0.69 |
| 2007~2011 | 0.65 | 0.62 | 0.68 | 0.69 | 0.66 | 0.72 | 0.59 | 0.56 | 0.63 |
| 2012~2017 | 0.59 | 0.56 | 0.61 | 0.64 | 0.61 | 0.67 | 0.52 | 0.49 | 0.55 |
| 2017~2021 | 0.54 | 0.51 | 0.57 | 0.62 | 0.59 | 0.65 | 0.45 | 0.42 | 0.48 |

Supplementary table11: cohort effect of prevalence.

| **sex** | **Both** | | | **Male** | | | **Female** | | |
| --- | --- | --- | --- | --- | --- | --- | --- | --- | --- |
| **Cohort** | **Percent per Year** | **CILo** | **CIHi** | **Percent per Year** | **CILo** | **CIHi** | **Percent per Year** | **CILo** | **CIHi** |
| 1897~1901 | 0.68 | 0.55 | 0.84 | 0.73 | 0.56 | 0.96 | 0.66 | 0.53 | 0.81 |
| 1902~1906 | 0.72 | 0.65 | 0.80 | 0.77 | 0.69 | 0.86 | 0.70 | 0.63 | 0.77 |
| 1907~1911 | 0.77 | 0.73 | 0.81 | 0.81 | 0.76 | 0.86 | 0.75 | 0.70 | 0.79 |
| 1912~1916 | 0.81 | 0.78 | 0.85 | 0.85 | 0.82 | 0.89 | 0.79 | 0.76 | 0.83 |
| 1917~1921 | 0.85 | 0.82 | 0.87 | 0.88 | 0.85 | 0.90 | 0.83 | 0.80 | 0.86 |
| 1922~1926 | 0.89 | 0.87 | 0.91 | 0.91 | 0.89 | 0.94 | 0.88 | 0.85 | 0.90 |
| 1927~1931 | 0.91 | 0.89 | 0.93 | 0.93 | 0.91 | 0.95 | 0.90 | 0.88 | 0.92 |
| 1932~1936 | 0.94 | 0.92 | 0.95 | 0.95 | 0.93 | 0.96 | 0.93 | 0.90 | 0.95 |
| 1937~1941 | 0.96 | 0.94 | 0.98 | 0.97 | 0.95 | 0.99 | 0.95 | 0.93 | 0.97 |
| 1942~1946 | 0.98 | 0.97 | 1.00 | 0.98 | 0.97 | 1.00 | 0.98 | 0.96 | 1.00 |
| 1947~1951 | 0.99 | 0.98 | 1.01 | 0.99 | 0.98 | 1.01 | 0.99 | 0.97 | 1.01 |
| 1952~1956 | 0.99 | 0.98 | 1.01 | 1.00 | 0.98 | 1.01 | 0.99 | 0.98 | 1.01 |
| 1957~1961 | 1.00 | 1.00 | 1.00 | 1.00 | 1.00 | 1.00 | 1.00 | 1.00 | 1.00 |
| 1962~1966 | 0.99 | 0.97 | 1.00 | 0.99 | 0.98 | 1.00 | 0.99 | 0.97 | 1.00 |
| 1967~1971 | 0.96 | 0.95 | 0.98 | 0.96 | 0.95 | 0.98 | 0.96 | 0.95 | 0.98 |
| 1972~1976 | 0.96 | 0.95 | 0.97 | 0.96 | 0.95 | 0.98 | 0.96 | 0.94 | 0.97 |
| 1977~1981 | 0.97 | 0.96 | 0.98 | 0.98 | 0.97 | 0.99 | 0.96 | 0.94 | 0.97 |
| 1982~1986 | 0.97 | 0.95 | 0.98 | 0.98 | 0.97 | 0.99 | 0.95 | 0.94 | 0.97 |
| 1987~1991 | 0.95 | 0.94 | 0.97 | 0.97 | 0.96 | 0.98 | 0.93 | 0.92 | 0.95 |
| 1992~1996 | 0.96 | 0.94 | 0.97 | 0.98 | 0.96 | 0.99 | 0.94 | 0.92 | 0.96 |
| 1997~2001 | 0.96 | 0.94 | 0.97 | 0.98 | 0.96 | 0.99 | 0.94 | 0.92 | 0.96 |
| 2002~2006 | 0.96 | 0.94 | 0.97 | 0.98 | 0.96 | 0.99 | 0.94 | 0.92 | 0.96 |
| 2007~2011 | 0.95 | 0.93 | 0.97 | 0.96 | 0.95 | 0.98 | 0.94 | 0.92 | 0.96 |
| 2012~2017 | 0.93 | 0.91 | 0.94 | 0.94 | 0.92 | 0.96 | 0.91 | 0.89 | 0.94 |
| 2017~2021 | 0.89 | 0.87 | 0.91 | 0.90 | 0.88 | 0.92 | 0.88 | 0.86 | 0.91 |

Supplementary table12: cohort effect of incidence.

| **sex** | **Both** | | | **Male** | | | **Female** | | |
| --- | --- | --- | --- | --- | --- | --- | --- | --- | --- |
| **Cohort** | **Percent per Year** | **CILo** | **CIHi** | **Percent per Year** | **CILo** | **CIHi** | **Percent per Year** | **CILo** | **CIHi** |
| 1897~1901 | 0.66 | 0.52 | 0.83 | 0.71 | 0.50 | 0.99 | 0.63 | 0.51 | 0.77 |
| 1902~1906 | 0.68 | 0.60 | 0.76 | 0.73 | 0.63 | 0.85 | 0.64 | 0.58 | 0.72 |
| 1907~1911 | 0.70 | 0.65 | 0.75 | 0.76 | 0.69 | 0.83 | 0.67 | 0.62 | 0.72 |
| 1912~1916 | 0.74 | 0.70 | 0.78 | 0.79 | 0.75 | 0.84 | 0.71 | 0.67 | 0.74 |
| 1917~1921 | 0.77 | 0.74 | 0.80 | 0.82 | 0.78 | 0.85 | 0.74 | 0.71 | 0.77 |
| 1922~1926 | 0.82 | 0.80 | 0.85 | 0.86 | 0.83 | 0.89 | 0.79 | 0.76 | 0.82 |
| 1927~1931 | 0.85 | 0.83 | 0.87 | 0.88 | 0.86 | 0.91 | 0.82 | 0.80 | 0.85 |
| 1932~1936 | 0.88 | 0.86 | 0.90 | 0.90 | 0.88 | 0.93 | 0.86 | 0.84 | 0.88 |
| 1937~1941 | 0.92 | 0.90 | 0.94 | 0.93 | 0.91 | 0.95 | 0.90 | 0.88 | 0.92 |
| 1942~1946 | 0.95 | 0.93 | 0.97 | 0.96 | 0.94 | 0.97 | 0.94 | 0.92 | 0.96 |
| 1947~1951 | 0.97 | 0.95 | 0.99 | 0.97 | 0.95 | 0.99 | 0.97 | 0.95 | 0.98 |
| 1952~1956 | 0.98 | 0.97 | 0.99 | 0.98 | 0.97 | 1.00 | 0.98 | 0.96 | 0.99 |
| 1957~1961 | 1.00 | 1.00 | 1.00 | 1.00 | 1.00 | 1.00 | 1.00 | 1.00 | 1.00 |
| 1962~1966 | 1.00 | 0.99 | 1.02 | 1.00 | 0.99 | 1.02 | 1.01 | 0.99 | 1.02 |
| 1967~1971 | 0.99 | 0.98 | 1.00 | 0.98 | 0.97 | 1.00 | 1.00 | 0.98 | 1.01 |
| 1972~1976 | 1.00 | 0.98 | 1.01 | 0.99 | 0.98 | 1.01 | 1.00 | 0.99 | 1.02 |
| 1977~1981 | 1.02 | 1.00 | 1.03 | 1.02 | 1.01 | 1.04 | 1.02 | 1.00 | 1.03 |
| 1982~1986 | 1.03 | 1.02 | 1.05 | 1.04 | 1.02 | 1.05 | 1.02 | 1.01 | 1.04 |
| 1987~1991 | 1.03 | 1.01 | 1.04 | 1.03 | 1.02 | 1.05 | 1.02 | 1.00 | 1.03 |
| 1992~1996 | 1.04 | 1.03 | 1.06 | 1.05 | 1.04 | 1.07 | 1.03 | 1.01 | 1.05 |
| 1997~2001 | 1.06 | 1.04 | 1.08 | 1.08 | 1.06 | 1.10 | 1.04 | 1.02 | 1.06 |
| 2002~2006 | 1.08 | 1.06 | 1.09 | 1.09 | 1.08 | 1.11 | 1.06 | 1.04 | 1.07 |
| 2007~2011 | 1.09 | 1.07 | 1.10 | 1.10 | 1.08 | 1.12 | 1.07 | 1.05 | 1.09 |
| 2012~2017 | 1.08 | 1.06 | 1.10 | 1.09 | 1.07 | 1.11 | 1.06 | 1.04 | 1.08 |
| 2017~2021 | 1.06 | 1.04 | 1.08 | 1.07 | 1.05 | 1.10 | 1.04 | 1.02 | 1.06 |

Supplementary table13: period effect of DALYs and Prevalence.

| Dalys | | | | | | | | | | Prevalence | | | | | | | | |
| --- | --- | --- | --- | --- | --- | --- | --- | --- | --- | --- | --- | --- | --- | --- | --- | --- | --- | --- |
| **sex** | **Both** | | | **Male** | | | **Female** | | | **Both** | | | **Male** | | | **Female** | | |
| **Period** | **Percent per Year** | **CILo** | **CIHi** | **Percent per Year** | **CILo** | **CIHi** | **Percent per Year** | **CILo** | **CIHi** | **Percent per Year** | **CILo** | **CIHi** | **Percent per Year** | **CILo** | **CIHi** | **Percent per Year** | **CILo** | **CIHi** |
| 1992-1996 | 1.04 | 1.03 | 1.05 | 1.05 | 1.04 | 1.06 | 1.04 | 1.03 | 1.05 | 0.97 | 0.96 | 0.98 | 0.97 | 0.96 | 0.98 | 0.97 | 0.96 | 0.98 |
| 1997-2001 | 1.02 | 1.02 | 1.03 | 1.02 | 1.01 | 1.03 | 1.03 | 1.02 | 1.04 | 0.99 | 0.98 | 1.00 | 0.99 | 0.98 | 1.00 | 0.99 | 0.99 | 1.00 |
| 2002-2006 | 1.00 | 1.00 | 1.00 | 1.00 | 1.00 | 1.00 | 1.00 | 1.00 | 1.00 | 1.00 | 1.00 | 1.00 | 1.00 | 1.00 | 1.00 | 1.00 | 1.00 | 1.00 |
| 2007-2011 | 0.97 | 0.96 | 0.98 | 0.96 | 0.96 | 0.97 | 0.97 | 0.96 | 0.98 | 1.01 | 1.00 | 1.01 | 1.01 | 1.00 | 1.01 | 1.00 | 1.00 | 1.01 |
| 2012-2016 | 0.96 | 0.95 | 0.97 | 0.95 | 0.94 | 0.96 | 0.96 | 0.95 | 0.97 | 1.00 | 1.00 | 1.01 | 1.00 | 0.99 | 1.01 | 1.00 | 1.00 | 1.01 |
| 2017-2021 | 0.95 | 0.94 | 0.96 | 0.94 | 0.93 | 0.95 | 0.97 | 0.95 | 0.98 | 1.01 | 1.00 | 1.02 | 1.00 | 1.00 | 1.01 | 1.01 | 1.00 | 1.02 |

Supplementary table14: period effect of Incidence and Motality.

| Incidence | | | | | | | | | | Motality | | | | | | | | |
| --- | --- | --- | --- | --- | --- | --- | --- | --- | --- | --- | --- | --- | --- | --- | --- | --- | --- | --- |
| **sex** | **Both** | | | **Male** | | | **Female** | | | **Both** | | | **Male** | | | **Female** | | |
| **Period** | **Percent per Year** | **CILo** | **CIHi** | **Percent per Year** | **CILo** | **CIHi** | **Percent per Year** | **CILo** | **CIHi** | **Percent per Year** | **CILo** | **CIHi** | **Percent per Year** | **CILo** | **CIHi** | **Percent per Year** | **CILo** | **CIHi** |
| 1992-1996 | 0.96 | 0.95 | 0.97 | 0.96 | 0.96 | 0.97 | 0.96 | 0.95 | 0.97 | 1.06 | 1.04 | 1.07 | 1.06 | 1.05 | 1.08 | 1.06 | 1.04 | 1.08 |
| 1997-2001 | 0.99 | 0.98 | 1.00 | 0.99 | 0.98 | 1.00 | 0.99 | 0.98 | 1.00 | 1.02 | 1.01 | 1.04 | 1.02 | 1.01 | 1.04 | 1.03 | 1.01 | 1.05 |
| 2002-2006 | 1.00 | 1.00 | 1.00 | 1.00 | 1.00 | 1.00 | 1.00 | 1.00 | 1.00 | 1.00 | 1.00 | 1.00 | 1.00 | 1.00 | 1.00 | 1.00 | 1.00 | 1.00 |
| 2007-2011 | 1.02 | 1.01 | 1.03 | 1.01 | 1.01 | 1.02 | 1.02 | 1.01 | 1.03 | 0.95 | 0.94 | 0.97 | 0.95 | 0.94 | 0.96 | 0.95 | 0.93 | 0.97 |
| 2012-2016 | 1.04 | 1.03 | 1.05 | 1.03 | 1.02 | 1.04 | 1.04 | 1.04 | 1.05 | 0.96 | 0.94 | 0.97 | 0.94 | 0.93 | 0.96 | 0.96 | 0.95 | 0.98 |
| 2017-2021 | 1.06 | 1.05 | 1.07 | 1.05 | 1.04 | 1.06 | 1.07 | 1.06 | 1.08 | 0.96 | 0.95 | 0.98 | 0.93 | 0.92 | 0.95 | 0.98 | 0.96 | 1.00 |

Supplementary table15: Projection of age-standardized DALYs rate.

| **year** | Male | | | Female | | | Both | | |
| --- | --- | --- | --- | --- | --- | --- | --- | --- | --- |
|  | **ASR** | **upper** | **lower** | **ASR** | **upper** | **lower** | **ASR** | **upper** | **lower** |
| 1990 | 238.2615002 | 238.4524095 | 238.070591 | 187.3860811 | 187.5528337 | 187.2193286 | 212.5930571 | 212.7190154 | 212.4670989 |
| 1991 | 238.8243103 | 239.0136468 | 238.6349738 | 187.5363201 | 187.7018702 | 187.37077 | 212.9639557 | 213.0889965 | 212.838915 |
| 1992 | 239.1654637 | 239.3530567 | 238.9778708 | 188.4455787 | 188.6100915 | 188.281066 | 213.5938787 | 213.717959 | 213.4697983 |
| 1993 | 239.3075467 | 239.4934217 | 239.1216717 | 188.8348935 | 188.9981884 | 188.6715987 | 213.8606816 | 213.9837392 | 213.7376241 |
| 1994 | 238.6306028 | 238.8144863 | 238.4467192 | 188.1662127 | 188.3278129 | 188.0046125 | 213.1908891 | 213.3126594 | 213.0691188 |
| 1995 | 238.2600241 | 238.4422497 | 238.0777984 | 187.5236525 | 187.6837848 | 187.3635203 | 212.693757 | 212.8144462 | 212.5730679 |
| 1996 | 236.6609298 | 236.8409769 | 236.4808827 | 186.2520865 | 186.4104043 | 186.0937687 | 211.2670669 | 211.3863598 | 211.1477741 |
| 1997 | 234.560563 | 234.7384225 | 234.3827035 | 184.9507365 | 185.1073711 | 184.794102 | 209.5672297 | 209.6851616 | 209.4492979 |
| 1998 | 231.8447851 | 232.0202892 | 231.6692809 | 183.2417274 | 183.3965533 | 183.0869016 | 207.3615054 | 207.4779803 | 207.2450306 |
| 1999 | 230.7140426 | 230.88787 | 230.5402152 | 182.0023744 | 182.1556641 | 181.8490847 | 206.1842334 | 206.2995923 | 206.0688745 |
| 2000 | 229.4347244 | 229.606754 | 229.2626949 | 180.7453397 | 180.8970842 | 180.5935951 | 204.9381851 | 205.0523922 | 204.823978 |
| 2001 | 228.2954673 | 228.4658623 | 228.1250722 | 179.3310086 | 179.4812487 | 179.1807686 | 203.6757393 | 203.788863 | 203.5626156 |
| 2002 | 227.0270049 | 227.1957714 | 226.8582385 | 177.7201105 | 177.8687709 | 177.5714502 | 202.2512457 | 202.3632628 | 202.1392286 |
| 2003 | 227.2539808 | 227.4216596 | 227.086302 | 176.6064579 | 176.75378 | 176.4591359 | 201.8318157 | 201.9430107 | 201.7206207 |
| 2004 | 225.2606497 | 225.4265559 | 225.0947434 | 174.9920959 | 175.1379079 | 174.8462839 | 200.0183599 | 200.1283975 | 199.9083224 |
| 2005 | 224.4396238 | 224.6040504 | 224.2751972 | 173.693381 | 173.8378205 | 173.5489415 | 198.9808439 | 199.0899085 | 198.8717793 |
| 2006 | 221.7029053 | 221.8651884 | 221.5406222 | 171.4882213 | 171.6308868 | 171.3455559 | 196.5251857 | 196.6328903 | 196.4174812 |
| 2007 | 220.1417069 | 220.30222 | 219.9811937 | 169.407073 | 169.5479559 | 169.2661901 | 194.721539 | 194.8280195 | 194.6150584 |
| 2008 | 217.8091647 | 217.9676363 | 217.650693 | 167.8312031 | 167.9704834 | 167.6919229 | 192.7771982 | 192.8824069 | 192.6719896 |
| 2009 | 215.3700315 | 215.5264154 | 215.2136477 | 166.3795988 | 166.5173252 | 166.2418723 | 190.836505 | 190.9404382 | 190.7325718 |
| 2010 | 213.6333959 | 213.7880484 | 213.4787434 | 164.5569395 | 164.6930295 | 164.4208495 | 189.0621369 | 189.1649004 | 188.9593735 |
| 2011 | 211.374221 | 211.5270378 | 211.2214043 | 162.7075172 | 162.842023 | 162.5730114 | 187.0104119 | 187.1119808 | 186.9088429 |
| 2012 | 209.7940308 | 209.9452742 | 209.6427875 | 161.5490191 | 161.682225 | 161.4158133 | 185.6429914 | 185.7435556 | 185.5424272 |
| 2013 | 210.6165455 | 210.7670857 | 210.4660053 | 161.2684422 | 161.4007499 | 161.1361344 | 185.9484479 | 186.0484874 | 185.8484084 |
| 2014 | 210.0933004 | 210.2426917 | 209.9439091 | 160.5442352 | 160.6754862 | 160.4129841 | 185.3310263 | 185.4302997 | 185.2317528 |
| 2015 | 209.7399641 | 209.8883321 | 209.5915961 | 159.8478174 | 159.9780444 | 159.7175904 | 184.8193485 | 184.9179161 | 184.7207809 |
| 2016 | 209.3790081 | 209.526331 | 209.2316852 | 159.6958066 | 159.8252372 | 159.566376 | 184.5863445 | 184.6842808 | 184.4884083 |
| 2017 | 208.3396764 | 208.4857546 | 208.1935983 | 158.8135971 | 158.9419625 | 158.6852316 | 183.6467523 | 183.743892 | 183.5496125 |
| 2018 | 205.0285747 | 205.172675 | 204.8844743 | 156.1118214 | 156.2384409 | 155.985202 | 180.6473127 | 180.7431483 | 180.5514772 |
| 2019 | 203.6919084 | 203.8348082 | 203.5490086 | 154.7834684 | 154.9089959 | 154.6579408 | 179.3237787 | 179.4188175 | 179.22874 |
| 2020 | 201.5445925 | 201.6861277 | 201.4030573 | 153.3557777 | 153.4801653 | 153.23139 | 177.5431306 | 177.63729 | 177.4489712 |
| 2021 | 201.0720327 | 201.2129285 | 200.9311369 | 154.0420498 | 154.1663403 | 153.9177593 | 177.6495472 | 177.7434318 | 177.5556626 |
| 2022 | 201.4626396 | 208.8567371 | 194.0685422 | 153.3132344 | 159.4825112 | 147.1439576 | 177.5581116 | 184.2807049 | 170.8355183 |
| 2023 | 200.0785381 | 209.3383965 | 190.8186797 | 152.0237242 | 159.5748032 | 144.4726452 | 176.2115683 | 184.5678927 | 167.8552439 |
| 2024 | 198.6306014 | 210.3465693 | 186.9146334 | 150.6497937 | 160.0255063 | 141.2740811 | 174.7878909 | 185.299543 | 164.2762387 |
| 2025 | 197.1200908 | 211.7440938 | 182.4960878 | 149.1891669 | 160.7289063 | 137.6494275 | 173.2866576 | 186.353577 | 160.2197382 |
| 2026 | 195.5531449 | 213.4428168 | 177.663473 | 147.6501071 | 161.6176655 | 133.6825487 | 171.7162148 | 187.6530121 | 155.7794175 |
| 2027 | 193.9412713 | 215.3902152 | 172.4923274 | 146.0369836 | 162.640995 | 129.4329722 | 170.0843128 | 189.1452699 | 151.0233557 |
| 2028 | 192.3021786 | 217.5479512 | 167.0564061 | 144.369369 | 163.7704391 | 124.968299 | 168.408927 | 190.7950721 | 146.022782 |
| 2029 | 190.6524417 | 219.8970595 | 161.4078238 | 142.6657192 | 164.9932482 | 120.3381901 | 166.7071994 | 192.5850287 | 140.8293701 |
| 2030 | 189.0094693 | 222.4340279 | 155.5849108 | 140.9428183 | 166.3064653 | 115.5791714 | 164.9962107 | 194.5109267 | 135.4814948 |
| 2031 | 187.3858155 | 225.1589042 | 149.6127269 | 139.2207294 | 167.7182361 | 110.7232226 | 163.2931145 | 196.577112 | 130.009117 |
| 2032 | 185.7888721 | 228.0682948 | 143.5094494 | 137.5070147 | 169.2245027 | 105.7895268 | 161.6051879 | 198.7790497 | 124.4313262 |
| 2033 | 184.2272473 | 231.1535107 | 137.300984 | 135.8160116 | 170.8248539 | 100.8071693 | 159.9434803 | 201.1110707 | 118.77589 |
| 2034 | 182.7065453 | 234.4094384 | 131.0036522 | 134.1560497 | 172.5181716 | 95.7939278 | 158.3149043 | 203.5694406 | 113.0603679 |
| 2035 | 181.2273962 | 237.8305255 | 124.624267 | 132.5262307 | 174.2965108 | 90.75595067 | 156.7193994 | 206.1469995 | 107.2917994 |
| 2036 | 179.7756731 | 241.3952009 | 118.1561453 | 130.9164623 | 176.142081 | 85.69084363 | 155.1454022 | 208.8243139 | 101.4664904 |
| 2037 | 178.3339793 | 245.0756942 | 111.5922643 | 129.3091892 | 178.0256134 | 80.59276507 | 153.5752236 | 211.5721406 | 95.57830662 |
| 2038 | 176.8865165 | 248.837792 | 104.935241 | 127.6936773 | 179.9202369 | 75.46711763 | 151.9951856 | 214.3588303 | 89.63154079 |
| 2039 | 175.4218004 | 252.6551776 | 98.18842329 | 126.0618558 | 181.8046977 | 70.31901391 | 150.3952981 | 217.1597092 | 83.63088702 |
| 2040 | 173.9352901 | 256.5133871 | 91.35719309 | 124.4063815 | 183.6604082 | 65.15235486 | 148.7695749 | 219.9575896 | 77.58156022 |
| 2041 | 172.4223178 | 260.3992995 | 84.44533602 | 122.7239382 | 185.4753336 | 59.97254271 | 147.114418 | 222.7398855 | 71.48895043 |
| 2042 | 170.8817691 | 264.3040942 | 77.4594441 | 121.0094259 | 187.233945 | 54.78490678 | 145.4267279 | 225.4938896 | 65.3595661 |
| 2043 | 169.3183618 | 268.2223856 | 70.41433802 | 119.2692127 | 188.9336236 | 49.6048017 | 143.7119533 | 228.214885 | 59.20902161 |
| 2044 | 167.7412203 | 272.1587185 | 63.32372216 | 117.5127207 | 190.5793928 | 44.44604861 | 141.9795223 | 230.9073364 | 53.05170821 |
| 2045 | 166.160034 | 276.1220153 | 56.19805265 | 115.7476189 | 192.1765155 | 39.31872226 | 140.2382289 | 233.5779903 | 46.89846757 |
| 2046 | 164.5789846 | 280.1150601 | 49.04290899 | 113.9806263 | 193.7308762 | 34.23037648 | 138.4938469 | 236.2311953 | 40.75649854 |
| 2047 | 162.998304 | 284.1342808 | 41.86232716 | 112.2106855 | 195.23597 | 29.18540091 | 136.7460064 | 238.8614883 | 34.63052451 |
| 2048 | 161.4198395 | 288.1747345 | 34.6649446 | 110.4412629 | 196.689699 | 24.19282687 | 134.9974965 | 241.4650603 | 28.52993262 |
| 2049 | 159.8453724 | 292.2334906 | 27.45725423 | 108.6766238 | 198.0934371 | 19.25981055 | 133.2514352 | 244.0407947 | 22.46207584 |
| 2050 | 158.2779404 | 296.3118515 | 20.24402937 | 106.9192529 | 199.4474513 | 14.39105437 | 131.5106254 | 246.5890689 | 16.43218202 |

Supplementary table16: Projection of ASDR.

| **year** | Male | | | Female | | | Both | | |
| --- | --- | --- | --- | --- | --- | --- | --- | --- | --- |
|  | **ASR** | **upper** | **lower** | **ASR** | **upper** | **lower** | **ASR** | **upper** | **lower** |
| 1990 | 2.556504061 | 2.577132431 | 2.535875691 | 1.620523509 | 1.635975398 | 1.605071619 | 2.066775931 | 2.079522618 | 2.054029244 |
| 1991 | 2.543669881 | 2.563644398 | 2.523695363 | 1.608993336 | 1.624040986 | 1.593945687 | 2.054788645 | 2.067265748 | 2.042311542 |
| 1992 | 2.537897112 | 2.557562247 | 2.518231977 | 1.614967981 | 1.629871924 | 1.600064038 | 2.055404974 | 2.067746621 | 2.043063327 |
| 1993 | 2.536801904 | 2.556260476 | 2.517343333 | 1.617425524 | 1.63221863 | 1.602632419 | 2.056344195 | 2.068576208 | 2.044112182 |
| 1994 | 2.53323442 | 2.55251649 | 2.513952349 | 1.613390403 | 1.628058842 | 1.598721964 | 2.052959652 | 2.065086551 | 2.040832752 |
| 1995 | 2.536284786 | 2.555426704 | 2.517142868 | 1.612745832 | 1.627319986 | 1.598171679 | 2.055359119 | 2.067408599 | 2.043309639 |
| 1996 | 2.517331185 | 2.536231963 | 2.498430407 | 1.603955852 | 1.618382526 | 1.589529177 | 2.041794384 | 2.053705456 | 2.029883312 |
| 1997 | 2.487539804 | 2.506162249 | 2.468917359 | 1.591078322 | 1.605336882 | 1.576819763 | 2.020174597 | 2.031921867 | 2.008427326 |
| 1998 | 2.454362347 | 2.4727018 | 2.436022894 | 1.576948008 | 1.591041109 | 1.562854906 | 1.996763582 | 2.008350919 | 1.985176244 |
| 1999 | 2.446240403 | 2.464404187 | 2.428076618 | 1.567554507 | 1.581504236 | 1.553604778 | 1.988441397 | 1.99991528 | 1.976967514 |
| 2000 | 2.429535504 | 2.447478077 | 2.411592932 | 1.55827718 | 1.572087266 | 1.544467094 | 1.976224417 | 1.987572735 | 1.9648761 |
| 2001 | 2.420478215 | 2.438241317 | 2.402715114 | 1.550143526 | 1.563826053 | 1.536460998 | 1.968505517 | 1.979747819 | 1.957263216 |
| 2002 | 2.412713168 | 2.430311358 | 2.395114978 | 1.540544214 | 1.554090026 | 1.526998403 | 1.960440569 | 1.971577298 | 1.94930384 |
| 2003 | 2.429295875 | 2.446815421 | 2.41177633 | 1.542925028 | 1.556392571 | 1.529457484 | 1.971548436 | 1.982634648 | 1.960462223 |
| 2004 | 2.413003639 | 2.430343802 | 2.395663475 | 1.530275585 | 1.543595742 | 1.516955428 | 1.957143083 | 1.968109502 | 1.946176664 |
| 2005 | 2.396511323 | 2.413616822 | 2.379405824 | 1.516637432 | 1.529802087 | 1.503472777 | 1.942911971 | 1.953743682 | 1.93208026 |
| 2006 | 2.348308289 | 2.365079586 | 2.331536992 | 1.487655577 | 1.500595242 | 1.474715913 | 1.904593622 | 1.915228331 | 1.893958912 |
| 2007 | 2.332034797 | 2.348593316 | 2.315476278 | 1.467719836 | 1.480471333 | 1.454968338 | 1.887298219 | 1.897794632 | 1.876801806 |
| 2008 | 2.304660987 | 2.320975151 | 2.288346822 | 1.456374616 | 1.468977917 | 1.443771314 | 1.868741351 | 1.87909892 | 1.858383783 |
| 2009 | 2.278829358 | 2.294903939 | 2.262754777 | 1.447752234 | 1.460219754 | 1.435284714 | 1.852089018 | 1.862312372 | 1.841865664 |
| 2010 | 2.26227124 | 2.278155103 | 2.246387377 | 1.429614194 | 1.4419086 | 1.417319787 | 1.834776622 | 1.844869935 | 1.824683309 |
| 2011 | 2.236152315 | 2.251816701 | 2.220487929 | 1.411982651 | 1.424110836 | 1.399854466 | 1.81288934 | 1.822844366 | 1.802934314 |
| 2012 | 2.228695947 | 2.244213791 | 2.213178103 | 1.410420258 | 1.422451967 | 1.398388548 | 1.80877153 | 1.81863847 | 1.798904589 |
| 2013 | 2.248873748 | 2.264326901 | 2.233420595 | 1.422096504 | 1.434093242 | 1.410099766 | 1.826547832 | 1.836387017 | 1.816708648 |
| 2014 | 2.258357806 | 2.273724322 | 2.242991289 | 1.426517279 | 1.438446746 | 1.414587812 | 1.833989753 | 1.843773776 | 1.82420573 |
| 2015 | 2.270221033 | 2.285521798 | 2.254920268 | 1.430435469 | 1.442296169 | 1.418574769 | 1.842288064 | 1.85202317 | 1.832552958 |
| 2016 | 2.273394773 | 2.288586274 | 2.258203273 | 1.44464469 | 1.456479727 | 1.432809652 | 1.852305299 | 1.861993525 | 1.842617072 |
| 2017 | 2.271672328 | 2.286736643 | 2.256608012 | 1.450678686 | 1.462450455 | 1.438906916 | 1.85569114 | 1.865314046 | 1.846068234 |
| 2018 | 2.234487963 | 2.249306259 | 2.219669666 | 1.423858399 | 1.435428196 | 1.412288602 | 1.823568612 | 1.833032926 | 1.814104299 |
| 2019 | 2.22036787 | 2.235031884 | 2.205703857 | 1.409978967 | 1.421415971 | 1.398541963 | 1.809930803 | 1.819292521 | 1.800569085 |
| 2020 | 2.186407144 | 2.200891037 | 2.17192325 | 1.386532566 | 1.397819376 | 1.375245756 | 1.781172567 | 1.790406249 | 1.771938885 |
| 2021 | 2.146386443 | 2.16080615 | 2.131966736 | 1.366716323 | 1.37796858 | 1.355464065 | 1.751092013 | 1.7602423 | 1.741941727 |
| 2022 | 2.113826791 | 2.20236669 | 2.025286893 | 1.349737072 | 1.415666211 | 1.283807934 | 1.726447807 | 1.803807705 | 1.64908791 |
| 2023 | 2.083596174 | 2.19885738 | 1.968334969 | 1.32929962 | 1.412912931 | 1.245686308 | 1.700431288 | 1.800146536 | 1.60071604 |
| 2024 | 2.053720316 | 2.2030516 | 1.904389032 | 1.308593893 | 1.414846108 | 1.202341678 | 1.674413844 | 1.80274699 | 1.546080698 |
| 2025 | 2.024204534 | 2.212843915 | 1.835565153 | 1.287621966 | 1.420040326 | 1.155203607 | 1.648402623 | 1.809790054 | 1.487015192 |
| 2026 | 1.995045575 | 2.226892889 | 1.763198261 | 1.266432498 | 1.427569375 | 1.10529562 | 1.622445465 | 1.820128566 | 1.424762364 |
| 2027 | 1.966301851 | 2.244378839 | 1.688224864 | 1.245036668 | 1.436756545 | 1.053316792 | 1.596573754 | 1.832984223 | 1.360163286 |
| 2028 | 1.93786097 | 2.264449686 | 1.611272253 | 1.223384814 | 1.44697542 | 0.999794208 | 1.570697605 | 1.847591732 | 1.293803477 |
| 2029 | 1.909612754 | 2.286486711 | 1.532738796 | 1.201459268 | 1.457802671 | 0.945115865 | 1.54475122 | 1.863413698 | 1.226088742 |
| 2030 | 1.881534859 | 2.310125719 | 1.452943998 | 1.179295338 | 1.468987245 | 0.88960343 | 1.518742056 | 1.880130728 | 1.157353385 |
| 2031 | 1.85362635 | 2.335121224 | 1.372131477 | 1.156997678 | 1.480430569 | 0.833564788 | 1.492740405 | 1.897582278 | 1.087898533 |
| 2032 | 1.82593442 | 2.36132057 | 1.290548271 | 1.134635763 | 1.492022597 | 0.77724893 | 1.466799493 | 1.91562213 | 1.017976857 |
| 2033 | 1.798371169 | 2.388336281 | 1.208406057 | 1.112240688 | 1.503580493 | 0.720900884 | 1.440881058 | 1.933945368 | 0.947816748 |
| 2034 | 1.770861536 | 2.415852561 | 1.12587051 | 1.089860524 | 1.514997794 | 0.664723253 | 1.414972038 | 1.95233193 | 0.877612146 |
| 2035 | 1.743459627 | 2.443777453 | 1.043141801 | 1.06758701 | 1.526272974 | 0.608901046 | 1.389145339 | 1.970725084 | 0.807565594 |
| 2036 | 1.716279334 | 2.472148352 | 0.960410316 | 1.045564161 | 1.537514774 | 0.553613548 | 1.363546417 | 1.989212015 | 0.737880819 |
| 2037 | 1.689446655 | 2.501041091 | 0.877852219 | 1.023852302 | 1.548730174 | 0.49897443 | 1.338264528 | 2.007821961 | 0.668707095 |
| 2038 | 1.662976788 | 2.53032452 | 0.795629057 | 1.002443875 | 1.559798901 | 0.44508885 | 1.313295466 | 2.026411912 | 0.600179021 |
| 2039 | 1.636859886 | 2.559871027 | 0.713848745 | 0.98131102 | 1.570596832 | 0.392025207 | 1.288619723 | 2.044847623 | 0.532391823 |
| 2040 | 1.611160043 | 2.589705489 | 0.632614597 | 0.960453282 | 1.581064105 | 0.339842459 | 1.264268062 | 2.063102313 | 0.465433812 |
| 2041 | 1.58594828 | 2.61990074 | 0.55199582 | 0.939923021 | 1.591244208 | 0.288601835 | 1.240313247 | 2.081244424 | 0.39938207 |
| 2042 | 1.561272891 | 2.650505379 | 0.472040404 | 0.91971348 | 1.601088343 | 0.238338616 | 1.216774797 | 2.099265929 | 0.334283665 |
| 2043 | 1.537078886 | 2.681351923 | 0.39280585 | 0.899777311 | 1.61044809 | 0.189106532 | 1.193596078 | 2.11699411 | 0.270198045 |
| 2044 | 1.513292231 | 2.712257902 | 0.31432656 | 0.880083201 | 1.619215491 | 0.140950911 | 1.17072611 | 2.134279401 | 0.207172819 |
| 2045 | 1.489915278 | 2.743193129 | 0.236637427 | 0.860655615 | 1.627398605 | 0.093912625 | 1.148176008 | 2.151099237 | 0.145252779 |
| 2046 | 1.466958542 | 2.774163073 | 0.159754011 | 0.841588639 | 1.635155759 | 0.048021519 | 1.126005154 | 2.167540464 | 0.084469844 |
| 2047 | 1.444440296 | 2.80519089 | 0.083689701 | 0.822903632 | 1.642520621 | 0.003286643 | 1.104232082 | 2.183622757 | 0.024841407 |
| 2048 | 1.422290107 | 2.836091759 | 0.008488454 | 0.804553164 | 1.649370318 | -0.04026399 | 1.082797754 | 2.199186111 | -0.033590604 |
| 2049 | 1.400451573 | 2.866715719 | -0.065812573 | 0.786498005 | 1.655604283 | -0.082608272 | 1.061657637 | 2.214105397 | -0.090790124 |
| 2050 | 1.378931915 | 2.89705265 | -0.139188821 | 0.768742132 | 1.661219722 | -0.123735458 | 1.040816751 | 2.228367092 | -0.14673359 |

Supplementary table17: Projection of ASIR.

| **year** | Male | | | Female | | | Both | | |
| --- | --- | --- | --- | --- | --- | --- | --- | --- | --- |
|  | **ASR** | **upper** | **lower** | **ASR** | **upper** | **lower** | **ASR** | **upper** | **lower** |
| 1990 | 41.78160148 | 41.86087723 | 41.70232573 | 37.59087648 | 37.66573497 | 37.51601798 | 39.66680672 | 39.72120216 | 39.61241127 |
| 1991 | 42.33127782 | 42.41019848 | 42.25235716 | 38.14594799 | 38.22070269 | 38.0711933 | 40.21980351 | 40.2740992 | 40.16550782 |
| 1992 | 42.70103375 | 42.77946818 | 42.62259932 | 38.52005471 | 38.59446258 | 38.44564685 | 40.59250291 | 40.64651988 | 40.53848594 |
| 1993 | 42.98973264 | 43.06767801 | 42.91178726 | 38.80411034 | 38.87812109 | 38.7300996 | 40.87961591 | 40.93332273 | 40.82590909 |
| 1994 | 43.10671477 | 43.18397664 | 43.02945289 | 38.92286669 | 38.99627501 | 38.84945838 | 40.99811019 | 41.05136305 | 40.94485733 |
| 1995 | 43.20989575 | 43.28659861 | 43.13319289 | 38.99856503 | 39.0714473 | 38.92568277 | 41.08805656 | 41.14092685 | 41.03518628 |
| 1996 | 43.23298736 | 43.30904944 | 43.15692529 | 38.96526203 | 39.03749152 | 38.89303255 | 41.08432372 | 41.13673824 | 41.0319092 |
| 1997 | 43.34498269 | 43.42060107 | 43.26936432 | 38.97804564 | 39.04977786 | 38.90631343 | 41.14967506 | 41.20176042 | 41.0975897 |
| 1998 | 43.43846319 | 43.51363949 | 43.3632869 | 38.95491526 | 39.02613018 | 38.88370034 | 41.18810581 | 41.23985517 | 41.13635645 |
| 1999 | 43.54500988 | 43.61980197 | 43.47021778 | 38.9485097 | 39.01927643 | 38.87774296 | 41.24099571 | 41.29245307 | 41.18953835 |
| 2000 | 43.56066869 | 43.63495529 | 43.4863821 | 38.89750167 | 38.96775848 | 38.82724486 | 41.22512904 | 41.27622854 | 41.17402954 |
| 2001 | 43.58540027 | 43.65924155 | 43.51155898 | 38.87996471 | 38.94980044 | 38.81012898 | 41.23011921 | 41.28091302 | 41.1793254 |
| 2002 | 43.58656998 | 43.65995987 | 43.51318009 | 38.84802268 | 38.91743705 | 38.77860831 | 41.21663118 | 41.2671182 | 41.16614415 |
| 2003 | 43.59712269 | 43.67009384 | 43.52415154 | 38.82808891 | 38.89711123 | 38.75906658 | 41.21379934 | 41.26400154 | 41.16359714 |
| 2004 | 43.6361115 | 43.70870058 | 43.56352241 | 38.83236611 | 38.90102653 | 38.76370569 | 41.23678045 | 41.28672063 | 41.18684026 |
| 2005 | 43.71333285 | 43.78556784 | 43.64109787 | 38.8729437 | 38.94127247 | 38.80461493 | 41.29646708 | 41.34616597 | 41.24676819 |
| 2006 | 43.79856581 | 43.8704197 | 43.72671192 | 38.93955302 | 39.00754489 | 38.87156115 | 41.37277953 | 41.42222569 | 41.32333338 |
| 2007 | 43.85453678 | 43.92594634 | 43.78312721 | 39.00647507 | 39.07409644 | 38.9388537 | 41.43460412 | 41.4837634 | 41.38544485 |
| 2008 | 43.87672383 | 43.94762778 | 43.80581987 | 39.05574845 | 39.12295205 | 38.98854486 | 41.47067513 | 41.51950888 | 41.42184137 |
| 2009 | 43.88349574 | 43.95387551 | 43.81311597 | 39.09324249 | 39.1600111 | 39.02647388 | 41.49304877 | 41.5415441 | 41.44455343 |
| 2010 | 43.89176203 | 43.96165714 | 43.82186693 | 39.11693334 | 39.18328422 | 39.05058246 | 41.50903835 | 41.55721549 | 41.46086122 |
| 2011 | 43.91678514 | 43.98625168 | 43.8473186 | 39.12901869 | 39.1949758 | 39.06306158 | 41.52783223 | 41.57572022 | 41.47994424 |
| 2012 | 43.94810109 | 44.0171543 | 43.87904788 | 39.13481209 | 39.20038056 | 39.06924362 | 41.54709296 | 41.59469992 | 41.49948601 |
| 2013 | 43.98873901 | 44.05740225 | 43.92007577 | 39.14583734 | 39.21103726 | 39.08063742 | 41.5740229 | 41.62136435 | 41.52668146 |
| 2014 | 44.02860533 | 44.09689714 | 43.96031352 | 39.15986435 | 39.22471699 | 39.09501171 | 41.60219847 | 41.64928791 | 41.55510903 |
| 2015 | 44.05815175 | 44.12607268 | 43.99023082 | 39.17603799 | 39.24055904 | 39.11151694 | 41.62636237 | 41.67320526 | 41.57951948 |
| 2016 | 44.01452214 | 44.0820157 | 43.94702858 | 39.14409222 | 39.2082496 | 39.07993483 | 41.58970179 | 41.63626675 | 41.54313684 |
| 2017 | 43.90799024 | 43.97503395 | 43.84094654 | 39.06706478 | 39.13084551 | 39.00328406 | 41.49843674 | 41.54471081 | 41.45216268 |
| 2018 | 43.80796407 | 43.87460571 | 43.74132243 | 39.00642113 | 39.06987606 | 38.94296621 | 41.41813517 | 41.46415295 | 41.3721174 |
| 2019 | 43.78134721 | 43.84769126 | 43.71500316 | 39.02365778 | 39.08689406 | 38.9604215 | 41.41318473 | 41.45902042 | 41.36734905 |
| 2020 | 44.03763028 | 44.10396901 | 43.97129156 | 39.52542303 | 39.58887768 | 39.46196838 | 41.79114208 | 41.83704563 | 41.74523852 |
| 2021 | 44.74953882 | 44.81633657 | 44.68274106 | 40.10047396 | 40.16431212 | 40.0366358 | 42.43577542 | 42.4819599 | 42.38959095 |
| 2022 | 45.33645412 | 46.99368803 | 43.6792202 | 40.54970951 | 42.18731987 | 38.91209916 | 42.94179606 | 44.5731597 | 41.31043242 |
| 2023 | 45.5617394 | 47.63923568 | 43.48424313 | 40.78981312 | 42.81461541 | 38.76501082 | 43.1724326 | 45.20559594 | 41.13926925 |
| 2024 | 45.7625487 | 48.40338079 | 43.12171661 | 41.01257587 | 43.55794147 | 38.46721027 | 43.38212161 | 45.9550515 | 40.80919171 |
| 2025 | 45.93802603 | 49.25992715 | 42.61612491 | 41.21610038 | 44.39400193 | 38.03819883 | 43.56942796 | 46.79659524 | 40.34226069 |
| 2026 | 46.09083308 | 50.19354154 | 41.98812462 | 41.40069866 | 45.30701392 | 37.49438341 | 43.735745 | 47.71450955 | 39.75698045 |
| 2027 | 46.22443804 | 51.19532949 | 41.25354658 | 41.56875842 | 46.28803276 | 36.84948409 | 43.88396673 | 48.69982596 | 39.06810751 |
| 2028 | 46.34756036 | 52.26342059 | 40.43170014 | 41.72850164 | 47.33570185 | 36.12130143 | 44.02268609 | 49.75108033 | 38.29429186 |
| 2029 | 46.46805716 | 53.39967948 | 39.53643483 | 41.88703339 | 48.45180422 | 35.32226255 | 44.1594292 | 50.87014777 | 37.44871064 |
| 2030 | 46.59267987 | 54.60814073 | 38.57721901 | 42.05017512 | 49.63984611 | 34.46050414 | 44.30047329 | 52.06077343 | 36.54017314 |
| 2031 | 46.72676808 | 55.89348024 | 37.56005593 | 42.22228636 | 50.90371345 | 33.54085928 | 44.45063136 | 53.32717284 | 35.57408988 |
| 2032 | 46.87272804 | 57.25771961 | 36.48773647 | 42.40606674 | 52.24605307 | 32.56608042 | 44.61245201 | 54.67165188 | 34.55325215 |
| 2033 | 47.03506783 | 58.70395668 | 35.36617899 | 42.60660917 | 53.67120301 | 31.54201534 | 44.7908393 | 56.09804105 | 33.48363755 |
| 2034 | 47.21628356 | 60.23458125 | 34.19798588 | 42.82642124 | 55.18207202 | 30.47077047 | 44.9883249 | 57.60900684 | 32.36764297 |
| 2035 | 47.41593493 | 61.84966535 | 32.98220451 | 43.06487603 | 56.77890347 | 29.3508486 | 45.20434799 | 59.20464719 | 31.2040488 |
| 2036 | 47.6291092 | 63.54422337 | 31.71399504 | 43.31723268 | 58.45720912 | 28.17725624 | 45.43401527 | 60.88011381 | 29.98791672 |
| 2037 | 47.84849295 | 65.30958436 | 30.38740154 | 43.57669879 | 60.20923922 | 26.94415835 | 45.67022638 | 62.62711169 | 28.71334107 |
| 2038 | 48.06835504 | 67.13687741 | 28.99983267 | 43.83812034 | 62.02733168 | 25.64890899 | 45.90757754 | 64.43741502 | 27.37774007 |
| 2039 | 48.28248185 | 69.01673099 | 27.5482327 | 44.09542531 | 63.90272817 | 24.28812245 | 46.13991599 | 66.30193012 | 25.97790186 |
| 2040 | 48.48489841 | 70.94013609 | 26.02966073 | 44.3426373 | 65.82678063 | 22.85849397 | 46.36122808 | 68.21175305 | 24.51070311 |
| 2041 | 48.6705974 | 72.8992718 | 24.44192301 | 44.57479387 | 67.79204057 | 21.35754718 | 46.56648124 | 70.15915217 | 22.97381032 |
| 2042 | 48.83725063 | 74.88953212 | 22.78496914 | 44.78993045 | 69.79476598 | 19.78509492 | 46.75352463 | 72.1399129 | 21.36713635 |
| 2043 | 48.98846089 | 76.91351281 | 21.06340898 | 44.99165391 | 71.83813696 | 18.14517086 | 46.92603751 | 74.15700286 | 19.69507216 |
| 2044 | 49.12869654 | 78.97604619 | 19.28134689 | 45.1841458 | 73.92712038 | 16.44117123 | 47.0883725 | 76.21532917 | 17.96141582 |
| 2045 | 49.26185008 | 81.08210789 | 17.44159227 | 45.37113108 | 76.06693199 | 14.67533017 | 47.24433207 | 78.31993895 | 16.16872519 |
| 2046 | 49.38990182 | 83.2344833 | 15.54532034 | 45.55492981 | 78.26131225 | 12.84854737 | 47.39604362 | 80.4740179 | 14.31806934 |
| 2047 | 49.51303751 | 85.43320882 | 13.59286621 | 45.7367244 | 80.51232952 | 10.96111928 | 47.54418537 | 82.67856873 | 12.40980201 |
| 2048 | 49.63306237 | 87.67990488 | 11.58621985 | 45.91910758 | 82.82356624 | 9.014648909 | 47.69100062 | 84.93623276 | 10.44576848 |
| 2049 | 49.75093193 | 89.97542865 | 9.526435215 | 46.10343535 | 85.19726395 | 7.00960676 | 47.83764286 | 87.24852279 | 8.426762935 |
| 2050 | 49.86684524 | 92.31989874 | 7.413791734 | 46.29014771 | 87.63466226 | 4.945633165 | 47.98440491 | 89.61603595 | 6.352773872 |

Supplementary table18: Projection of ASPR.

| **year** | Male | | | Female | | | Both | | |
| --- | --- | --- | --- | --- | --- | --- | --- | --- | --- |
|  | **ASR** | **upper** | **lower** | **ASR** | **upper** | **lower** | **ASR** | **upper** | **lower** |
| 1990 | 309.7005255 | 309.9250622 | 309.4759887 | 281.3722602 | 281.5797809 | 281.1647396 | 295.1057482 | 295.2572685 | 294.9542279 |
| 1991 | 314.4344659 | 314.6582718 | 314.21066 | 285.2511363 | 285.4582513 | 285.0440213 | 299.4075292 | 299.5587253 | 299.256333 |
| 1992 | 317.9125552 | 318.1350454 | 317.690065 | 288.11418 | 288.3202795 | 287.9080805 | 302.5760747 | 302.726494 | 302.4256554 |
| 1993 | 320.5968402 | 320.8178634 | 320.375817 | 290.3701642 | 290.5750987 | 290.1652297 | 305.0459764 | 305.1955048 | 304.8964481 |
| 1994 | 321.935022 | 322.1541041 | 321.7159398 | 291.5412276 | 291.7445569 | 291.3378982 | 306.3020898 | 306.4503989 | 306.1537808 |
| 1995 | 322.734194 | 322.9514481 | 322.51694 | 292.1978784 | 292.3996846 | 291.9960722 | 307.0349841 | 307.1821493 | 306.887819 |
| 1996 | 322.8686598 | 323.0839312 | 322.6533884 | 292.3166469 | 292.5167205 | 292.1165733 | 307.1685269 | 307.3144122 | 307.0226416 |
| 1997 | 323.4962184 | 323.7099654 | 323.2824715 | 292.9460416 | 293.1447769 | 292.7473062 | 307.8069412 | 307.9518446 | 307.6620378 |
| 1998 | 324.0173149 | 324.2295752 | 323.8050546 | 293.4804797 | 293.6778869 | 293.2830726 | 308.3435369 | 308.4874691 | 308.1996046 |
| 1999 | 324.5606544 | 324.7714929 | 324.3498159 | 293.9908013 | 294.1869687 | 293.794634 | 308.8790027 | 309.0220193 | 308.7359861 |
| 2000 | 324.5958792 | 324.8050066 | 324.3867519 | 294.0185891 | 294.2133307 | 293.8238475 | 308.9209588 | 309.0628984 | 308.7790192 |
| 2001 | 324.7828824 | 324.9904038 | 324.5753611 | 293.966115 | 294.1594955 | 293.7727346 | 309.0035291 | 309.1444561 | 308.8626021 |
| 2002 | 325.0632408 | 325.269223 | 324.8572586 | 293.7894283 | 293.9814347 | 293.5974219 | 309.0772259 | 309.2171638 | 308.937288 |
| 2003 | 325.4573421 | 325.6618985 | 325.2527856 | 293.6103033 | 293.8009871 | 293.4196196 | 309.2067065 | 309.3457108 | 309.0677021 |
| 2004 | 325.9367583 | 326.1399435 | 325.7335731 | 293.4851614 | 293.6745541 | 293.2957688 | 309.4005125 | 309.5386081 | 309.2624168 |
| 2005 | 326.4173842 | 326.6191872 | 326.2155812 | 293.4831872 | 293.6713398 | 293.2950345 | 309.6507113 | 309.7879139 | 309.5135088 |
| 2006 | 326.6255175 | 326.8257754 | 326.4252595 | 293.4184905 | 293.6053301 | 293.2316508 | 309.7341073 | 309.8703357 | 309.597879 |
| 2007 | 326.4372819 | 326.6358074 | 326.2387563 | 293.1364074 | 293.3218106 | 292.9510043 | 309.5155732 | 309.6507199 | 309.3804265 |
| 2008 | 325.9166735 | 326.1133392 | 325.7200079 | 292.6773401 | 292.8612169 | 292.4934633 | 309.0428487 | 309.1768345 | 308.9088629 |
| 2009 | 325.2115941 | 325.4063425 | 325.0168458 | 292.1768325 | 292.3591596 | 291.9945055 | 308.4528564 | 308.5856497 | 308.320063 |
| 2010 | 324.4737409 | 324.6666641 | 324.2808177 | 291.7491212 | 291.9299952 | 291.5682473 | 307.8735341 | 308.0051982 | 307.7418701 |
| 2011 | 323.4585485 | 323.6496495 | 323.2674475 | 291.0863678 | 291.2657715 | 290.906964 | 307.0330522 | 307.1635788 | 306.9025256 |
| 2012 | 322.0091675 | 322.1983373 | 321.8199977 | 290.0207188 | 290.1985406 | 289.842897 | 305.7725879 | 305.9018987 | 305.6432771 |
| 2013 | 320.4711118 | 320.658386 | 320.2838377 | 288.8479908 | 289.0242411 | 288.6717404 | 304.4153851 | 304.5434931 | 304.287277 |
| 2014 | 319.1198157 | 319.3053134 | 318.9343181 | 287.8037384 | 287.9785021 | 287.6289747 | 303.2166326 | 303.343605 | 303.0896602 |
| 2015 | 318.2198964 | 318.4038019 | 318.0359909 | 287.1410947 | 287.3145492 | 286.9676403 | 302.4388746 | 302.5648402 | 302.3129091 |
| 2016 | 317.3130938 | 317.4954076 | 317.1307799 | 286.3471364 | 286.5192713 | 286.1750015 | 301.5956322 | 301.7205909 | 301.4706735 |
| 2017 | 316.1765477 | 316.3572497 | 315.9958458 | 285.1837271 | 285.3544778 | 285.0129763 | 300.4496556 | 300.5735771 | 300.3257341 |
| 2018 | 315.195991 | 315.3752104 | 315.0167717 | 284.1731641 | 284.3426499 | 284.0036783 | 299.4571148 | 299.5800857 | 299.3341439 |
| 2019 | 314.728632 | 314.9066024 | 314.5506617 | 283.8245737 | 283.9930699 | 283.6560774 | 299.0527827 | 299.1749818 | 298.9305835 |
| 2020 | 316.529203 | 316.7067572 | 316.3516487 | 287.2505911 | 287.4192656 | 287.0819167 | 301.6545177 | 301.7766308 | 301.5324046 |
| 2021 | 320.9987895 | 321.1768477 | 320.8207312 | 292.5490089 | 292.7186218 | 292.3793961 | 306.5346841 | 306.6573018 | 306.4120664 |
| 2022 | 322.0510238 | 332.9442352 | 311.1578124 | 292.250363 | 302.8223114 | 281.6784145 | 306.8185042 | 317.5115123 | 296.125496 |
| 2023 | 323.0972129 | 337.0479474 | 309.1464784 | 293.8228926 | 307.3517496 | 280.2940356 | 308.128477 | 321.8355759 | 294.4213781 |
| 2024 | 324.0040193 | 341.9936193 | 306.0144193 | 295.3052489 | 312.7626892 | 277.8478085 | 309.323765 | 327.0256834 | 291.6218466 |
| 2025 | 324.7714844 | 347.586409 | 301.9565598 | 296.692949 | 318.8730367 | 274.5128613 | 310.4017846 | 332.8925258 | 287.9110433 |
| 2026 | 325.4125016 | 353.7112756 | 297.1137275 | 297.9825586 | 325.5582928 | 270.4068244 | 311.3669612 | 339.3157965 | 283.418126 |
| 2027 | 325.9329065 | 360.2871235 | 291.5786894 | 299.1678146 | 332.7279279 | 265.6077012 | 312.2186702 | 346.2088605 | 278.22848 |
| 2028 | 326.3834039 | 367.2881924 | 285.4786155 | 300.2968918 | 340.358247 | 260.2355367 | 313.0069615 | 353.5471086 | 272.4668144 |
| 2029 | 326.8093308 | 374.7162253 | 278.9024364 | 301.4110709 | 348.4499846 | 254.3721571 | 313.77521 | 361.3312893 | 266.2191307 |
| 2030 | 327.2491238 | 382.589466 | 271.9087817 | 302.5419538 | 357.0161396 | 248.067768 | 314.5583418 | 369.5764218 | 259.5402618 |
| 2031 | 327.7307473 | 390.9294562 | 264.5320383 | 303.7069718 | 366.0678507 | 241.3460929 | 315.3791214 | 378.2985619 | 252.4596809 |
| 2032 | 328.2570862 | 399.7320949 | 256.7820775 | 304.90399 | 375.5967333 | 234.2112467 | 316.2377917 | 387.4910675 | 244.9845158 |
| 2033 | 328.8592562 | 409.013964 | 248.7045485 | 306.1676974 | 385.6275221 | 226.7078726 | 317.1678609 | 397.1751768 | 237.1605451 |
| 2034 | 329.5546805 | 418.7881527 | 240.3212082 | 307.5178929 | 396.1795265 | 218.8562592 | 318.1879945 | 407.3670314 | 229.0089577 |
| 2035 | 330.33945 | 429.0513527 | 231.6275472 | 308.9536769 | 407.2556776 | 210.6516761 | 319.2956238 | 418.0660971 | 220.5251504 |
| 2036 | 331.1754753 | 439.7608117 | 222.590139 | 310.4439494 | 418.8240095 | 202.0638892 | 320.4560254 | 429.2346801 | 211.6773708 |
| 2037 | 332.0094278 | 450.8485581 | 213.1702976 | 311.9402366 | 430.8248129 | 193.0556604 | 321.6179655 | 440.8083279 | 202.4276031 |
| 2038 | 332.8176771 | 462.2680001 | 203.3673542 | 313.4280162 | 443.2277029 | 183.6283295 | 322.762622 | 452.7487249 | 192.7765192 |
| 2039 | 333.578028 | 473.9782221 | 193.1778339 | 314.8894185 | 456.0016343 | 173.7772027 | 323.8697866 | 465.019444 | 182.7201291 |
| 2040 | 334.2701748 | 485.944313 | 182.5960366 | 316.3045724 | 469.1158599 | 163.4932848 | 324.919046 | 477.5869575 | 172.2511344 |
| 2041 | 334.8752178 | 498.134784 | 171.6156515 | 317.6515548 | 482.5376406 | 152.7654689 | 325.8896921 | 490.4184755 | 161.3609087 |
| 2042 | 335.3726854 | 510.5123677 | 160.2330032 | 318.9075653 | 496.2290736 | 141.5860569 | 326.7599075 | 503.4758906 | 150.0439244 |
| 2043 | 335.7845523 | 523.0901327 | 148.4789719 | 320.0956533 | 510.2097134 | 129.9815932 | 327.5526349 | 516.775985 | 138.3292847 |
| 2044 | 336.1376499 | 535.8957553 | 136.3795444 | 321.2405834 | 524.5088967 | 117.9722701 | 328.293783 | 530.3472923 | 126.2402738 |
| 2045 | 336.4565968 | 548.9612215 | 123.9519722 | 322.3627993 | 539.1563484 | 105.5692501 | 329.0058214 | 544.2204398 | 113.791203 |
| 2046 | 336.7551105 | 562.3071411 | 111.2030799 | 323.4711962 | 554.1683406 | 92.77405185 | 329.6999188 | 558.4135129 | 100.9863247 |
| 2047 | 337.0252166 | 575.9192983 | 98.13113492 | 324.5573937 | 569.5323936 | 79.58239388 | 330.3677937 | 572.9127879 | 87.82279944 |
| 2048 | 337.2779736 | 589.804993 | 84.75095423 | 325.6386934 | 585.2703503 | 66.00703655 | 331.0239812 | 587.7332408 | 74.31472171 |
| 2049 | 337.5205056 | 603.9699072 | 71.07110396 | 326.725749 | 601.3982197 | 52.05327825 | 331.6773628 | 602.885515 | 60.46921066 |
| 2050 | 337.7564216 | 618.418255 | 57.09458815 | 327.8226323 | 617.9252013 | 37.72006331 | 332.331597 | 618.3758005 | 46.28739342 |

Supplementary table19: Baseline regressions of GCAE.

| val | DALYs | | | Mortality | | | YLLs | | | Incidence | | |
| --- | --- | --- | --- | --- | --- | --- | --- | --- | --- | --- | --- | --- |
|  | Male | Female | Both | Male | Female | Both | Male | Female | Both | Male | Female | Both |
| Coef. | -0.05 | -0.07 | -0.06 | 0.00 | 0.00 | 0.00 | -0.03 | -0.03 | -0.03 | 0.00 | -0.01 | 0.00 |
| Std.Crr | 0.01 | 0.00 | 0.00 | 0.00 | 0.00 | 0.00 | 0.00 | 0.00 | 0.00 | 0.00 | 0.00 | 0.00 |
| t | -9.92 | -15.43 | -12.44 | -6.18 | -7.12 | -6.33 | -6.26 | -8.59 | -6.87 | -3.20 | -12.92 | -8.07 |
| P>\|t\| | 0.00 | 0.00 | 0.00 | 0.00 | 0.00 | 0.00 | 0.00 | 0.00 | 0.00 | 0.00 | 0.00 | 0.00 |
| 95% Coef. | -0.06 | -0.08 | -0.07 | 0.00 | 0.00 | 0.00 | -0.04 | -0.04 | -0.03 | 0.00 | -0.01 | -0.01 |
| Interval | -0.04 | -0.06 | -0.05 | 0.00 | 0.00 | 0.00 | -0.02 | -0.02 | -0.02 | 0.00 | -0.01 | 0.00 |
